# Discovery of *N*,4-Di(1*H*-pyrazol-4-yl)pyrimidin-2-amine-Derived CDK2 Inhibitors as Potential Anticancer Agents: Design, Synthesis, and Evaluation

**DOI:** 10.3390/molecules28072951

**Published:** 2023-03-25

**Authors:** Biruk Sintayehu Fanta, Jimma Lenjisa, Theodosia Teo, Lianmeng Kou, Laychiluh Mekonnen, Yuchao Yang, Sunita K. C. Basnet, Ramin Hassankhani, Matthew J. Sykes, Mingfeng Yu, Shudong Wang

**Affiliations:** Drug Discovery and Development, Clinical and Health Sciences, University of South Australia, Adelaide, SA 5000, Australia

**Keywords:** CDK2, CDK2 inhibitor, pyrazole, *N*,4-di(1*H*-pyrazol-4-yl)pyrimidin-2-amine, antiproliferative activity, bioisosteric replacement

## Abstract

Cyclin-dependent kinase 2 (CDK2) has been garnering considerable interest as a target to develop new cancer treatments and to ameliorate resistance to CDK4/6 inhibitors. However, a selective CDK2 inhibitor has yet to be clinically approved. With the desire to discover novel, potent, and selective CDK2 inhibitors, the phenylsulfonamide moiety of our previous lead compound **1** was bioisosterically replaced with pyrazole derivatives, affording a novel series of *N*,4-di(1*H*-pyrazol-4-yl)pyrimidin-2-amines that exhibited potent CDK2 inhibitory activity. Among them, **15** was the most potent CDK2 inhibitor (*K*_i_ = 0.005 µM) with a degree of selectivity over other CDKs tested. Meanwhile, this compound displayed sub-micromolar antiproliferative activity against a panel of 13 cancer cell lines (GI_50_ = 0.127–0.560 μM). Mechanistic studies in ovarian cancer cells revealed that **15** reduced the phosphorylation of retinoblastoma at Thr821, arrested cells at the S and G2/M phases, and induced apoptosis. These results accentuate the potential of the *N*,4-di(1*H*-pyrazol-4-yl)pyrimidin-2-amine scaffold to be developed into potent and selective CDK2 inhibitors for the treatment of cancer.

## 1. Introduction

Cyclin-dependent kinase 2 (CDK2) is a serine/threonine protein kinase that is activated by binding to cyclin A or E and phosphorylation at its Thr160 residue by the CDK-activating kinase (CAK, i.e., CDK7-cyclin H-MAT1). The activity of CDK2 is also negatively regulated by phosphorylation at its Thr14 and Tyr15 by Wee1/Myt1 as well as by binding to CDK inhibitory proteins such as the CDK-interacting protein (Cip)/kinase inhibitory protein (Kip) family members (i.e., p21^Cip1^, p27^Kip1^, and p57^Kip2^) [1]. CDK2 plays important roles in overseeing various facets of the cell division cycle, including regulation of the G1-to-S transition, centrosome duplication, DNA replication and repair, and activation of CDK1/cyclin B for the G2-to-M transition [2,3]. Aberrant activity of CDK2 is linked with a myriad of human cancer types, including those originating from the ovary [4], breast [5], lung [6], and brain [1]. Moreover, this kinase is associated with resistance to CDK4/6 inhibitors [7,8]. Therefore, CDK2 has been an attractive target for the development of new anticancer treatments and the amelioration of resistance to CDK4/6 inhibitors [9]. Indeed, several studies have demonstrated the potential of CDK2 inhibition in cancer treatments both as a monotherapy and as part of combination therapies [9,10,11,12].

Structurally-diverse small-molecule CDK2 inhibitors have been developed, including AT7519, CYC065, dinaciclib, PF-06873600, and PF-07104091, all of which are currently at different stages of clinical trials, however, there is not yet any clinically-approved selective CDK2 inhibitor [1,13]. Consequently, it is imperative to develop novel, potent, and selective CDK2 inhibitors.

**Figure 1:**
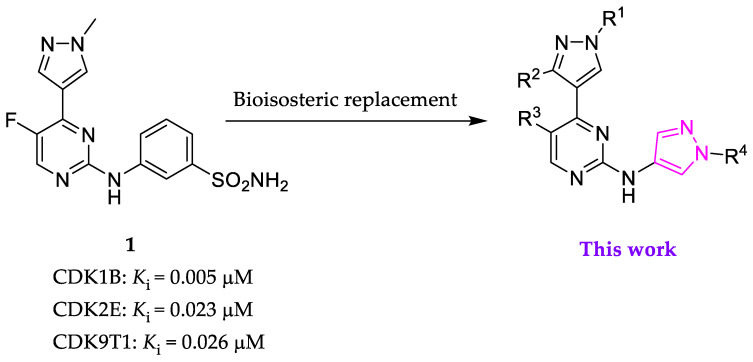
Design of novel CDK2 inhibitors from our previously-identified **1** [20].

With our continued aspiration to discover potent and selective CDK inhibitors [14,15,16,17,18,19,20], we employed a bioisosteric replacement strategy to develop a new chemotype of CDK2 inhibitors from our previously-identified compound, 3-((5-fluoro-4-(1-methyl-1*H*-pyrazol-4-yl)pyrimidin-2-yl)amino)benzenesulfonamide **1** (Figure 1). This compound inhibited multiple CDKs potently, particularly CDKs 1, 2, and 9. Pyrazole is a privilege scaffold that features in many drugs, including the anticancer kinase inhibitors such as crizotinib, erdafitinib, encorafenib, and pralsetinib [21]. This scaffold has also been commonly used as a bioisostere during lead optimization [22,23,24]. A search of the SwissBioisostere database returned that pyrazole has been utilized as a bioisosteric replacement of the benzene ring 263 times, and >80% (214 times) of instances resulted in positive outcomes (i.e., better or similar biological activities for 70 and 144 times, respectively) (http://www.swissbioisostere.ch accessed on 27 November 2022) [25,26]. Encouraged by these outcomes, we designed and synthesized *N*,4-di(1*H*-pyrazol-4-yl)pyrimidin-2-amine derivatives by substituting the phenylsulfonamide moiety of **1** with a pyrazole motif. Herein, we report the preparation and evaluation of *N*,4-di(1*H*-pyrazol-4-yl)pyrimidin-2-amine derivatives as potential CDK2 inhibitors.

## 2. Results and Discussion

### 2.1. Inhibitor Design

With the desire to discover potent and selective CDK2 inhibitors with drug-like properties, we used a bioisosteric replacement strategy, i.e., substitution of the phenylsulfonamide moiety of **1**, our recently-reported lead compound [20], with a wide range of pyrazole-derived groups to conceive a series of *N*,4-di(1*H*-pyrazol-4-yl)pyrimidin-2-amine analogues (Figure 1). We envisioned that this replacement might improve the potency, selectivity, and physiochemical properties. Moreover, the N-NH motif of the newly introduced pyrazole ring could serve as both a hydrogen-bond acceptor and donor to interact with CDK2, and such an interaction might contribute positively to the potency of the entire molecule [27,28].

**Figure 2:**
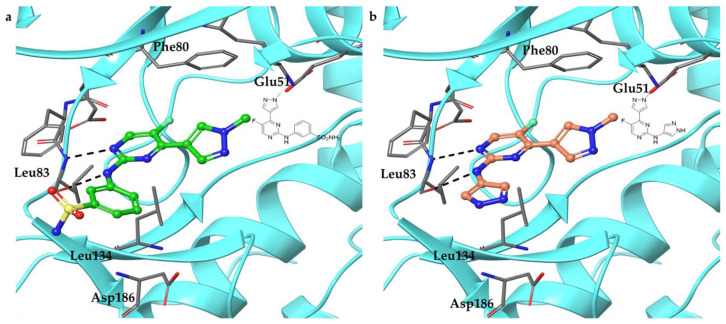
Predicted binding pose of **1** (**a**) or **14** (**b**) in CDK2/cyclin E (PDB ID: 7KJS). The kinase is presented in cyan ribbons with selected amino acids annotated, while **1** and **14** are colored green and salmon, respectively. Hydrogen bonds are indicated by dashed black lines. The illustration was generated using Maestro Schrödinger Release 2021-1.

**Scheme 1:**
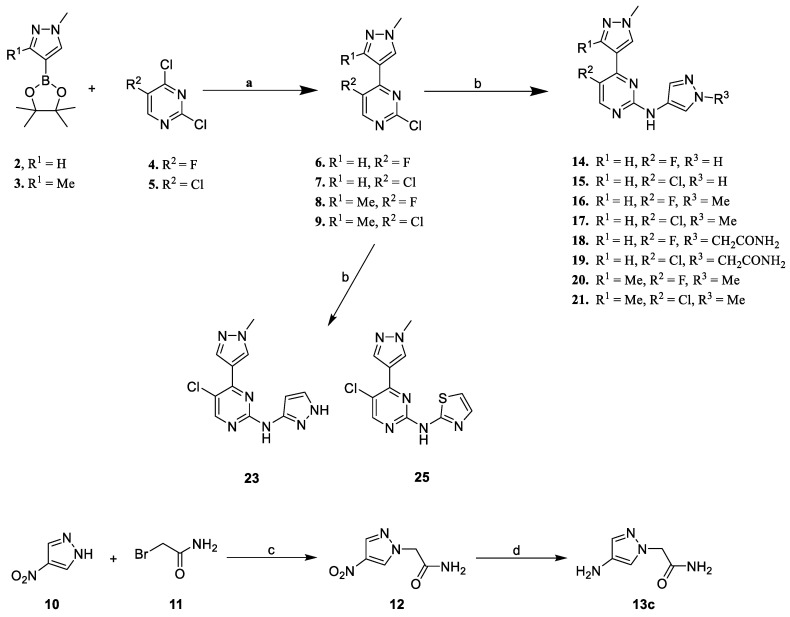
Synthesis of *N*,4-di(1*H*-pyrazolyl)pyrimidin-2-amines **14**–**21** and **23** and *N*-(5-chloro-4-(1-methyl-1*H*-pyrazol-4-yl)pyrimidin-2-yl)thiazol-2-amine **25**. Reagents and conditions: (a) PdCl_2_(dppf)·DCM, K_2_CO_3_, 1,4-dioxane/ethanol/water (*v/v/v*, 7/3/4), 80 °C, 12 h, and 59–69%; (b) appropriate aminopyrazoles (**13a**–**13c** and **22**) or thiazol-2-amine (**24**), Pd_2_(dba)_3_, Xantphos, Cs_2_CO_3_, 1,4-dioxane, microwave 200–300 W, 140 °C, 1 h, and 13–28%; (c) K_2_CO_3_, acetonitrile, 80 °C, o/n, and 95%; and (d) H_2_, 10% Pd/C, methanol, rt, o/n, and 90%.

To understand the potential of these newly designed compounds to inhibit CDK2, a molecular modeling study on **14**, an exemplar of the proposed series (Scheme 1), or **1** in complex with CDK2/cyclin E was performed, and their respective plausible binding modes are shown in Figure 2. Both compounds are predicted to engage the hinge region, forming hydrogen bonds through their common pyrimidinyl-N1-C2-NH motif with Leu83 of CDK2. These highly similar CDK2-binding features between **14** and **1** encouraged us to synthesize the *N*-pyrazole analogues. 

### 2.2. Synthesis

Scheme 1 depicts the synthesis of *N*,4-di(1*H*-pyrazolyl)pyrimidin-2-amines **14–21** and **23** and *N*-(5-chloro-4-(1-methyl-1*H*-pyrazol-4-yl)pyrimidin-2-yl)thiazol-2-amine **25**. Suzuki coupling reactions of either (1-methyl-1*H*-pyrazol-4-yl)boronic acid pinacol ester **2** or its pyrazolyl-C3-methylated counterpart **3** with a 5-substituted-2,4-dichloropyrimidine **4** or **5** in the presence of PdCl_2_(dppf)·DCM and K_2_CO_3_ in 1,4-dioxane/ethanol/water afforded 2-chloro-4-(1-methyl-1*H*-pyrazol-4-yl)pyrimidines **6–9** in 59–69% yields. Buchwald–Hartwig amination of these chlorides with appropriate aminopyrazoles **13a–13c** and **22** or thiazol-2-amine **24** in the presence of Pd_2_(dba)_3_, xantphos, and Cs_2_CO_3_, yielded the desired compounds (**14–21**, **23**, and **25**) in varying yields (13–28%). Compounds **13a** and **13b** were purchased, whereas **13c** was prepared in-house (Scheme 1).

**Scheme 2:**
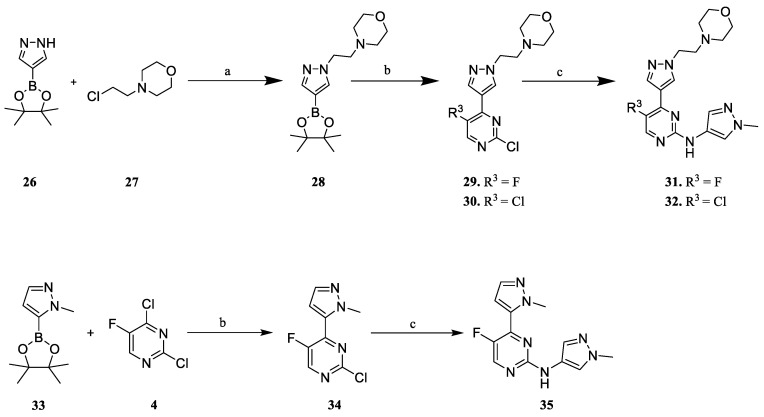
Synthesis of **31**, **32**, and **35**. Reagents and conditions: (a) Cs_2_CO_3_, acetonitrile, reflux, 24 h, and 57%; (b) **4** or **5**, PdCl_2_(dppf)·DCM, K_2_CO_3_, 1,4-dioxane/ethanol/water (*v*/*v*/*v*, 7/3/4), 80 °C, 12 h, and 40–45%; and (c) 1-methyl-1*H*-pyrazol-4-amine (**13b**), Pd_2_(dba)_3_, Xantphos, Cs_2_CO_3_, 1,4-dioxane, microwave 200–300 W, 140 °C, 1 h, and 16–26%.

Scheme 2 illustrates the preparation of **31**, **32**, and **35**. Compound **28** was prepared in moderate yield (57%) from (1*H*-pyrazol-4-yl)boronic acid pinacol ester **26** and 4-(2-chloroethyl)morpholine **27** in the presence of Cs_2_CO_3_ in acetonitrile at reflux. Suzuki coupling reactions of **28** with **4** or **5** afforded **29** and **30** which each were subsequently aminated with 1-methyl-1*H*-pyrazol-4-amine **13b** under Buchwald–Hartwig coupling conditions to afford **31** and **32** in yields of 17% and 16%, respectively. 

To explore the effect of regioisomerism of the pyrazole ring at the pyrimidinyl-C4 position on the CDK2 inhibition, 5-fluoro-*N*-(1-methyl-1*H*-pyrazol-4-yl)-4-(1-methyl-1*H*-pyrazol-5-yl)pyrimidin-2-amine **35** was synthesized (Scheme 2). Suzuki coupling reaction of (1-methyl-1*H*-pyrazol-5-yl)boronic acid pinacol ester **33** with 2,4-dichloro-5-fluoropyrimidine **4** gave 2-chloro-5-fluoro-4-(1-methyl-1*H*-pyrazol-5-yl)pyrimidine **34** in 42% yield. Subsequent Buchwald–Hartwig amination of **34** with **13b** produced **35** in a yield of 26%. 

### 2.3. Structure-Activity Relationships

With the desire to improve the potency and selectivity of our previously-identified lead compound **1 [20]**, its phenylsulfonamide moiety was replaced with pyrazole-derived groups, affording a series of *N*,4-di(1*H*-pyrazol-4-yl)pyrimidin-2-amine derivatives. These new analogues were then evaluated for their activities against CDK2E; in order to understand their selectivity profiles, they were also tested against other CDKs, namely CDK1B, CDK5p25, and CDK9T1, all of which share high amino acid sequence similarity with CDK2E. Additionally, the antiproliferative activities of these compounds were screened against A2780 ovarian cancer cells.

As shown in Table 1, substitution of the phenylsulfonamide moiety of **1** with an unsubstituted pyrazol-4-yl ring gave **14**, which displayed more potent CDK2 and CDK5 inhibition with *K*_i_ values of 0.007 and 0.003 µM, respectively. Compound **14** also improved the selectivity of **1** for CDK2 over CDKs 1 and 9. However, **14** showed about 28-fold lower antiproliferative activity against A2780 ovarian cancer cells when compared to **1**. Replacement of the fluorine atom in **14** (R^2^ = F) with chlorine afforded **15** (R^2^ = Cl), which maintained the inhibitory potencies towards CDK2 (*K*_i_ = 0.005 µM) and CDK5 (*K*_i_ = 0.003 µM) and slightly enhanced the selectivity for CDK2 over CDK1 and CDK9. Furthermore, in comparison with **1**, **15** showed improved potency against and selectivity for CDK2 but reduced antiproliferative activity against A2780 cells (**15**: GI_50_ = 0.158 µM versus **1**: GI_50_ = 0.018 µM). Encouraged by these results (i.e., enhanced CDK2 inhibitory activity and selectivity), further SAR studies were carried out by varying R^1^ and R^3^ substituents while retaining R^2^ as chlorine or fluorine.

Substitution of the *N*-pyrazol-4-yl-NH in **14** and **15** (R^3^ = H) by a methyl, affording **16** and **17** (R^3^ = Me), respectively, led to a reduction in the inhibitory potencies across all the CDKs tested and, consequently, resulted in diminished antiproliferative activities against A2780 cells. An acetamide group was then introduced at the same position to test whether the terminal amide could interact with Asp86 and/or Lys89 residues of CDK2. However, **18** and **19** (R^3^ = CH_2_CONH_2_) thus obtained had detrimental effects on the inhibition of all four CDKs and A2780 cells. Taken together, these results suggest the importance of the unmasked pyrazole ring (R^3^ = H) at the pyrimidinyl-C2-NH position for inhibitory activities towards both CDK2 and A2780 cells. 

We next investigated the SAR around the other pyrazole ring occupying the pyrimidinyl-C4 position (i.e., R and R^1^). As shown in Table 2, methylation of the pyrazolyl-C3 position as in **20** and **21** (R^1^ = Me) led to significant decreases in inhibitory activities against all the CDKs tested with concomitant reductions in the cellular activities. Similarly, substitution of the pyrazolyl-N1-methyl group by a larger 2-morpholinoethyl as in **31** and **32** (R = 2-morpholinoethyl) had detrimental effects on both kinase and cellular inhibition (e.g., **32**: *K*_i_(CDK2) = 0.252 µM and %GI(A2780) = 3 at 1 µM versus **17**: *K*_i_(CDK2) = 0.011 µM and %GI(A2780) = 87 at 1 µM (not shown in Table 1)), thus signifying that diversification of the pyrazole ring by introduction of a larger substituent at the N1 or C3 position may not be tolerated. To explore the effect of regioisomerism regarding the pyrazole ring at the pyrimidinyl-C4 position on the CDK2 inhibition, **35** with a 1-methyl-1*H*-pyrazol-5-yl moiety was synthesized and found to be less active towards CDK2 than its 1-methyl-1*H*-pyrazol-4-yl counterpart **16** (% CDK2 inhibition = 64 and 97 (not shown in Table 1), respectively), suggesting the dependency of the kinase inhibition on the topology of the pyrazole ring at the pyrimidinyl-C4 position.

Having identified the dependence of the CDK2 inhibitory activity on the topology of the 1-methyl-1*H*-pyrazol-4-yl group at the pyrimidinyl-C4 position, it seemed logical to investigate if a similar dependence existed on the other pyrazole ring at the pyrimidinyl-C2-NH position. As a result, 5-chloro-4-(1-methyl-1*H*-pyrazol-4-yl)-*N*-(1*H*-pyrazol-5-yl)pyrimidin-2-amine **23** was synthesized. As Table 3 shows, the compound displayed greatly diminished CDK inhibitory activities, particularly with an 18-fold reduced CDK2 inhibition when compared to its 1*H*-pyrazol-4-yl counterpart **15** (*K*_i_ = 0.090 and 0.005 µM, respectively). Consequently, **23** showed 47-fold less potent antiproliferative activity against A2780 cells than did **15** (GI_50_ = 7.350 and 0.158 µM, respectively), indicating the dependency of the CDK2 inhibitory activity on the orientation of the 1*H*-pyrazolyl ring at the pyrimidinyl-C2-NH position.

Next, we investigated the effect of a related heteroaromatic system—thiazol-2-yl—at the pyrimidinyl-C2-NH position (i.e., **25**) on the CDK and A2780 inhibitory activities. As shown in Table 3, the introduction of such a ring led to a significant reduction of the inhibitory activities toward all the CDKs tested as well as A2780 cells. 

Taken together, compounds with chlorine at the pyrimidinyl-C5 position (R^2^ = Cl) were generally more potent CDK2 inhibitors than their fluorine counterparts (R^2^ = F), which may be attributed to a stronger hydrophobic interaction between the chlorine and Phe80.

### 2.4. Docking Study on ***15***

**Figure 3:**
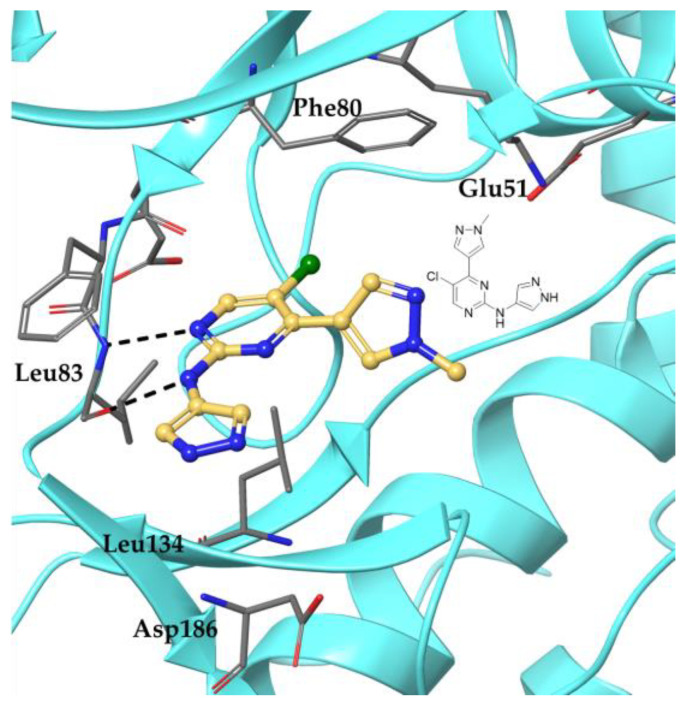
Compound **15** docked into CDK2/cyclin E (PDB ID: 7KJS). The kinase is presented in cyan ribbons with selected amino acids annotated, while **15** is colored yellow. Hydrogen bonds are indicated by dashed black lines. The illustration was generated using Maestro Schrödinger Release 2021-1.

To understand the likely binding mode of **15**, the most potent CDK2 inhibitor in the series, with CDK2 and to provide a rationale for the observed potency, the compound was docked into the crystal structure of CDK2/cyclin E (PDB ID: 7KJS) using OEDocking 4.1.2.1 from OpenEye Scientific Software. The result revealed that **15** may form two hydrogen bonds with Leu83 in the ATP binding pocket of CDK2 (Figure 3). Specifically, while the 2-amino group of the pyrimidine ring could develop a hydrogen bond with the carbonyl of Leu83, the pyrimidinyl-N1 is likely to form a second hydrogen bond with the NH of the same amino acid residue. This binding pose is similar to those of **1** or **14** in complex with CDK2/cyclin E (Figure 2) and those of other inhibitors co-crystallized with CDK2 [15,27,29]. 

### 2.5. Antiproliferative Activities of ***15***

Based on the most potent CDK2 inhibition displayed by **15** (*K*_i_ = 0.005 µM), it was selected for further antiproliferative studies using various types of cancer cell lines, including leukemia (MV4-11 and U937), breast (MCF7 (Rb-positive) and MDA-MB-231(Rb-negative)), and ovarian (OVCAR5, OAW28, OV90, Cov318, and Cov504) cancers, and glioblastoma (U87, T98G, and U251). As Table 4 shows, **15** inhibited the proliferation of all the cancer cell lines tested at submicromolar concentrations, with GI_50_ values ranging from 0.127 to 0.560 µM. The most sensitive cell line to **15** (GI_50_ = 0.127 µM) was MV4-11, which was followed by the ovarian cancer cell line OVCAR5 (GI_50_ = 0.150 µM). 

### 2.6. Cellular Mechanistic Studies of ***15***

Given that A2780 and OVCAR5 were two of the most sensitive cancer cell lines to **15** in the above cancer cell panel screen, both ovarian cell lines were selected for cellular mechanistic studies. The effects of **15** on cell cycle progression and the level of phosphorylated Rb were determined by flow cytometry and Western blotting, respectively. In addition, the impacts of **15** on colony formation and apoptosis were analyzed by staining with crystal violet and annexin V/propidium iodide (PI), respectively. CYC065, a CDK2/9 inhibitor and a clinical candidate [30], was used as a control in all mechanistic studies conducted. 

#### 2.6.1. Effect of **15** on the Cell Cycle Progression

**Figure 4:**
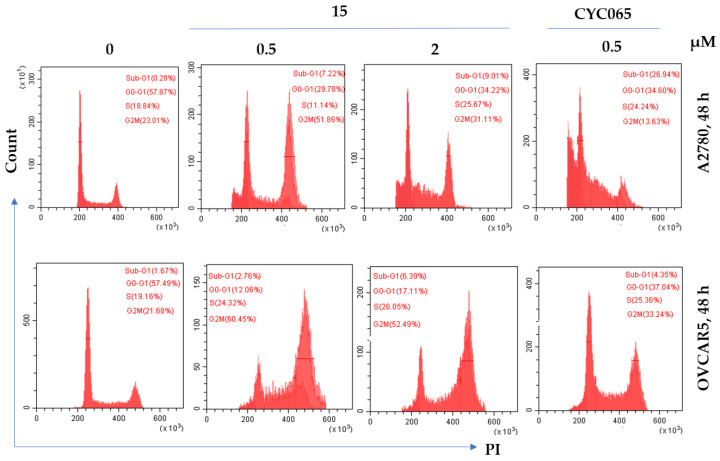
Effects of **15** on the cell cycle progression of A2780 and OVCAR5 ovarian cancer cells. Cells were incubated with **15** at 0.5 or 2 µM or CYC065 at 0.5 µM for 48 h. DMSO diluent was employed as a control. The experiments were performed twice, and representative data are presented.

A2780 cells were treated with **15** at concentrations of 0.5 or 2 µM for a period of 48 h. The flow cytometric analysis (Figure 4; top) showed that the compound at 0.5 µM arrested the cells at the G2/M phase (about 52%) when compared to the untreated cells (23%). An increased subpopulation of S-phase cells was also observed with the treatment of 2 µM of **15** (26% versus 19% in untreated cells). At 0.5 µM, CYC065 caused the cell to arrest in sub-G1 (27% versus 0.3% in untreated cells) and S-phase (24% versus 19% in untreated cells). 

Similar cell-cycle effects on OVCAR5 cells were also observed (Figure 4, bottom). At the concentration of 0.5 µM, **15** resulted in a 39% increment in the subpopulation of G2/M cells when compared to the vehicle control. At 2 µM, the compound also showed a similar effect (53% G2/M-phase cells versus 22% in untreated cells) with an increased S-phase subpopulation (26% versus 19% in untreated cells). CYC065 caused a similar cell-cycle profile with an increased percentage of G2/M cells, but to a lesser extent (a 12% increase when compared to the vehicle control). 

#### 2.6.2. Effect of **15** on Apoptosis in Cancer Cells

**Figure 5:**
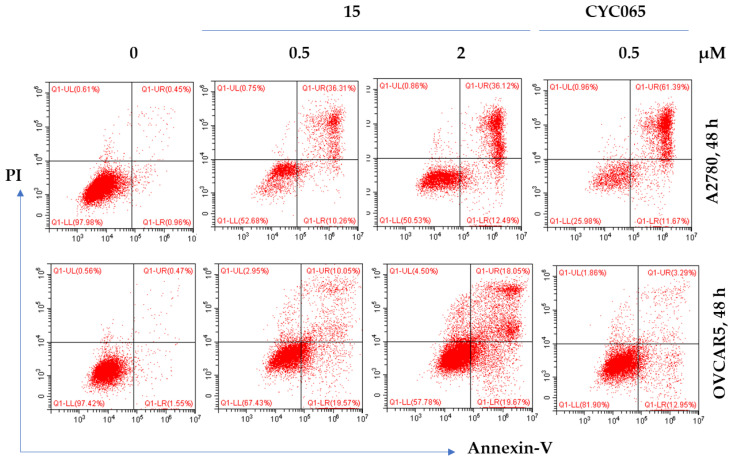
Effect of **15** on apoptosis in A2780 and OVCAR5 cells. Cells were incubated with **15** at 0.5 or 2 µM or CYC065 at 0.5 µM for 48 h. DMSO diluent was employed as a control. Apoptosis was determined by annexin V/PI staining. Experiments were performed twice, and representative data are presented. Viable cells are shown in the lower left quadrant, and they were not stained by either staining reagent, whereas the early apoptotic cells in the lower right quadrant were stained by annexin V. The late apoptotic cells in the upper right quadrant were stained by both staining reagents, while the necrotic cells in the upper left quadrant were stained by PI. The sum of the percentages of cells undergoing early (annexin V positive/PI negative) and late apoptosis (annexin V positive/PI positive) is taken as the total percentage of the apoptotic cells.

To probe whether the antiproliferative activities of **15** were due to the induction of apoptosis, A2780 and OVCAR5 cells were incubated with **15** at concentrations of 0.5 and 2 µM for 48 h, and apoptosis was determined by annexin V and PI double staining. As Figure 5 shows, in A2780 cells, **15** induced 47% and 49% apoptosis at 0.5 and 2 µM, respectively, while only 1% was found in untreated cells. In contrast, CYC065 at 0.5 µM showed 73% apoptosis. In OVCAR5 cells, **15** caused 30% and 38% apoptosis at 0.5 and 2 µM, respectively, whereas 2% was observed in untreated cells. On the other hand, CYC065 induced 16% apoptosis. CYC065 induced more apoptosis in A2780 cells than did **15**, and the opposite held true in OVCAR5 cells. 

#### 2.6.3. Effect of **15** on Colony Formation 

**Figure 6:**
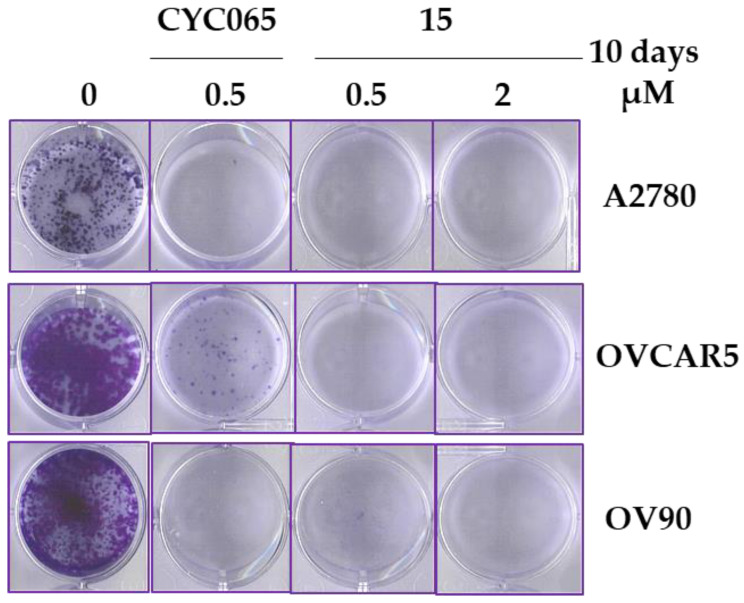
Effect of **15** on colony formation. Three different ovarian cancer cell lines were incubated with vehicle (DMSO), 0.5 or 2 µM **15**, or 0.5 µM CYC065 for 10 days. Colony formation was evaluated by crystal violet staining. Experiments were performed twice, and representative data are presented.

As Figure 6 illustrates, **15** inhibited the ability of three ovarian cancer cell lines (A2780, OVCAR5, and OV90) to form viable colonies at 0.5 and 2 µM. However, partial inhibition of colony formation was observed at 0.5 µM in OV90 cells. These results are in agreement with the observed antiproliferative effects of **15** (Table 1 and Table 4). Similarly, CYC065 reduced the clonogenic growth of all three cell lines, with the lowest effect on OVCAR5. 

#### 2.6.4. Western Blot Analysis 

**Figure 7:**
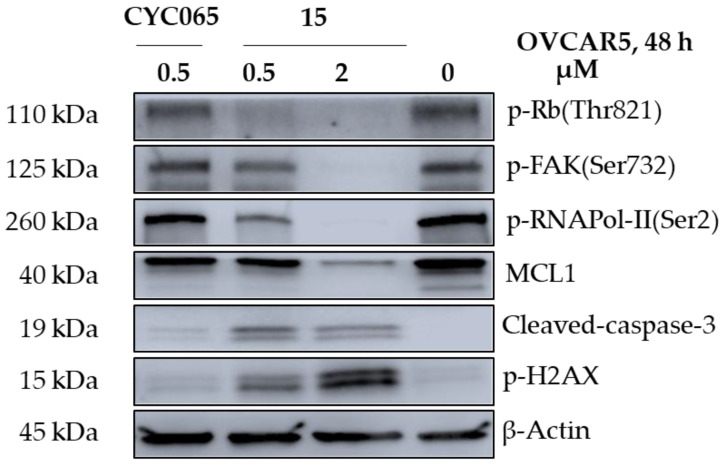
Western blot analysis of OVCAR5 cells treated with **15**. Cells were exposed to **15** at 0.5 or 2 µM or to CYC065 at 0.5 µM for 48 h. DMSO diluent was employed as a control. Experiments were performed twice, and representative data are presented.

To explore whether **15** could inhibit its target kinases within cancer cells and to understand the molecular mechanism underlying its anticancer effects, OVCAR5 cells were incubated with 0.5 or 2 µM of **15** or 0.5 µM of CYC065 for 48 h, and the phosphorylation levels of the physiological substrates of CDK2, CDK5, and CDK9 (i.e., Rb, FAK, and RNAPol-II, respectively) were determined by Western blotting. As shown in Figure 7, **15** completely abolished the kinase activity of CDK2, CDK5, and CDK9 at 2 µM as demonstrated by the disappearance of p-Rb(Thr821), p-FAK(Ser732), and p-RNAPol-II(Ser2), respectively. Additionally, **15** also caused double-strand DNA breaks, showcased by the appearance of phosphorylated histone H2AX (p-H2AX), which is an independent marker of DNA damage. The DNA damage resulted in apoptotic cell death which was evidenced by the cleavage of caspase-3 and the downregulation of the anti-apoptotic protein MCL1. 

## 3. Conclusions

The bioisosteric replacement of the phenylsulfonamide moiety of our previous lead compound **1** with pyrazole-derived groups led to the discovery of a new chemotype of CDK2 inhibitors, namely 4-(1-methyl-1*H*-pyrazol-4-yl)-*N*-(1*H*-pyrazol-4-yl)pyrimidin-2-amines. Among them, **14** and **15** inhibited CDK2 at single-digit nanomolar concentrations (*K*_i_ = 0.007 and 0.005 µM, respectively). The SAR analysis revealed that N-alkylation or topological change of the pyrazol-4-yl ring at the pyrimidinyl-C2-NH position had a detrimental effect on both the CDK2 inhibition and the antiproliferative activity. Similar effects were also observed upon derivatization of the other pyrazol-4-yl moiety at the pyrimidinyl-C4 position. Mechanistically, the antiproliferative activity of **15** was shown to be associated with inhibition of the phosphorylation of Rb, FAK, and RNAPol-II, cell cycle arrest at the S and G2/M phases, and induction of apoptosis. Thus, **15** can be considered a lead compound that could be further optimized into a potent and selective CDK2 inhibitor with potential anticancer activity. 

## 4. Experimental Section

### 4.1. Method for Computational Modeling 

Computational modeling was carried out using the crystal structure of CDK2/cyclin E in complex with a pyridopyrimidinone inhibitor (i.e., PF-06873600) (PDB ID: 7KJS). OMEGA was used for ligand preparation starting from the SMILES string, generating the lowest energy conformer of the ligand, which was retained for docking (OMEGA 4.2.1.2: OpenEye Scientific Software, Santa Fe, NM. http://www.eyesopen.com, accessed on 27 November 2022) [31]. Docking was undertaken using OEDocking and the chemgauss4 scoring function (OEDocking 4.1.2.1 OpenEye Scientific docking program, (http://www.eyesopen.com, accessed on 27 November 2022). The visualization was generated by Maestro Schrödinger Release 2021-1 as described elsewhere [32]. 

### 4.2. Chemistry

Chemicals and solvents were obtained from commercial sources and were used without further purification. A CEM Discover SP and Explorer 48/72/96 microwave system (CEM corporation, Matthews, NC, USA) controlled by the Synergy^TM^ software was used for microwave-assisted synthesis. The progression of a reaction was monitored by thin-layer chromatography using Merck 60 F_254_ silica gel-precoated aluminum plates and visualized by UV irradiation (254 nm). Flash column chromatography was carried out using a glass column packed with silica gel (230–400 mesh). An AB SCIEX TripleTOF^®^ 5600 mass spectrometer (Concord, Ontario, Canada) with ESI ionization mode was used to determine the high-resolution mass spectra. ^1^H and ^13^C NMR spectra were determined by a Bruker Avance III HD spectrometer (Faellanden, Switzerland) at 500.16 and 125.76 MHz, respectively, and were analyzed using the Bruker Topspin 4.1 program. Chemical shifts are reported in parts per million (ppm) and referenced to ^1^H signals of residual nondeuterated solvents and ^13^C signals of the deuterated solvents. The multiplicity of ^1^H NMR signals was reported as: s = singlet, d = doublet, and t = triplet. Coupling constant (*J*) values are reported in Hz. A Shimadzu Prominence UltraFast Liquid Chromatograph System (Kyoto, Japan) equipped with a CBM-20A communications bus module, a DGU-20A5R degassing unit, an LC-20AD liquid chromatograph pump, an SIL-20AHT auto-sampler, an SPD-M20A photodiode array detector, a CTO-20A column oven, and a Phenomenex Kinetex 5u C18 100A 250 mm × 4.60 mm column was used to determine the purity of the final compounds. All the final compounds used for biological assays had a purity greater than 95%. Method A (gradient 5%–95% methanol containing 0.1% formic acid (FA) over 20 min at a flow rate of 1 mL/min, followed by 95% methanol containing 0.1% FA over 5 min) and method B (gradient 5%–95% MeCN containing 0.1% FA over 20 min at a flow rate of 1 mL/min, followed by 95% MeCN containing 0.1% FA over 5 min) were used for analytic HPLC. The melting points (m.p.) of final compounds were determined using a Stuart SMP10 melting point apparatus and are uncorrected.

#### 4.2.1. General Synthetic Procedure A: Suzuki Coupling [33]

A mixture of a pyrazole boronic acid pinacol ester (1.30 equivalent (eqv.)), a 5-substituted-2,4-dichloropyrimidine (1.00 eqv.), PdCl_2_(dppf)·DCM (0.10 eqv.), and potassium carbonate (2.50 eqv.) in 1,4-dioxane:ethanol:water (7:3:4, *v/v/v*) was degassed, purged with nitrogen (3×), and heated under nitrogen at 80 °C for 12 h. The reaction mixture was cooled down and concentrated under reduced pressure, and the residue was partitioned between water and ethyl acetate (1:2, *v/v*). The aqueous layer was extracted with ethyl acetate (3×). The organic layer and extracts were combined, washed with brine, dried over Na_2_SO_4_, and filtered; the filtrate was concentrated under reduced pressure. The residue was purified by flash column chromatography (silica gel) eluting with 20–40% ethyl acetate in petroleum ether (unless otherwise stated) to afford the desired product. 

#### 4.2.2. General Synthetic Procedure B: Buchwald–Hartwig Amination [33]

A mixture of a 2-chloropyrimidine (1.20 eqv.), an arylamine (1.00 eqv.), Cs_2_CO_3_ (2.50 eqv.), Pd_2_(dba)_3_ (0.05 eqv.), and Xantphos (0.10 eqv.) in 1,4-dioxane was degassed, purged with nitrogen (3×), and subjected to microwave irradiation at 140 °C for 1 h. The reaction mixture was concentrated under reduced pressure, and the residue was partitioned between water and ethyl acetate (1:2, *v/v*). The aqueous layer was extracted with ethyl acetate (3×), and the organic layer and extracts were combined, washed with brine, dried over Na_2_SO_4_, and filtered. The filtrate was concentrated under reduced pressure, and the residue was purified by flash column chromatography (silica gel) to give the desired product. ^1^H & ^13^C NMR spectra, HRMS & HPLC chromatograms of compounds see Supplementary Materials.

2-Chloro-5-fluoro-4-(1-methyl-1*H*-pyrazol-4-yl)pyrimidine (**6**): Prepared following the general synthetic procedure A using 2,4-dichloro-5-fluoropyrimidine (**4**) (5.00 g, 30.0 mmol) and (1-methyl-1*H*-pyrazol-4-yl)boronic acid pinacol ester (**2**) (5.20 g, 25.0 mmol). White solid (3.70 g, 69%). ^1^H NMR (DMSO-*d*_6_): *δ* 3.96 (s, 3H, CH_3_); 8.11 (s, 1H, pyrazolyl-H); 8.57 (s, 1H, pyrazolyl-H); and 8.79 (d, 1H, *J* = 2.4, pyrimidinyl-H). HRMS (ESI-TOF) 213.0345 [M(^35^Cl)+H]^+^ and 215.0316 [M(^37^Cl)+H]^+^; calcd. for C_8_H_7_ClFN_4_^+^ 213.0338 [M(^35^Cl)+H]^+^ and 215.0309 [M(^37^Cl)+H]^+^.

2,5-Dichloro-4-(1-methyl-1*H*-pyrazol-4-yl)pyrimidine (**7**): Prepared following the general synthetic procedure A using 2,4,5-trichloropyrimidine (**5**) (5.50 g, 30.0 mmol) and (1-methyl-1*H*-pyrazol-4-yl)boronic acid pinacol ester (**2**) (5.20 g, 25.0 mmol). White solid (3.70 g, 65%). ^1^H NMR (CDCl_3_): *δ* 4.02 (s, 3H, CH_3_); 8.37 (s, 1H, pyrazolyl-H); 8.39 (s, 1H, pyrazolyl-H); and 8.52 (s, 1H, pyrimidinyl-H). HRMS (ESI-TOF) 229.0051 [M(2 × ^35^Cl)+H]^+^, 231.0018 [M(^35^Cl and ^37^Cl)+H]^+^ and 232.9987 [M(2 × ^37^Cl)+H]^+^, calcd. For C_8_H_7_Cl_2_N_4_^+^ 229.0043 [M(2 × ^35^Cl)+H]^+^, 231.0013 [M(^35^Cl and ^37^Cl)+H]^+^, and 232.9984 [M(2 × ^37^Cl)+H]^+^.

2-Chloro-4-(1,3-dimethyl-1*H*-pyrazol-4-yl)-5-fluoropyrimidine (**8**): Prepared following the general synthetic procedure A using (1,3-dimethyl-1*H*-pyrazol-4-yl)boronic acid pinacol ester (**3**) (5.60 g, 25.0 mmol) and 2,4-dichloro-5-fluoropyrimidine (**4**) (5.00 g, 30.0 mmol). Off-white solid (3.60 g, 63%). ^1^H NMR (DMSO-*d*_6_): *δ* 2.54 (s, 3H, CH_3_); 3.84 (s, 3H, CH_3_); 7.93 (s, 1H, pyrazolyl-H); 8.27 (d, 1H, *J* = 2.7, pyrimidinyl-H). HRMS (ESI-TOF) 227.0502 [M(^35^Cl)+H]^+^ and 229.0470 [M(^37^Cl)+H]^+^; calcd. For C_9_H_9_ClFN_4_^+^ 227.0495 [M(^35^Cl)+H]^+^, and 229.0465 [M(^37^Cl)+H]^+^.

2,5-Dichloro-4-(1,3-dimethyl-1*H*-pyrazol-4-yl)pyrimidine (**9**): Prepared following the general synthetic procedure A using 2,4,5-trichloropyrimidine (**5**) (5.50 g, 30.0 mmol) and (1,3-dimethyl-1*H*-pyrazol-4-yl)boronic acid pinacol ester (**3**) (5.60 g, 25.0 mmol). Off-white solid (3.60 g, 59%). ^1^H NMR (DMSO-*d*_6_): *δ* 2.42 (s, 3H, CH_3_); 3.87 (s, 3H, CH_3_); 8.63 (s, 1H, pyrazolyl-H); 8.76 (s, 1H, pyrimidinyl-H). HRMS (ESI-TOF) 243.0213 [M(2 × ^35^Cl)+H]^+^, 245.0180 [M(^35^Cl and ^37^Cl)+H]^+^ and 247.0151 [M(2 × ^37^Cl)+H]^+^; calcd. For C_9_H_9_ClFN_4_^+^ 243.0199 [M(2 × ^35^Cl)+H]^+^, 245.0170 [M(^35^Cl and ^37^Cl)+H]^+^, and 247.0140 [M(2 × ^37^Cl)+H]^+^.

2-(4-Nitro-1*H*-pyrazol-1-yl)acetamide (**12**): To a solution of 4-nitro-1*H*-pyrazole (**10**) (1.70 g, 15.0 mmol) in acetonitrile (150 mL) were added K_2_CO_3_ (4.10 g, 30.0 mmol) and 2-bromoacetamide (**11**) (2.10 g, 15.0 mmol), and the mixture was heated at 60 °C overnight, cooled down, filtered, and washed with acetonitrile (50 mL). The filtrate and washing were concentrated under reduced pressure, and the residue was triturated with diethyl ether:petroleum ether (2:1) to yield **12** as a white solid (2.40 g, 95%). ^1^H NMR (DMSO-*d*_6_): *δ* 4.88 (s, 2H, CH_2_), 7.41 (s, 1H, acetamide-NH), 7.68 (s, 1H, acetamide-NH), 8.27 (s, 1H, pyrazolyl-H), 8.83 (s, 1H, pyrazolyl-H). HRMS (ESI-TOF) 171.0523 [M+H]^+^; calcd. for C_5_H_7_N_4_O_3_^+^ 171.0513 [M+H]^+^.

2-(4-Amino-1*H*-pyrazol-1-yl)acetamide (**13c**): To a suspension of 2-(4-nitro-1*H*-pyrazol-1-yl)acetamide (**12**) (2.40 g, 14.1 mmol) in methanol (100 mL) was added 10% palladium on carbon (150 mg, 141 µmol), and the reaction mixture was stirred at room temperature under hydrogen overnight. The reaction product was filtered through a pad of Celite, and the residue was washed with methanol (100 mL). The filtrate and washing were combined and concentrated under reduced pressure to afford **13c** as a dark brown solid, which was used in the next step without further purification (1.80 g, 90%). ^1^H NMR (DMSO-*d*_6_): *δ* 3.81 (s, 2H, CH_2_); 4.51 (s, 2H, NH_2_); 6.89 (s, 1H, pyrazolyl-H); 6.99 (s, 1H, pyrazolyl-H); and 7.14 (s, 2H, acetamide-NH_2_). HRMS (ESI-TOF) 141.0778 [M+H]^+^; calcd. for C_5_H_9_N_4_O^+^ 141.0771 [M+H]^+^.

5-Fluoro-4-(1-methyl-1*H*-pyrazol-4-yl)-*N*-(1*H*-pyrazol-4-yl)pyrimidin-2-amine (**14**): Prepared from 2-chloro-5-fluoro-4-(1-methyl-1*H*-pyrazol-4-yl)pyrimidine (**6**) (213 mg, 1.00 mmol) and 4-amino-1*H*-pyrazole (**13a**) (100 mg, 1.20 mmol) following the general synthetic procedure B. The residue was purified by flash column chromatography, eluting with 0–5% methanol in ethyl acetate to afford 14 as a yellow solid (34 mg, 13%). m.p. 253.8–254.8 °C. ^1^H NMR (DMSO-*d*_6_): *δ* 3.94 (s, 3H, CH_3_); 7.71 (s, 2H, pyrazolyl-H); 8.04 (s, 1H, pyrazolyl-H), 8.38 (s, 1H, pyrazolyl-H), 8.41 (d, 1H, *J* = 2.9, pyrimidinyl-H), 9.38 (s, 1H, NH); and 12.43 (s, 1H, pyrazolyl-NH). ^13^C NMR (DMSO-*d*_6_): *δ* 39.4, 116.2 (d, *J*_C–F_ = 5.5), 117.9, 123.5, 130.3, 132.8 (d, *J*_C–F_ = 8.0), 139.1 (d, *J*_C–F_ = 5.2), 146.4, 148.3 (d, *J*_C–F_ = 246.0), and 156.8 (one carbon signal overlapping or obscured). HRMS (ESI-TOF): 260.1064 [M+H]^+^; calcd. for C_11_H_11_FN_7_^+^ 260.1054 [M+H]^+^. Anal. RP/HPLC Method A: *t*_R_ 15.53 min, purity 100%; Method B: *t*_R_ 11.35 min, purity > 99%.

5-Chloro-4-(1-methyl-1*H*-pyrazol-4-yl)-*N*-(1*H*-pyrazol-4-yl)pyrimidin-2-amine (**15**): Prepared from 2,5-dichloro-4-(1-methyl-1*H*-pyrazol-4-yl)pyrimidine (**7**) (229 mg, 1.00 mmol) and 4-amino-1*H*-pyrazole (**13a**) (100 mg, 1.20 mmol) following the general synthetic procedure B. The residue was purified by flash column chromatography, eluting with 0–5% methanol in ethyl acetate to afford **15** as a yellow solid (52 mg, 19%). m.p. 269.1 °C. ^1^H NMR (DMSO-*d*_6_): *δ* 3.97 (s, 3H, CH_3_); 7.63 (s, 1H, pyrazolyl-H); 7.92 (s, 1H, pyrazolyl-H); 8.19 (s, 1H, pyrazolyl-H); 8.43 (s, 1H, pyrazolyl-H); 8.59 (s, 1H, pyrimidinyl-H); 9.56 (s, 1H, NH); and 12.51 (s, 1H, pyrazolyl-NH). ^13^C NMR (DMSO-*d*_6_): *δ* 40.5, 114.2, 118.8, 118.9, 122.9, 130.9, 133.3, 140.1, 155.4, 158.1, and 158.7. HRMS (ESI-TOF) 276.0758 [M(^35^Cl)+H]^+^ and 278.0728 [M(^37^Cl)+H]^+^; calcd. for C_11_H_11_ClN_7_^+^ 276.0759 [M(^35^Cl)+H]^+^ and 278.0730 [M(^37^Cl)+H]^+^. Anal. RP/HPLC Method A: *t*_R_ 16.84 min, purity ˃ 95%; Method B: *t*_R_ 12.34 min, purity ˃ 95%.

5-Fluoro-*N*,4-bis(1-methyl-1*H*-pyrazol-4-yl)pyrimidin-2-amine (**16**): Prepared from 2-chloro-5-fluoro-4-(1-methyl-1*H*-pyrazol-4-yl)pyrimidine (**6**) (213 mg, 1.00 mmol) and 1-methyl-1*H*-pyrazole-4-amine (**13b**) (117 mg, 1.20 mmol) following the general synthetic procedure B. The residue was purified by flash column chromatography, eluting with 0–2.5% methanol in ethyl acetate to give **16** as a white solid (74 mg, 27%). m.p. 202.8–203.7 °C. ^1^H NMR (DMSO-*d*_6_): *δ* 3.84 (s, 3H, CH_3_); 3.97 (s, 3H, CH_3_); 7.49 (s, 1H, pyrazolyl-H); 7.91 (s, 1H, pyrazolyl-H); 8.09 (s, 1H, pyrazolyl-H); 8.41 (s, 1H, pyrazolyl-H); 8.43 (d, 1H, *J* = 3.1, pyrimidinyl-H); and 9.42 (s, 1H, NH). ^13^C NMR (DMSO-*d*_6_): *δ* 39.2, 39.4, 116.2 (d, *J*_C–F_ = 5.4), 120.4, 123.9, 130.0, 132.8 (d, *J*_C–F_ = 8.3), 139.3 (d, *J*_C–F_ = 4.8), 146.3, 148.4 (d, *J*_C–F_ = 246.0), and 156.6 (one carbon signal overlapping or obscured). HRMS (ESI-TOF) 274.1210 [M+H]^+^; calcd. for C_12_H_13_FN_7_^+^ 274.1211 [M+H]^+^. Anal. RP/HPLC Method A: *t*_R_ 16.63 min, purity 100%; Method B: *t*_R_ 12.55 min, purity 100%.

5-Chloro-*N*,4-bis(1-methyl-1*H*-pyrazol-4-yl)pyrimidin-2-amine (**17**): Prepared from 2,5-dichloro-4-(1-methyl-1*H*-pyrazol-4-yl)pyrimidine (**7**) (229 mg, 1.00 mmol) and 1-methyl-1*H*-pyrazole-4-amine (**13b**) (117 mg, 1.20 mmol) following the general synthetic procedure B. The residue was purified by flash column chromatography, eluting with 0–2.5% methanol in ethyl acetate to give **17** as a white solid (67 mg, 23%). m.p. 201.2–202.3 °C. ^1^H NMR (DMSO-*d*_6_): *δ* 3.84 (s, 3H, CH_3_); 3.97 (s, 3H, CH_3_); 7.53 (s, 1H, pyrazolyl-H); 7.87 (s, 1H, pyrazolyl-H); 8.21 (s, 1H, pyrazolyl-H); 8.40 (s, 1H, pyrazolyl-H); 8.55 (s, 1H, pyrimidinyl-H); and 9.42 (s, 1H, NH). ^13^C NMR (DMSO-*d*_6_): *δ* 40.6, 40.7, 114.4, 118.9, 121.0, 123.4, 130.5, 133.2, 140.1, 155.5, 158.1, and 158.5. HRMS (ESI-TOF): 290.0918 [M(^35^Cl)+H]^+^ and 292.0893 [M(^37^Cl)+H]^+^; calcd. for C_12_H_13_ClN_7_^+^ 290.0916 [M(^35^Cl)+H]^+^ and 292.0886 [M(^37^Cl)+H]^+^. Anal. RP/HPLC Method A: *t*_R_ 11.27 min, purity 100%; Method B: *t*_R_ 13.52 min, purity 100%.

2-(4-((5-Fluoro-4-(1-methyl-1*H*-pyrazol-4-yl)pyrimidin-2-yl)amino)-1*H*-pyrazol-1-yl)acetamide (**18**): Prepared from 2-chloro-5-fluoro-4-(1-methyl-1*H*-pyrazol-4-yl)pyrimidine (**6**) (213 mg, 1.00 mmol) and 2-(4-amino-1*H*-pyrazol-1-yl)acetamide (**13c**) (140 mg, 1.00 mmol) following the general synthetic procedure B. The residue was purified by flash column chromatography, eluting with 0–4% methanol in ethyl acetate to give **18** as an off-white solid (54 mg, 17%). m.p. 283.1–284.1 °C. ^1^H NMR (DMSO-*d*_6_): *δ* 4.02 (s, 3H, CH_3_); 4.82 (s, 2H, CH_2_); 7.29 (s, 1H, pyrazolylacetamide-NH); 7.45 (s, 1H, pyrazolylacetamide-NH); 7.58 (s, 1H, pyrazolyl-H); 8.05 (s, 1H, pyrazolyl-H); 8.17 (s, 1H, pyrazolyl-H), 8.47 (s, 1H, pyrazolyl-H); 8.50 (d, 1H, *J* = 2.4, pyrimidinyl-H); and 9.52 (s, 1H, NH). ^13^C NMR (DMSO-*d*_6_): *δ* 39.4, 54.5, 116.2, 121.2, 123.9, 130.7, 132.9 (d, *J*_C–F_ = 8.3), 139.3, 146.5, 148.4 (d, *J*_C–F_ = 246.3), 156.6, and 169.5 (one carbon signal overlapping or obscured). HRMS (ESI-TOF) 317.1273 [M + H]^+^; calcd. for C_13_H_14_FN_8_O^+^ 317.1270 [M + H]^+^. Anal. RP/HPLC: Method A, *t*_R_ 14.43 min, purity 100%; Method B, *t*_R_ 8.14 min, purity ˃ 99%. 

2-(4-((5-Chloro-4-(1-methyl-1*H*-pyrazol-4-yl)pyrimidin-2-yl)amino)-1*H*-pyrazol-1-yl)acetamide (**19**): Prepared from 2,5-dichloro-4-(1-methyl-1*H*-pyrazol-4-yl)pyrimidine (**7**) (229 mg, 1.00 mmol) and 2-(4-amino-1*H*-pyrazol-1-yl)acetamide (**13c**) (168 mg, 1.20 mmol) following the general synthetic procedure B. The residue was purified by flash column chromatography, eluting with 0–5% methanol in ethyl acetate to yield **19** as a pale orange solid (63 mg, 19%). m.p. 267.6–268.1 °C. ^1^H NMR (DMSO-*d*_6_): *δ* 3.96 (s, 3H, CH_3_); 4.77 (s, 2H, CH_2_); 7.25 (s, 1H, pyrazolylacetamide-NH); 7.44 (s, 1H, pyrazolylacetamide-NH); 7.54 (s, 1H, pyrazolyl-H); 7.97 (s, 1H, pyrazolyl-H); 8.24 (s, 1H, pyrazolyl-H); 8.43 (s, 1H, pyrazolyl-H); 8.61 (s, 1H, pyrimidinyl-H); and 9.61 (s, 1H, NH). ^13^C NMR (DMSO-*d*_6_): *δ* 39.4, 54.5, 114.3, 118.9, 121.7, 123.4, 130.9, 133.4, 140.2, 157.9, 158.6, and 169.4 (one carbon signal overlapping or obscured). HRMS (ESI-TOF) 333.0975 [M(^35^Cl)+H]^+^ and 335.0944 [M(^37^Cl)+H]^+^; calcd. for C_13_H_14_ClN_8_O^+^ 333.0974 [M(^35^Cl)+H]^+^ and 335.0945 [M(^37^Cl)+H]^+^. Anal. RP/HPLC Method A: *t*_R_ 15.55 min, purity ˃ 98%; Method B: *t*_R_ 11.46 min, purity ˃ 98%.

4-(1,3-Dimethyl-1*H*-pyrazol-4-yl)-5-fluoro-*N*-(1-methyl-1*H*-pyrazol-4-yl)pyrimidin-2-amine (**20**): Prepared from 2-chloro-4-(1,3-dimethyl-1*H*-pyrazol-4-yl)-5-fluoropyrimidine (**8**) (226 mg, 1.00 mmol) and 1-methyl-1*H*-pyrazole-4-amine (**13b**) (117 mg, 1.20 mmol) following the general synthetic procedure B. The residue was purified by flash column chromatography eluting, with 0–2.5% methanol in ethyl acetate to yield **20** as a pale orange solid (66 mg, 23%). m.p. 189.1–190.1 °C. ^1^H NMR (DMSO-*d*_6_): *δ* 2.49 (s, 3H, CH_3_); 3.79 (s, 3H, CH_3_); 3.84 (s, 3H, CH_3_); 7.46 (s, 1H, pyrazolyl-H); 7.83 (s, 1H, pyrazolyl-H); 8.22 (d, 1H, *J* = 3.7, pyrazolyl-H); 8.38 (d, 1H, *J* = 3.3, pyrimidinyl-H); and 9.21 (s, 1H, NH). ^13^C NMR (DMSO-*d*_6_): *δ* 15.0, 39.5, 111.9, 121.2, 123.8, 130.7, 134.3 (d, *J*_C–F_ = 13.5), 145.3, 148.3, 148.7 (d, *J*_C-F_ = 246.0), 148.8, and 156.7 (one carbon signal overlapping or obscured). HRMS (ESI-TOF) 288.1378 [M+H]^+^; calcd. for C_13_H_15_FN_7_^+^ 288.1367. Anal. RP/HPLC Method A: *t*_R_ 17.31 min, purity ˃ 99%; Method B: *t*_R_ 13.10 min, purity ˃ 99%.

5-Chloro-4-(1,3-dimethyl-1*H*-pyrazol-4-yl)-*N*-(1-methyl-1*H*-pyrazol-4-yl)pyrimidin-2-amine (**21**): Prepared from 2,5-dichloro-4-(1,3-dimethyl-1*H*-pyrazol-4-yl)pyrimidine (**9**) (243 mg, 1.00 mmol) and 1-methyl-1*H*-pyrazole-4-amine (**13b**) (117 mg, 1.20 mmol) following the general synthetic procedure B. The residue was purified by flash column chromatography eluting, with 75% ethyl acetate in petroleum ether and ramping up to 2.5% methanol in ethyl acetate to afford **21** as an orange solid (67 mg, 22%). m.p. 182.3–183.3 °C. ^1^H NMR (DMSO-*d*_6_): *δ* 2.41 (s, 3H, CH_3_); 3.80 (s, 3H, CH_3_); 3.85 (s, 3H, CH_3_); 7.48 (s, 1H, pyrazolyl-H); 7.83 (s, 1H, pyrazolyl-H); 8.37 (s, 1H, pyrazolyl-H); 8.41 (s, 1H, pyrimidinyl-H); and 9.44 (s, 1H, NH). ^13^C NMR (DMSO-*d*_6_): *δ* 14.5, 38.9, 39.5, 114.7, 115.5, 121.4, 123.3, 130.8, 134.0, 148.3, 157.5, 157.9, and 158.1. HRMS (ESI-TOF) 304.1080 [M(^35^Cl)+H]^+^ and 306.1051 [M(^37^Cl)+H]^+^; calcd. For C_13_H_15_ClN_7_^+^ 304.1072 [M(^35^Cl)+H]^+^ and 306.1043 [M(^37^Cl)+H]^+^. Anal. RP/HPLC Method A: *t*_R_ 18.22 min, purity 100%; Method B: *t*_R_ 13.79 min, purity ˃ 99%.

5-Chloro-4-(1-methyl-1*H*-pyrazol-4-yl)-*N*-(1*H*-pyrazol-5-yl)pyrimidin-2-amine (**23**): Prepared from 2,5-dichloro-4-(1-methyl-1*H*-pyrazol-4-yl)pyrimidine (**7**) (229 mg, 1.00 mmol) and 1*H*-pyrazole-3-amine (**22**) (100 mg, 1.20 mmol) following the general synthetic procedure B. The residue was purified by flash column chromatography eluting, with 75–100% ethyl acetate in petroleum ether to afford **23** as a white solid (77 mg, 28%). m.p. 245.4–246.4 °C. ^1^H NMR (DMSO-*d*_6_): *δ* 3.96 (s, 3H, CH_3_); 6.63 (s, 1H, pyrazolyl-H); 7.62 (s, 1H, pyrazolyl-H); 8.19 (s, 1H, pyrazolyl-H); 8.44 (s, 1H, pyrazolyl-H); 8.58 (s, 1H, pyrimidinyl-H); 9.86 (s, 1H, NH); and 12.25 (s, 1H, pyrazolyl-NH). ^13^C NMR (DMSO-*d*_6_): *δ* 39.4, 96.6, 115.0, 118.9, 128.8, 133.3, 140.1, 155.3, 158.2, and 158.6 (one carbon signal overlapping or obscured). HRMS (ESI-TOF) 276.0758 [M(^35^Cl)+H]^+^ and 278.0728 [M(^37^Cl)+H]^+^; calcd. For C_11_H_11_ClN_7_^+^ 276.0759 [M(^35^Cl)+H]^+^ and 278.0730 [M(^37^Cl)+H]^+^. Anal. RP/HPLC Method A: *t*_R_ 17.48 min, purity 100%, Method B: *t*_R_ 12.57 min, purity > 98%.

*N*-(5-Chloro-4-(1-methyl-1*H*-pyrazol-4-yl)pyrimidin-2-yl)thiazol-2-amine (**25**): Prepared from 2,5-dichloro-4-(1-methyl-1*H*-pyrazol-4-yl)pyrimidine (**7**) (229 mg, 1.00 mmol) and thiazole-2-amine (**24**) (120 mg, 1.20 mmol) following the general synthetic procedure B. The residue was purified by flash column chromatography eluting with 75–100% ethyl acetate in petroleum ether to give **25** as an off-white solid (79 mg, 27%). m.p. 243.6–244.5 °C. ^1^H NMR (DMSO-*d*_6_): *δ* 4.00 (s, 3H, CH_3_); 7.18 (d, 1H, *J* = 3.5, thiazolyl-H); 7.47 (d, 1H, *J* = 3.5, thiazolyl-H); 8.42 (s, 1H, pyrazolyl-H); 8.65 (s, 1H, pyrazolyl-H); 8.72 (s, 1H, pyrimidinyl-H); and 11.86 (s, 1H, NH). ^13^C NMR (DMSO-*d*_6_): *δ* 39.4, 112.9, 116.8, 118.4, 133.8, 138.4, 140.7, 155.1, 155.5, 159.1, and 160.0. HRMS (ESI-TOF) 293.0380 [M(^35^Cl)+H]^+^ and 295.0349 [M(^37^Cl)+H]^+^; calcd. For C_11_H_10_ClN_6_S^+^ 293.0371 [M(^35^Cl)+H]^+^ and 295.0342 [M(^37^Cl)+H]^+^. Anal. RP/HPLC Method A: *t*_R_ 19.54 min, purity 100%, method B: *t*_R_ 14.52 min, purity ˃ 99%.

4-(2-(4-(4,4,5,5-Tetramethyl-1,3,2-dioxaborolan-2-yl)-1*H*-pyrazol-1-yl)ethyl)morpholine (**28**): To a solution of 1*H*-pyrazole-4-boronic acid pinacol ester (**26**) (4.90 g, 25.0 mmol) in acetonitrile (20 mL) were added caesium carbonate (28.5 g, 87.5 mmol) and 4-(2-chloroethyl)morpholine (**27**) (5.60 g, 37.5 mmol), and the reaction mixture was heated at reflux for 24 h, cooled down, and partitioned between water (100 mL) and ethyl acetate (100 mL). The organic layer was separated, and the aqueous layer was extracted with ethyl acetate (2 × 100 mL). The organic layer and washings were combined, washed with brine (100 mL), and concentrated under reduced pressure to afford **28** as a brown solid that was used in the next step without further purification and full characterization (4.40 g, 57%). HRMS (ESI-TOF) 308.2138 [M+H]^+^; calcd. For C_15_H_27_BN_3_O_3_^+^ 308.2140 [M+H]^+^. 

4-(2-(4-(2-Chloro-5-fluoropyrimidin-4-yl)-1*H*-pyrazol-1-yl)ethyl)morpholine (**29**): Prepared from 4-(2-(4-(4,4,5,5-tetramethyl-1,3,2-dioxaborolan-2-yl)-1*H*-pyrazol-1-yl)ethyl)morpholine (**28**) (4.40 g, 14.0 mmol) and 2,4-dichloro-5-fluoropyrimidine (**4**) (1.60 g, 9.30 mmol) following the general synthetic procedure A. The residue was purified by flash column chromatography, eluting with 0–4% methanol in dichloromethane to yield **29** as a violet gummy solid (1.30 g, 45%). ^1^H NMR (DMSO-*d*_6_): *δ* 2.43 (t, 4H, *J* = 4.5, morpholinyl-H); 2.75 (t, 2H, *J* = 6.4, ethyl-CH_2_); 3.54 (t, 4H, *J* = 4.5, morpholinyl-H); 4.37 (t, 2H, *J* = 6.4, ethyl-CH_2_); 6.99 (s, 1H, pyrazolyl-H); and 7.35 (s, 1H, pyrazolyl-H), 8.63 (d, 1H, *J* = 2.8, pyrimidinyl-H). HRMS (ESI-TOF) 312.1024 [M(^35^Cl)+H]^+^ and 314.0995 [M(^37^Cl)+H]^+^; calcd. For C_13_H_16_ClFN_5_O^+^ 312.1022 [M(^35^Cl)+H]^+^ and 314.0993 [M(^37^Cl)+H]^+^.

4-(2-(4-(2,5-Dichloropyrimidin-4-yl)-1*H*-pyrazol-1-yl)ethyl)morpholine (**30**): Prepared from 4-(2-(4-(4,4,5,5-tetramethyl-1,3,2-dioxaborolan-2-yl)-1*H*-pyrazol-1-yl)ethyl)morpholine (**28**) (4.40 g, 14.0 mmol) and 2,4,5-trichloropyrimidine (**5**) (1.70 g, 9.30 mmol) following the general synthetic procedure A. The residue was purified by flash column chromatography eluting, with 0–2.5% methanol in dichloromethane to give **30** as a violet gummy solid (1.20 g, 40%). ^1^H NMR (DMSO-*d_6_*): *δ* 2.44 (t, 4H, *J* = 4.3, morpholinyl-H); 2.76 (t, 2H, *J* = 6.3, ethyl-CH_2_); 3.55 (t, 4H, *J* = 4.3, morpholinyl-H); 4.38 (t, 2H, *J* = 6.3, ethyl-CH_2_); 7.01 (s, 1H, pyrazolyl-H); 8.40 (s, 1H, pyrazolyl-H); and 8.73 (s, 1H, pyrimidinyl-H). HRMS (ESI-TOF) 328.0729 [M(2 × ^35^Cl)+H]^+^, 330.0700 [M(^35^Cl and ^37^Cl)+H]^+^ and 332.0670 [M(2 × ^37^Cl)+H]^+^; calcd. For C_20_H_25_ClN_7_O_3_S^+^ 328.0727 [M(2 × ^35^Cl)+H]^+^, 330.0697 [M(^35^Cl and ^37^Cl)+H]^+^, and 332.0668 [M(2 × ^37^Cl)+H]^+^.

5-Fluoro-*N*-(1-methyl-1*H*-pyrazol-4-yl)-4-(1-(2-morpholinoethyl)-1*H*-pyrazol-4-yl)pyrimidin-2-amine (**31**): Prepared from 4-(2-(4-(2-Chloro-5-fluoropyrimidin-4-yl)-1*H*-pyrazol-1-yl)ethyl)morpholine (**29**) (312 mg, 1.00 mmol) and 1-methyl-1*H*-pyrazole-4-amine (**13b**) (117 mg, 1.20 mmol) following the general synthetic procedure B. The residue was purified by flash column chromatography, eluting with 0–7.5% methanol in ethyl acetate to afford **31** as an orange solid (63 mg, 17%). **m.p.** 198.3–199.3 °C. ^1^H NMR (DMSO-*d*_6_): *δ* 2.44 (t, 4H, *J* = 4.5, morpholinyl-H); 2.76 (t, 2H, *J* = 6.4, ethylCH_2_); 3.55 (t, 4H, *J* = 4.5, morpholinyl-H); 3.83 (s, 3H, CH_3_); 4.36 (t, 2H, *J* = 6.4, ethylCH_2_); 7.50 (s, 1H, pyrazolyl-H), 7.90 (s, 1H, pyrazolyl-H); 8.11 (s, 1H, pyrazolyl-H); 8.43 (d, 1H, *J* = 3.0, pyrazolyl-H); 8.45 (s, 1H, NH); and 9.42 (s, 1H, pyrimidinyl-H). ^13^C NMR (DMSO-*d*_6_): *δ* 39.2, 49.2, 53.5, 57.9, 66.7, 115.9, 120.4, 124.0, 130.0, 132.7 (d, *J*_C–F_ = 8.4), 139.2, 146.5, 148.4 (d, *J*_C–F_ = 246.4), and 156.6 (three carbon signals overlapping or obscured). HRMS (ESI-TOF) 373.1894 [M + H]^+^; calcd. for C_17_H_22_FN_8_O^+^ 373.1895 [M + H]^+^. Anal. RP/HPLC Method A: *t*_R_ 12.12 min, purity 100%; Method B *t*_R_ 9.31 min, purity ˃ 99%.

5-Chloro-*N*-(1-methyl-1*H*-pyrazol-4-yl)-4-(1-(2-morpholinoethyl)-1*H*-pyrazol-4-yl)pyrimidin-2-amine (**32**): Prepared from 4-(2-(4-(2,5-dichloropyrimidin-4-yl)-1*H*-pyrazol-1-yl)ethyl)morpholine (**30**) (328 mg, 1.00 mmol) and 1-methyl-1*H*-pyrazole-4-amine (**13b**) (117 mg, 1.20 mmol) following the general synthetic procedure B. The residue was purified by flash column chromatography, eluting with 0–7.5% methanol in ethyl acetate to afford **32** as a pale violet solid (62 mg, 16%). m.p. 128.5–129.5 °C. ^1^H NMR (DMSO-*d*_6_): *δ* 2.42 (t, 4H, *J* = 4.5, morpholinyl-H); 2.74 (t, 2H, *J* = 6.4, ethyl-CH_2_); 3.54 (t, 4H, *J* = 4.5, morpholinyl-H); 3.82 (s, 3H, CH_3_); 4.35 (t, 2H, *J* = 6.4, ethyl-CH_2_); 7.51 (s, 1H, pyrazolyl-H); 7.87 (s, 1H, pyrazolyl-H); 8.22 (s, 1H, pyrazolyl-H); 8.41 (s, 1H, pyrazolyl-H); 8.64 (s, 1H, pyrimidinyl-H); and 9.56 (s, 1H, NH). ^13^C NMR (DMSO-*d*_6_): *δ* 39.2, 49.3, 53.6, 57.9, 66.7, 114.3, 116.2, 118.6, 120.9, 123.4, 127.6, 130.3, 133.2, 140.1, 141.9, 157.9, and 158.6. HRMS (ESI-TOF) 389.1595 [M(^35^Cl)+H]^+^ and 391.1566 [M(^37^Cl)+H]^+^; calcd. for C_17_H_22_ClN_8_O^+^ 389.1600 [M(^35^Cl)+H]^+^ and 391.1571 [M(^37^Cl)+H]^+^. Anal. RP/HPLC Method A: *t*_R_ 13.47 min, purity ˃ 99%; Method B: *t*_R_ 9.89 min, purity ˃ 98%.

2-Chloro-5-fluoro-4-(1-methyl-1*H*-pyrazol-5-yl)pyrimidine (**34**): Prepared from 2,4-dichloro-5-fluoropyrimidine (**4**) (2.50 g, 15.0 mmol) and 1-methyl-1*H*-pyrazole-5-boronic acid pinacol ester (**33**) (2.10 g, 10.0 mmol) following the general synthetic procedure A. The residue was purified by flash column chromatography, eluting with 20–40% ethyl acetate in petroleum ether to afford **34** as a white solid (893 mg, 42%). ^1^H NMR (DMSO-*d*_6_): *δ* 4.32 (s, 3H, CH_3_); 7.00 (t, 1H, *J* = 2.2, pyrazolyl-H); 7.58 (s, 1H, pyrazolyl-H); and 8.52 (s, 1H, pyrimidinyl-H). HRMS (ESI-TOF) 213.0335 [M(^35^Cl)+H]^+^ and 215.0316 [M(^37^Cl)+H]^+^; calcd. for C_8_H_7_ClFN_4_^+^ 213.0338 [M(^35^Cl)+H]^+^ and 215.0319 [M(^37^Cl)+H]^+^.

5-Fluoro-*N*-(1-methyl-1*H*-pyrazol-4-yl)-4-(1-methyl-1*H*-pyrazol-5-yl)pyrimidin-2-amine (**35**): Prepared from 2-Chloro-5-fluoro-4-(1-methyl-1*H*-pyrazol-5-yl)pyrimidine (**34**) (213 mg, 1.00 mmol) and 1-methyl-1*H*-pyrazole-4-amine (**13b**) (117 mg, 1.20 mmol) following the general synthetic procedure B. The residue was purified by flash column chromatography, eluting with 0–2.5% methanol in ethyl acetate to give **35** as a white solid (71 mg, 26%). m.p. 174.3–175.5 °C. ^1^H NMR (DMSO-*d*_6_): *δ* 3.82 (s, 3H, CH_3_); 4.16 (s, 3H, CH_3_); 6.84 (t, 1H, *J* = 2.0, pyrazolyl-H); 7.48 (s, 1H, pyrazolyl-H); 7.61 (d, 1H, *J* = 2.0, pyrazolyl-H); 7.86 (s, 1H, pyrazolyl-H); 8.59 (d, 1H, *J* = 2.9, pyrimidinyl-H); and 9.56 (s, 1H, NH). ^13^C NMR (DMSO-*d*_6_): *δ* 39.2, 39.4, 110.5 (d, *J*_C–F_ = 9.1), 121.4, 123.4, 130.7, 133.8, 138.5, 148.7 (d, *J*_C–F_ = 246.4), and 156.5 (two carbon signals overlapping or obscured). HRMS (ESI-TOF) 274.1209 [M + H]^+^; calcd. for C_12_H_13_FN_7_^+^ 274.1211 [M+H]^+^. Anal. RP/HPLC Method A: *t*_R_ 11.08 min, purity 100%; Method B: *t*_R_ 13.32 min, purity 100%.

### 4.3. Biology

#### 4.3.1. Kinase Assays

The luminescent ADP-Glo™ assay, which measures the amount of ADP formed in a kinase reaction as described previously [34], was used to determine the kinase inhibition profile of the compounds. Initially, the kinase inhibitory activity of the compounds was evaluated at 1 µM, and the compounds that displayed ≥ 80% kinase inhibition were selected for the determination of their apparent inhibition constant (*K*_i_) values. *K*_i_ values were calculated from their corresponding IC_50_ values (the concentrations of inhibitors that inhibit the kinase activity by 50%) and the Michaelis–Menten constant (*K*_m_) of ATP for each kinase using the Cheng–Prusoff equation as previously stated [34]. In brief, 1 μL of each concentration of the test compounds was added to the respective wells, and to it were added 1 μL of the kinases and their respective substrates, diluted to the desired concentration in kinase reaction buffer (40 nM tris base pH 7.5, 20 mM MgCl_2_, 0.4 µM dithiothreitol, and 0.1 mg/mL bovine serum albumin), and the pH was adjusted by adding 1 μL of a two-fold diluted kinase reaction buffer. To initiate the kinase activity, 1 μL of ATP solution (the concentration varies depending on the type of kinase under investigation) was added to each well, and the mixture was incubated at room temperature (20 to 22 °C) for 30–60 min depending on the kinase being tested. The reaction was then halted, 5 μL of the ADP-Glo reagent was added to each well (removing any unreacted ATP), and it was incubated at room temperature for 40 min. Then, to each well, 10 μL of the kinase detection reagent containing luciferin was added, which was incubated once more at room temperature in the dark for 30 min and the luminescence was measured by an EnVision^®^ multi-label plate reader with an integration time of 1.5 s per well. Positive controls (the wells without the inhibitor) and negative or blank controls (the wells without the inhibitor and the enzyme, but with 1 × kinase reaction buffer) were also determined in 2.5% DMSO.

The percentage of kinase inhibition was determined using the following equation:

$$\%\;Residual\;kinase\;activity=\frac{Luminescence\;in\;the\;presence\;of\;inhibitor-Luminescence\;of\;blank\;control}{Luminescence\;in\;the\;absence\;of\;inhibitor-Luminescence\;of\;blank\;control}\times 100\%\;\%kinase\;inhbition=100\%-\%Residual\;kinase\;activity$$

To determine the IC_50_ values, each compound was serially diluted in 2.5% DMSO, and the kinase inhibitory activities at eight different concentrations were determined and analyzed by a four-parameter logistic non-linear regression model using GraphPad Prism version 7.03 (San Diego, CA, USA). *K*_m_ is defined as the concentration of ATP or the substrate required to produce 50% of the maximal reaction rate in the kinase reaction. Therefore, since we used the concentration of ATP and the substrate at their *K*_m_ values, *K*_i_ simply equals IC_50_/2 assuming these inhibitors are ATP-competitive based on the Cheng–Prusoff equation: *K*_i_ = IC_50_/(1 + ([ATP]/*K*_m_(ATP))) where [ATP] is the ATP concentration used for the IC_50_ determination and *K*_m_(ATP) for each kinase was determined experimentally. 

#### 4.3.2. Cell Lines

Cancer cell lines derived from leukemia (MV4-11 and U937), breast (MCF7 (Rb-positive) and MDA-MB-231(Rb-negative)), and ovarian (A2780, OVCAR5, OAW28, OV90, Cov318, and Cov504) cancers, and glioblastoma (U87, T98G and U251) were acquired from the cell bank at Drug Discovery and Development, University of South Australia, and cultured according to the American Type Culture Collection (ATCC) recommendation in Roswell Park Memorial Institute (RPMI)-1640, Dulbecco’s Modified Eagle’s Medium (DMEM), or Minimum Essential Media (MEM), with 10% fetal bovine serum (Sigma-Aldrich, Castle Hill, NSW, Australia) within a humidified incubator at 37 °C in the presence of 5% CO_2_.

#### 4.3.3. Cell Viability Assays

The MTT (Life Technologies, Mulgrave, VIC, Australia) and resazurin (Sigma-Aldrich) assays were used to determine the antiproliferative activities of the compounds initially at 1 µM against the cancer cell lines stated above. Those compounds that inhibited the growth by ≥80% were subjected to the determination of GI_50_ using nonlinear regression analysis.

#### 4.3.4. Cell Cycle Analysis

The effect of the compounds on cell cycle distribution was evaluated following a previously reported method [34]. Briefly, six-well plates (Sigma-Aldrich, NSW, Australia) were seeded with about 1–2 × 10^5^ cells/well in 2 mL growth medium, incubated at 37 °C and 5% CO_2_ overnight, and treated with various concentrations of the test compounds and incubated for 48 h. The wells were collected by trypsinization, transferred into fluorescence-activating cell sorting (FACS) tubes, washed with 1 × phosphate-buffered saline (1 × PBS) twice, fixed in 70% (*v/v*) ice-cold ethanol for 15 min, and centrifuged at 300× *g* for 5 min. The cell pellets were then collected, stained with 200 µL of the cell cycle solution (comprising 50.0 μg/mL PI, 0.1 mg/mL RNase A, and 0.05% Triton X-100), and incubated for 1.5 h at room temperature in the dark. Finally, the DNA content was determined by a flow cytometer (CytoFLEX) and analyzed by CytExpert 2.1 software (Beckman Coulter, Brea, CA, USA). 

#### 4.3.5. Apoptosis Assay

Annexin V-fluorescein isothiocyanate (FITC) apoptosis detection kit I (consisting of a ten-time stock solution of binding buffer, FITC–annexin V, and PI staining solution) was used to determine the effect of the compounds on cancer cell survival as previously reported [34]. Briefly, six-well plates (Sigma-Aldrich, NSW, Australia) were seeded with about 1–2 × 10^5^ cells/well in 2 mL growth medium, incubated at 37 °C and 5% CO_2_ overnight, treated with various concentrations of the test compounds, and incubated for 48 h. The wells were collected by trypsinization, transferred into FACS tubes, washed twice with 1 × PBS, and centrifuged at 300× *g* for 5 min. The pellets were then suspended in 100 µL of 1 × binding buffer and incubated in the dark with 3 µL of PI and 3 µL of FITC-conjugated annexin V solutions for 15 min. Finally, 200 µL of binding buffer was added to each sample and analyzed by the CytoFLEX cytometer. The extent of apoptotic cell death was quantified as the sum of the percentages of early apoptotic cells (annexin V-positive and PI-negative) and late apoptotic cells (both annexin–V- and PI-positive) and presented graphically as a contour diagram relative to the untreated control.

#### 4.3.6. Colony Formation

The effect of compound **15** on colony formation was investigated as previously reported [34]. Briefly, ovarian cancer cells (i.e., A2780, OVCAR5, and OV90) seeded in six-well plates were adhered for 6 h and treated with **15**. The medium that contained the compound was then changed every 72 h. Ten days after treatment, the cells were washed with PBS, fixed, and stained with crystal violet staining solution (0.05% crystal violet, 1% formaldehyde, 1% PBS, and 1% ethanol), and colonies were quantified using ImageJ software (National Institute of Health, Maryland, USA), with data presented as percentages of the control cells.

#### 4.3.7. Western Blot Analysis

Western blot analysis of the compounds was carried out as previously reported [35]. The antibodies were purchased from Cell Signaling Technology (Danvers, MA, USA). 

## Figures and Tables

**Table 1:**
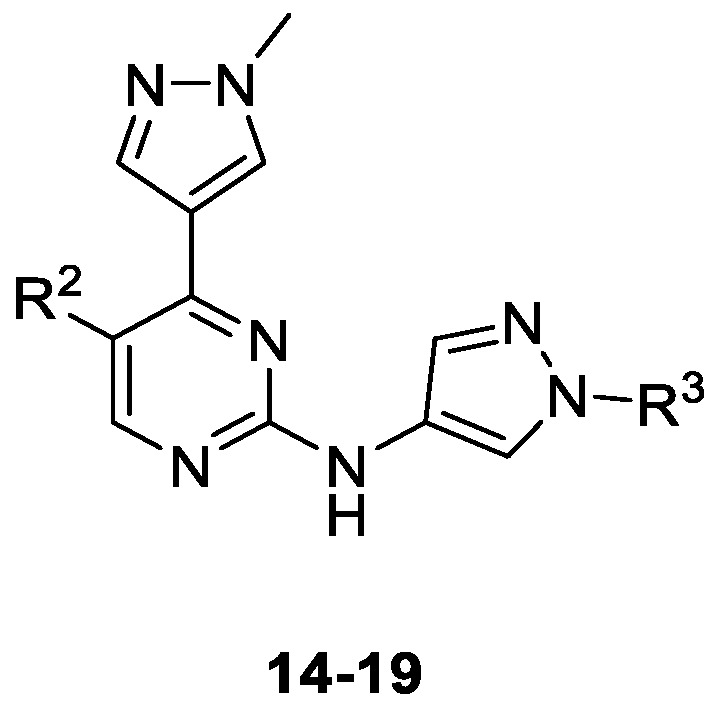
Inhibition of CDK kinase activity and A2780 cell viability by **14**–**19**.

Compound	Substituent		*K*_i_ (µM) or % Enzymatic Inhibition at 1 µM *				GI_50_ (µM) or % Growth Inhibition (GI) at 1 µM *
	R^2^	R^3^	CDK1B	CDK2E	CDK5p25	CDK9T1	A2780
**14**	F	H	0.018	0.007	0.003	0.016	0.498
**15**	Cl	H	0.025	0.005	0.003	0.020	0.158
**16**	F	Me	0.092	0.023	0.018	0.098	9 *
**17**	Cl	Me	0.034	0.011	0.006	0.056	1.023
**18**	F	CH_2_CONH_2_	0.112	0.085	0.193	68*	0 *
**19**	Cl	CH_2_CONH_2_	0.018	0.025	0.052	0.101	44 *
**1**	-		0.005	0.023	0.077	0.026	0.018

The enzymatic inhibition percentage was determined using a luminescent ADP-Glo™ assay. Apparent inhibition constant (*K*_i_) values were calculated using their corresponding half maximal inhibition (IC_50_) values and the appropriate *K*_m_(ATP) of each kinase as described in the experimental section. The antiproliferative activity was determined by 72 h 3-(4,5-dimethylthiazol-2-yl)-2,5-diphenyltetrazolium bromide (MTT) assays using A2780 cells. The asterisk (*) indicates % for either biochemical or cell-based data. Previously reported data for **1** [20] were included in the table for comparison purposes. CDK1B, CDK2E, CDK5p25, and CDK9T1 denote CDK1/cyclin B, CDK2/cyclin E, CDK5/p25, and CDK9/cyclin T1, respectively. Me: methyl.

**Table 2:**
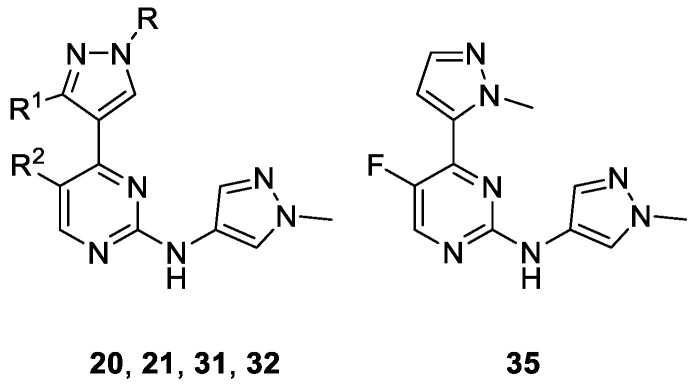
Inhibition of CDK kinase activity and A2780 cell viability by **20**, **21**, **31**, **32**, and **35**.

Compound	Substituent			*K*_i_ (µM) or % Enzymatic Inhibition at 1 µM *				%GI at 1 µM *
	R	R^1^	R^2^	CDK1B	CDK2E	CDK5p25	CDK9T1	A2780
**20**	Me	Me	F	0.164	0.026	0.018	0.343	7 *
**21**	Me	Me	Cl	0.225	0.023	0.035	0.062	8 *
**31**	2-morpholinoethyl	H	F	26 *	48 *	43 *	24 *	0 *
**32**	2-morpholinoethyl	H	Cl	36 *	0.252	0.126	21 *	3 *
**35**	-			49 *	64 *	58 *	45 *	19 *

The enzymatic inhibition percentage was determined using the luminescent ADP-Glo™ assay. Apparent inhibition constant (*K*_i_) values were calculated using their corresponding half maximal inhibition (IC_50_) values and the appropriate *K*_m_ (ATP) of each kinase as described in the experimental section. The antiproliferative activity was determined by 72 h MTT assays using A2780 cells. The asterisk (*) indicates % for either biochemical or cell-based data. CDK1B, CDK2E, CDK5p25, and CDK9T1 denote CDK1/cyclin B, CDK2/cyclin E, CDK5/p25, and CDK9/cyclin T1, respectively. Me: methyl.

**Table 3:**
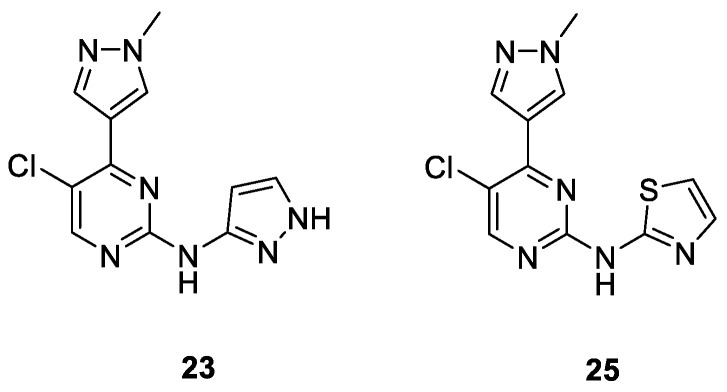
Inhibition of CDK kinase activity and A2780 cell viability by **23** and **25**.

Compound	*K*_i_ (µM) or % Enzymatic Inhibition at 1 µM *				GI_50_ (µM) or %GI at 1 µM *
	CDK1B	CDK2E	CDK5p25	CDK9T1	A2780
**23**	9 *	0.090	0.040	40 *	7.350
**25**	31 *	46 *	0.177	31 *	9 *

The enzymatic inhibition percentage was determined using the luminescent ADP-Glo™ assay. Apparent inhibition constant (*K*_i_) values were calculated using their corresponding half maximal inhibition (IC_50_) values and the appropriate *K*_m_ (ATP) of each kinase as described in the experimental section. The antiproliferative activity was determined by 72 h MTT assays using A2780 cells. The asterisk (*) indicates % for either biochemical or cell-based data. CDK1B, CDK2E, CDK5p25, and CDK9T1 denote CDK1/cyclin B, CDK2/cyclin E, CDK5/p25, and CDK9/cyclin T1, respectively.

**Table 4:** Antiproliferative effects of **15**.

Cancer Type	Cancer Cell Line	Antiproliferative Activity, GI_50_ (µM)
Leukemia	MV4-11	0.127
	U937	0.186
Breast	MCF7	0.342
	MDA-MB-231	0.458
Ovarian	OVCAR5	0.150
	OAW28	0.534
	OV90	0.390
	Cov318	0.488
	Cov504	0.540
Glioblastoma	U87	0.560
	T98G	0.380
	U251	0.350

## Data Availability

Not applicable.

## Supplementary Materials

The following supporting information can be downloaded at: https://www.mdpi.com/article/10.3390/molecules28072951/s1, The supplementary data (Figures S1–S65) related to this article encompasses the ^1^H & ^13^C NMR spectra, HRMS & HPLC chromatograms of **14**–**21**, **23**, **25**, **31**, **32** and **35**.
