# Supplementary material for: Discovery of N,4-Di(1H-pyrazol-4-yl)pyrimidin-2-amine-Derived CDK2 Inhibitors as Potential Anticancer Agents: Design, Synthesis, and Evaluation
Source: Molecules. 2023 Mar 25;28(7):2951. doi: 10.3390/molecules28072951 (PMC10096391; doi:10.3390/molecules28072951)
Supplement: Supplementary file 1 [file molecules-28-02951-s001.zip › molecules-2289918-supplementary.pdf]

Supplementary Data

Discovery of *N*,4-Di(1*H*-pyrazol-4-yl)pyrimidin-2-amine-Derived CDK2 Inhibitors as Potential Anticancer Agents: Design, Synthesis & Evaluation

Biruk Sintayehu Fanta, Jimma Lenjisa, Theodosia Teo, Lianmeng Kou, Laychiluh Mekonnen, Yuchao Yang, Sunita KC Basnet, Ramin Hassankhani, Matthew J. Sykes, Mingfeng Yu* and Shudong Wang*

Drug Discovery and Development, Clinical and Health Sciences, University of South Australia, Adelaide, South Australia 5000, Australia.

***Corresponding authors. E-mail: Mingfeng.Yu@unisa.edu.au; Shudong.Wang@unisa.edu.au**

Content

Figures S1-S65. ^{1}H & ^{13}C NMR spectra, HRMS & HPLC chromatograms of **14-21**, **23, 25**, **31**, **32** and **35**.

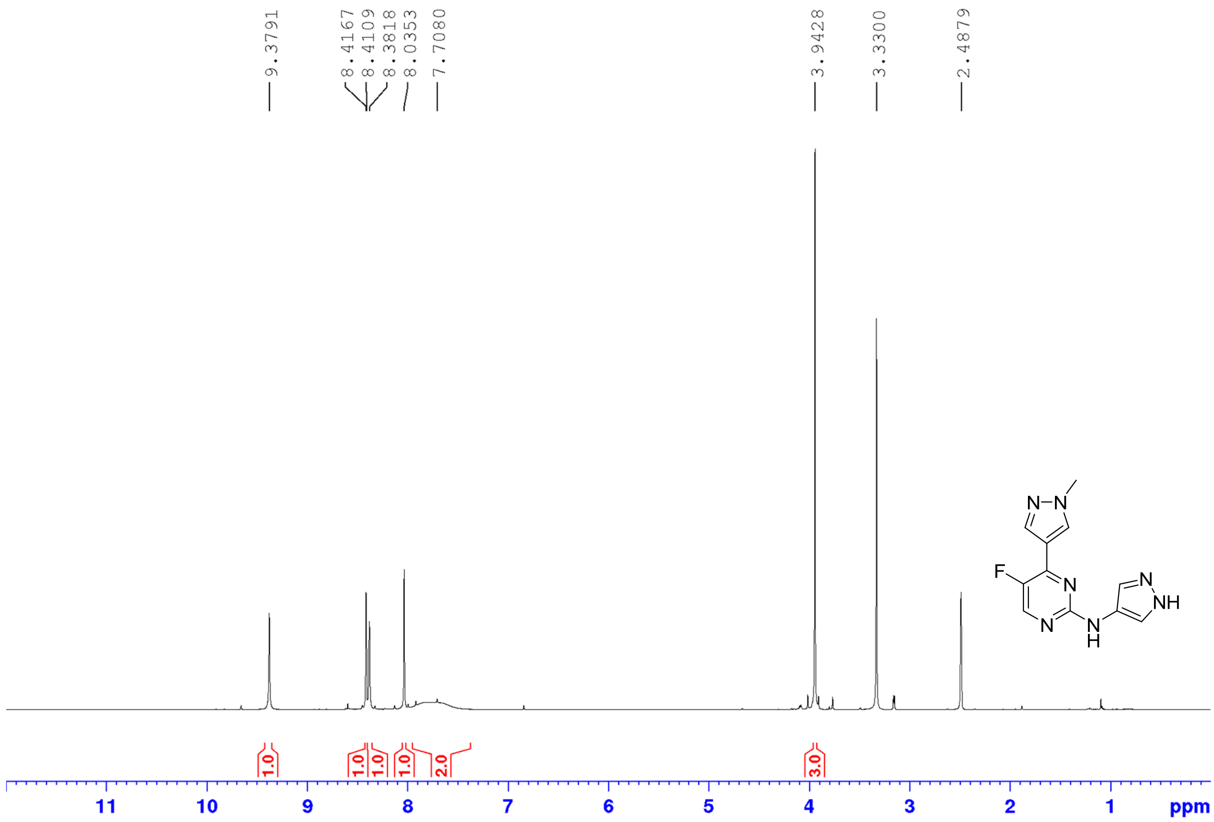

Figure S1. ^{1}H NMR spectrum of **14** in DMSO-d_6 (500 MHz).

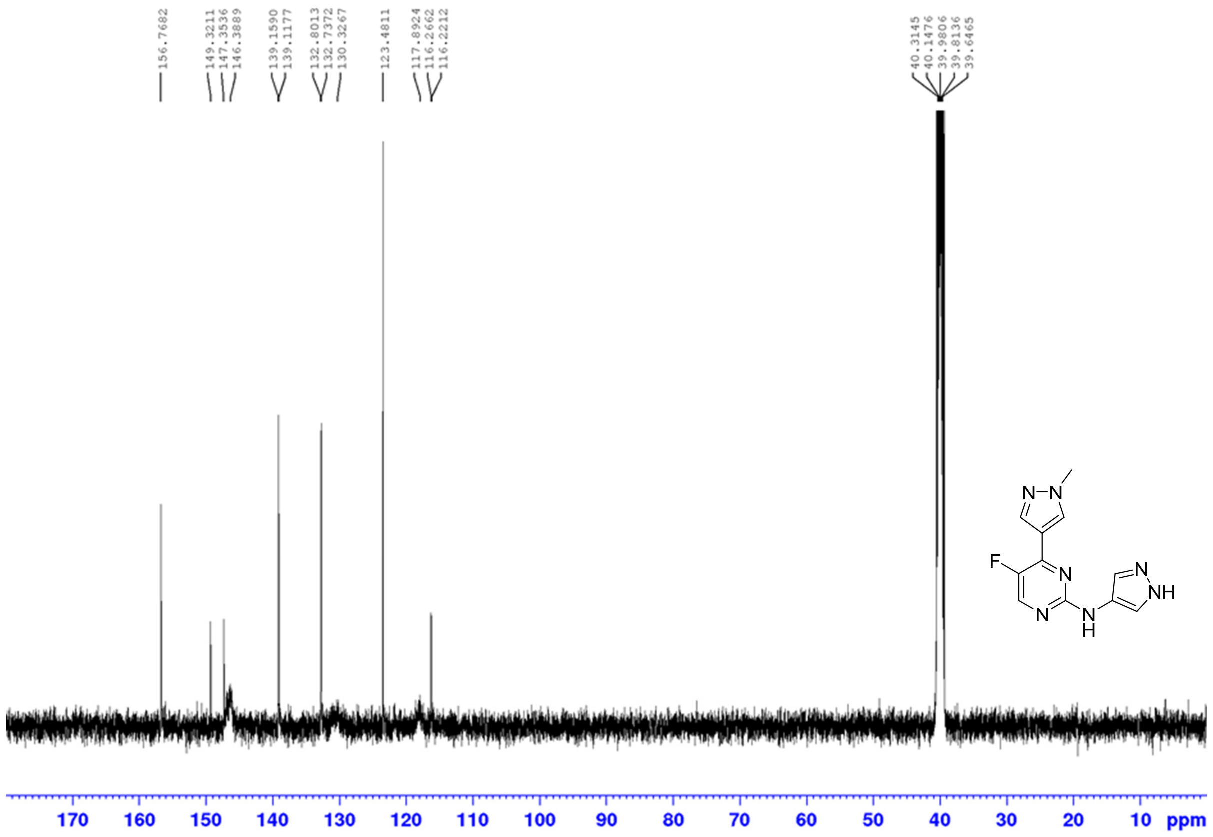

Figure S2. ^{13}C NMR spectrum of **14** in DMSO-d_6 (125 MHz).

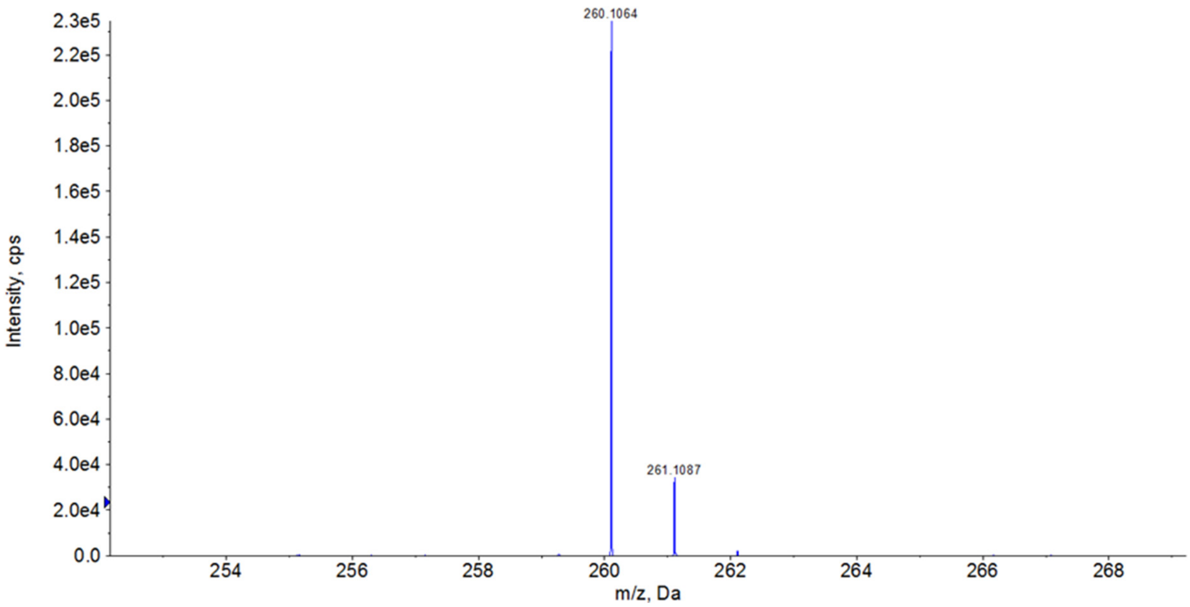

Figure S3. HRMS of **14**.

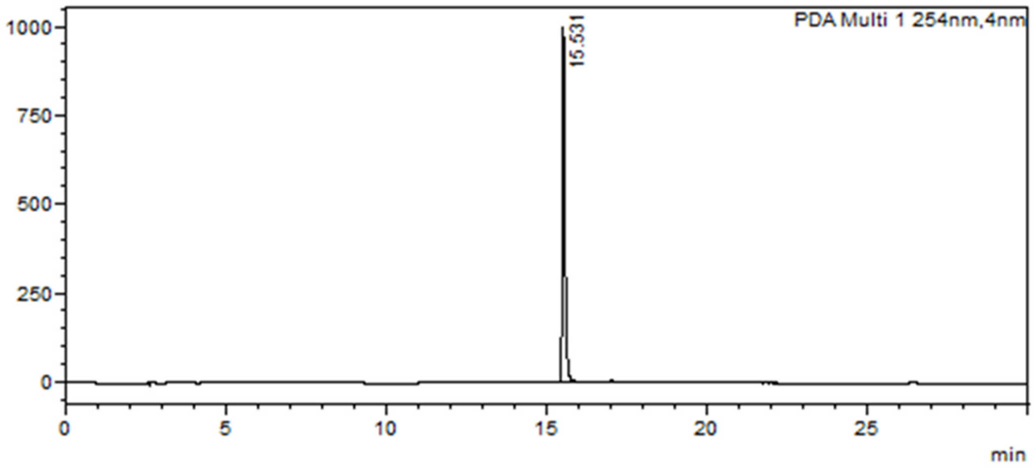

<Peak Table>

PDA Ch1 254nm

Peak#	Ret. Time	Area	Height	Conc.	Unit	Mark	Name
1	15.531	5743196	997219	100.000			
Total		5743196	997219				

Figure S4. HPLC chromatogram of **14** determined by method A.

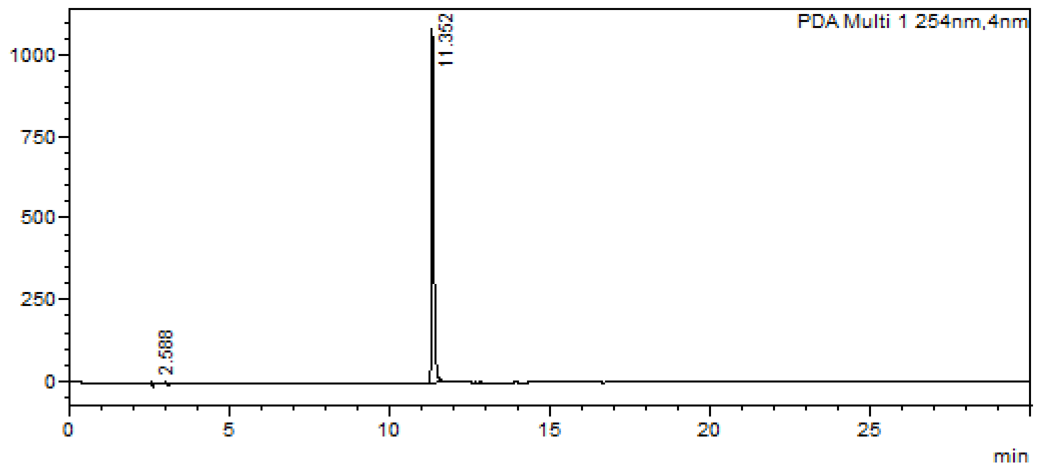

<Peak Table>

PDA Ch1 254nm

Peak#	Ret. Time	Area	Height	Conc.	Unit	Mark	Name
1	2.588	47004	14805	0.841			
2	11.352	5540735	1078809	99.159			
Total		5587739	1093614				

Figure S5. HPLC chromatogram of **14** determined by method B.

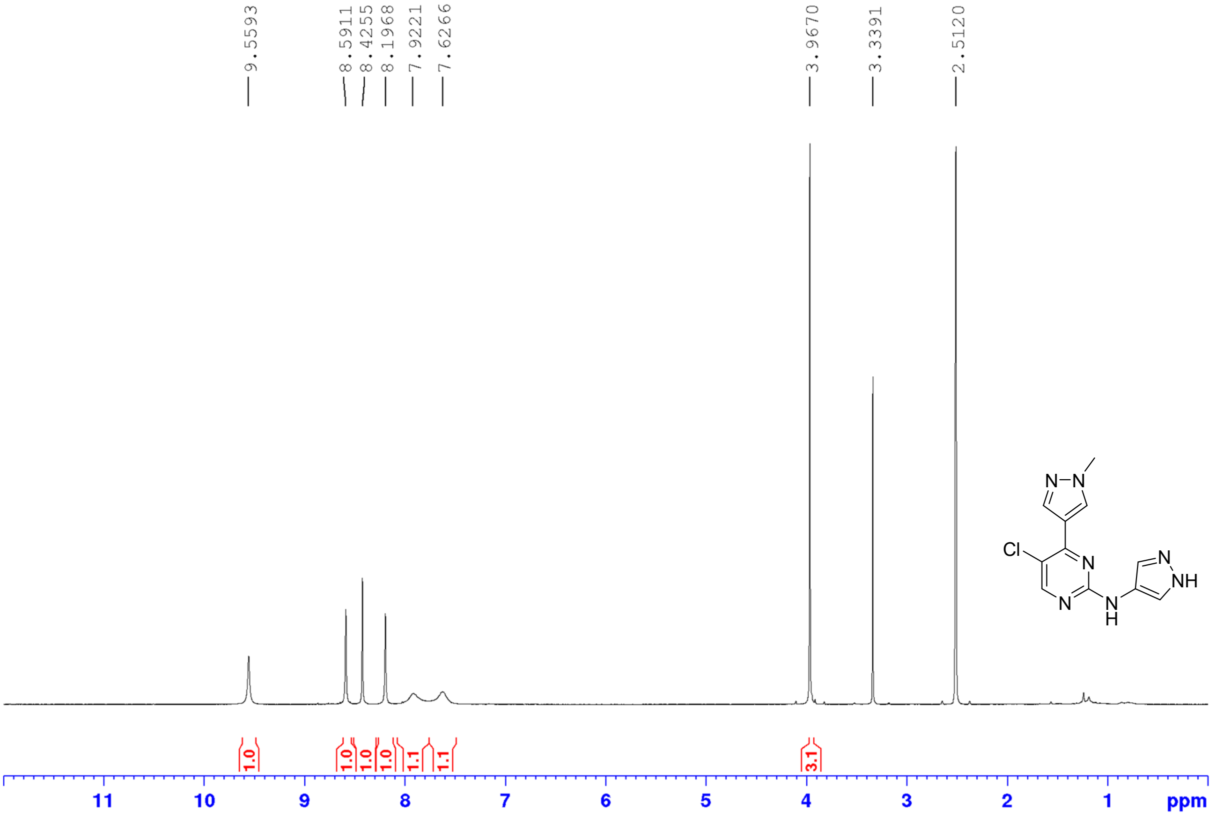

Figure S6. ^{1}H NMR spectrum of **15** in DMSO-d_6 (500 MHz).

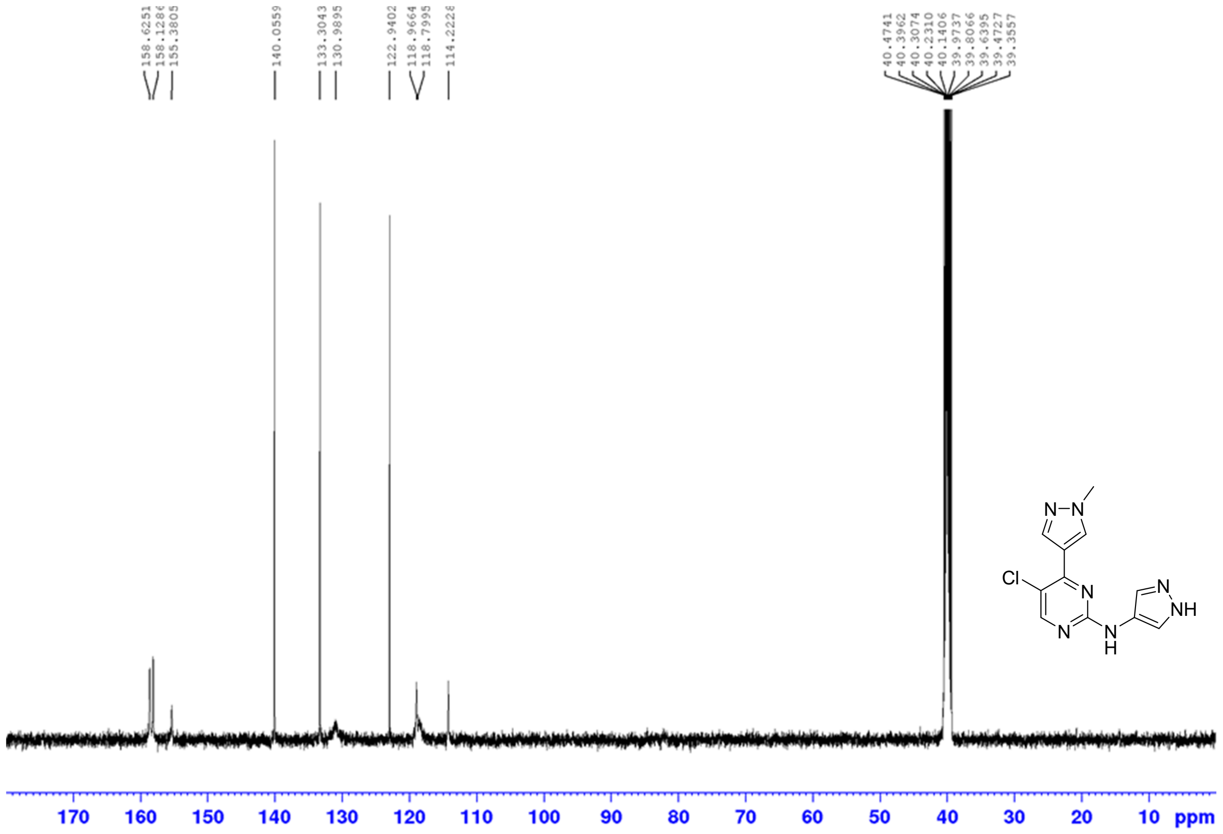

Figure S7. ^{13}C NMR spectrum of **15** in DMSO-d_6 (125 MHz).

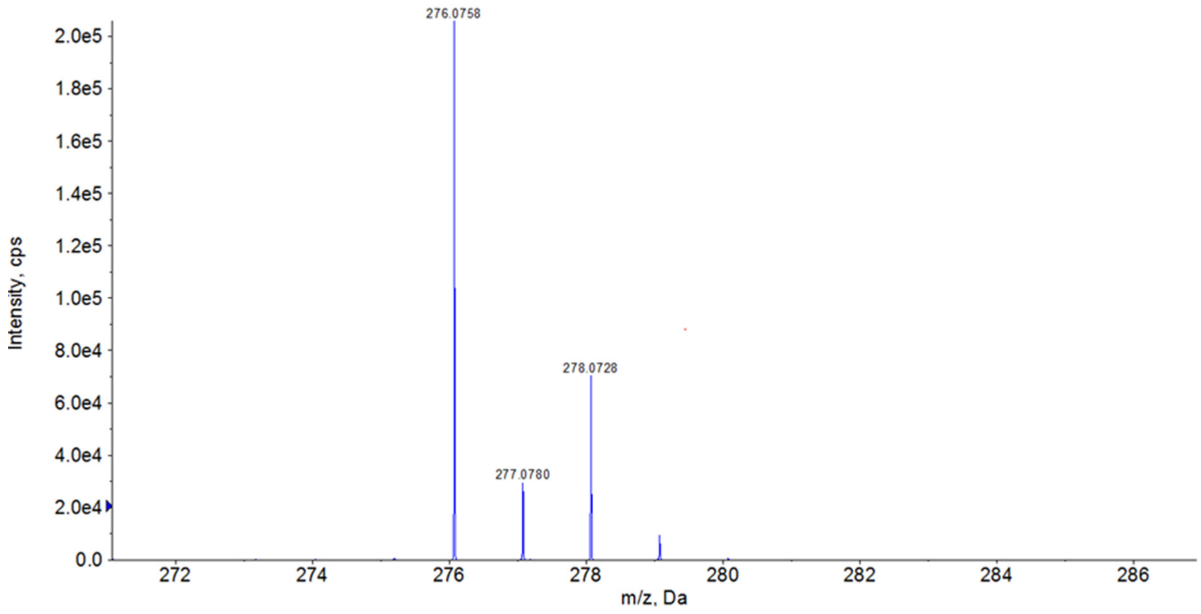

Figure S8. HRMS of **15**.

<Chromatogram>

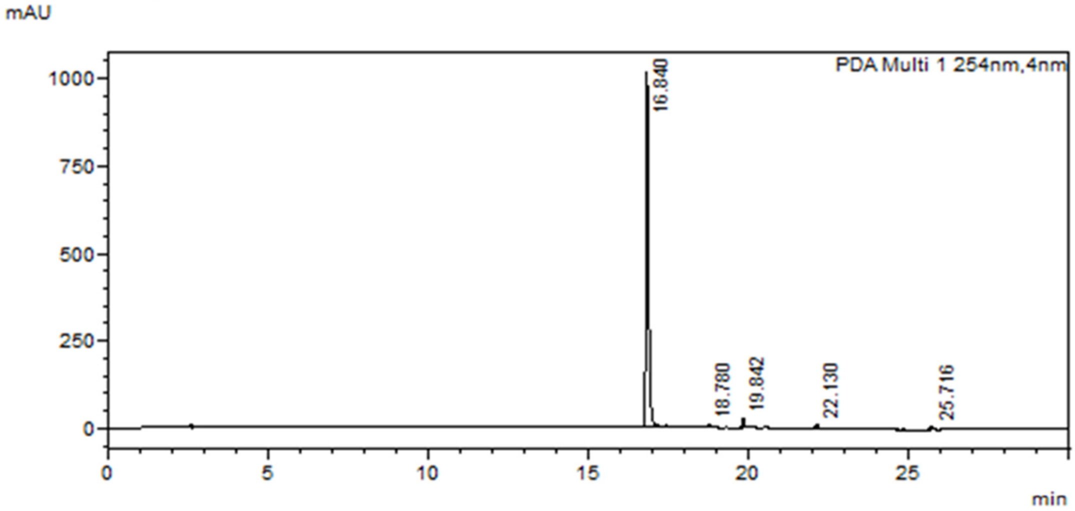

<Peak Table>

PDA Ch1 254nm

Peak#	Ret. Time	Area	Height	Conc.	Unit	Mark	Name
1	16.840	5781709	1012524	95.582			
2	18.780	55656	10458	0.920			
3	19.842	123205	24487	2.037			
4	22.130	54164	11585	0.895			
5	25.716	34230	7150	0.566			
Total		6048964	1066205				

Figure S9. HPLC chromatogram of **15** determined by method A.

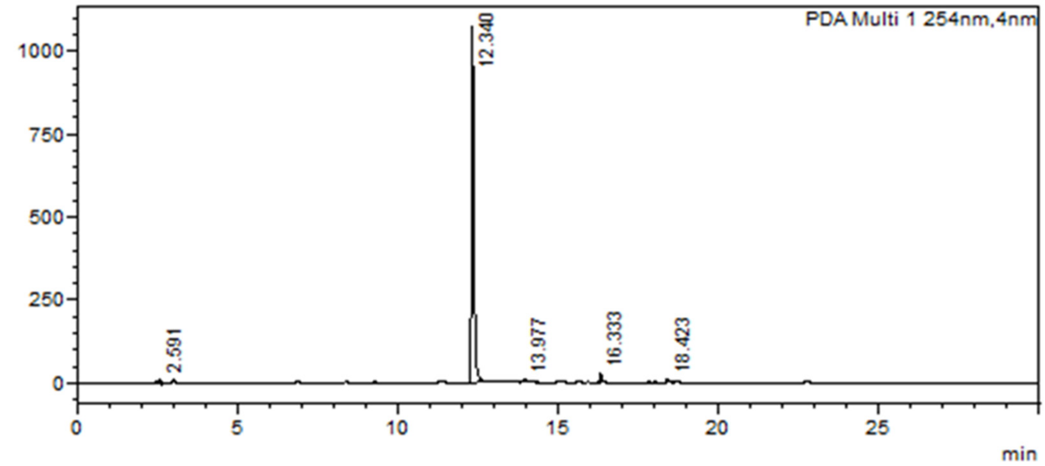

<Peak Table>

PDA Ch1 254nm

Peak#	Ret. Time	Area	Height	Conc.	Unit	Mark	Name
1	2.591	43150	15123	0.741			
2	12.340	5563012	1069367	95.547			
3	13.977	55892	10403	0.960			
4	16.333	114395	25665	1.965			
5	18.423	45799	8813	0.787			
Total		5822248	1129370				

Figure S10. HPLC chromatogram of **15** determined by method B.

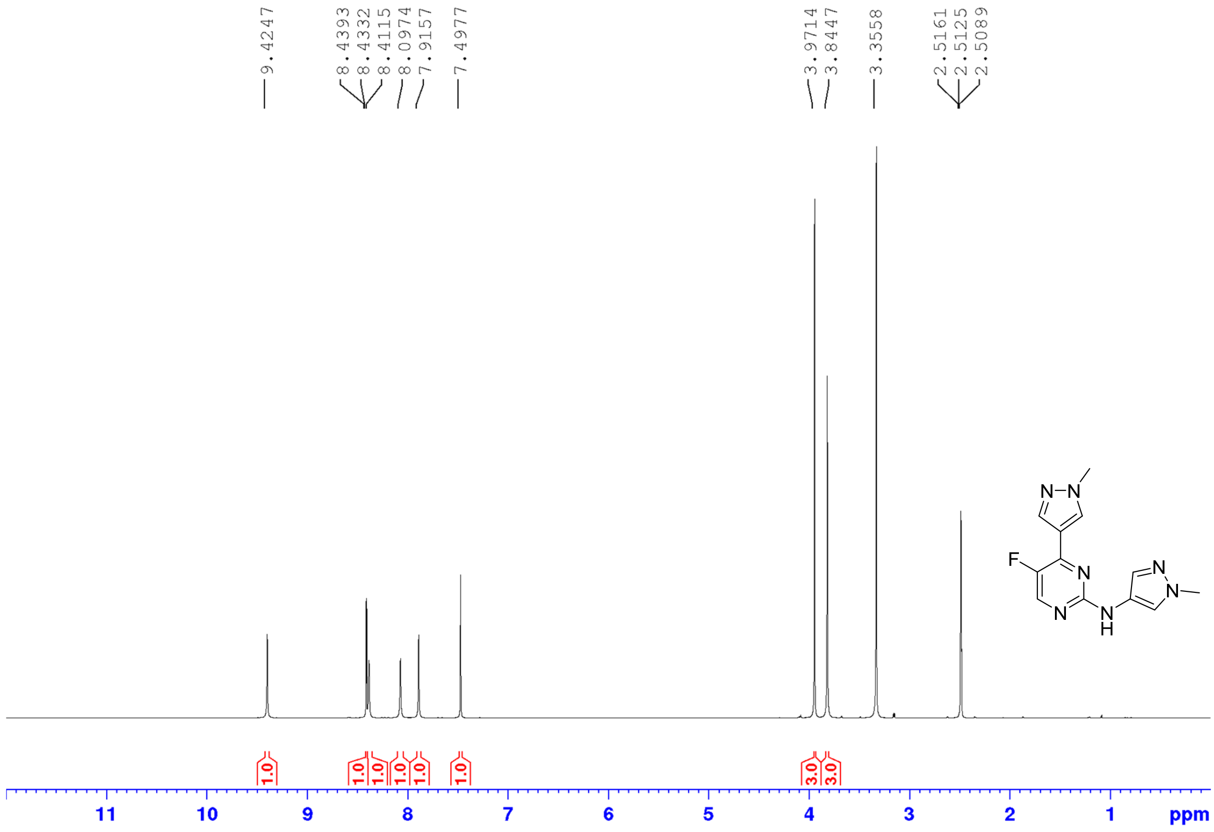

Figure S11. ^{1}H NMR spectrum of **16** in DMSO-d_6 (500 MHz).

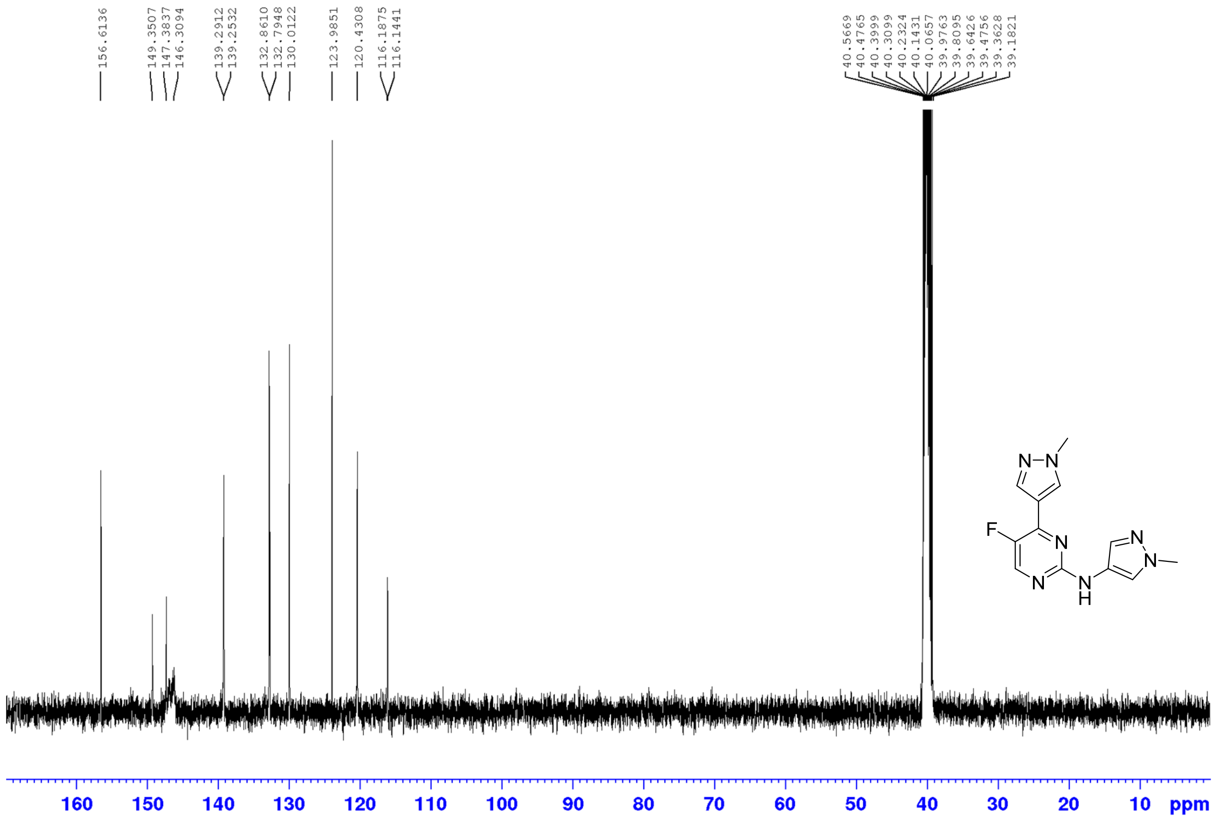

Figure S12. ^{13}C NMR spectrum of **16** in DMSO-d_6 (125 MHz).

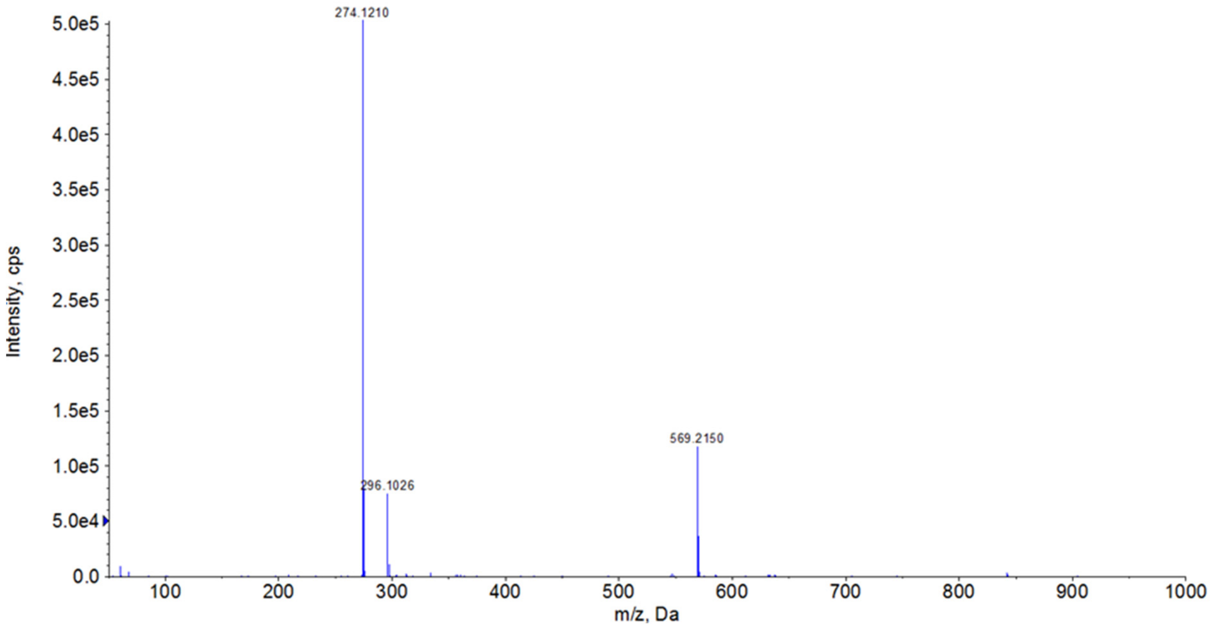

Figure S13. HRMS of **16**.

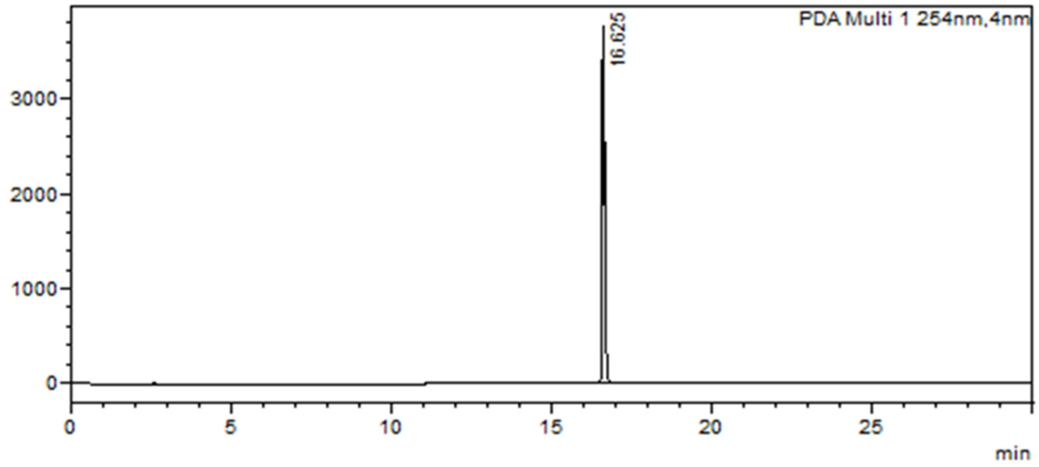

<Peak Table>

PDA Ch1 254nm

Peak#	Ret. Time	Area	Height	Conc.	Unit	Mark	Name
1	16.625	20762941	3757493	100.000			
Total		20762941	3757493				

Figure S14. HPLC chromatogram of **16** determined by method A.

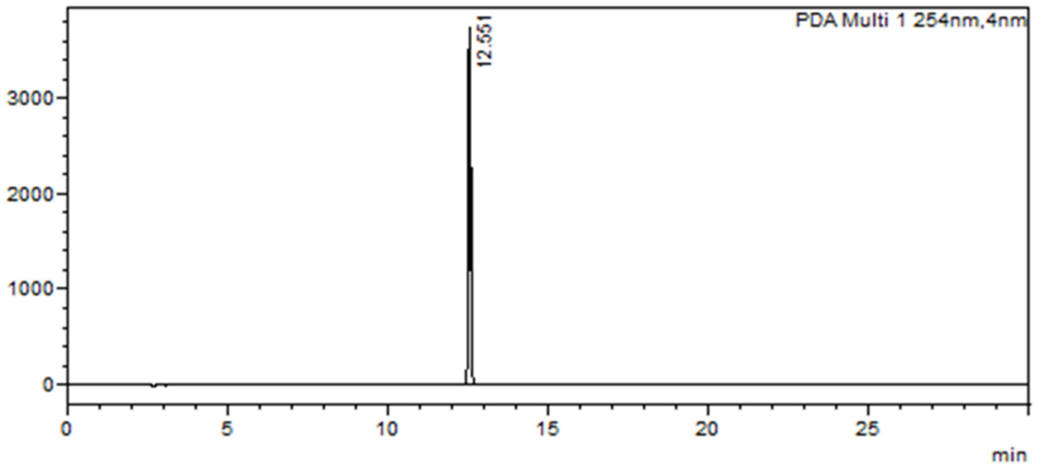

<Peak Table>

PDA Ch1 254nm

Peak#	Ret. Time	Area	Height	Conc.	Unit	Mark	Name
1	12.551	18116817	3731671	100.000			
Total		18116817	3731671				

Figure S15. HPLC chromatogram of **16** determined by method B.

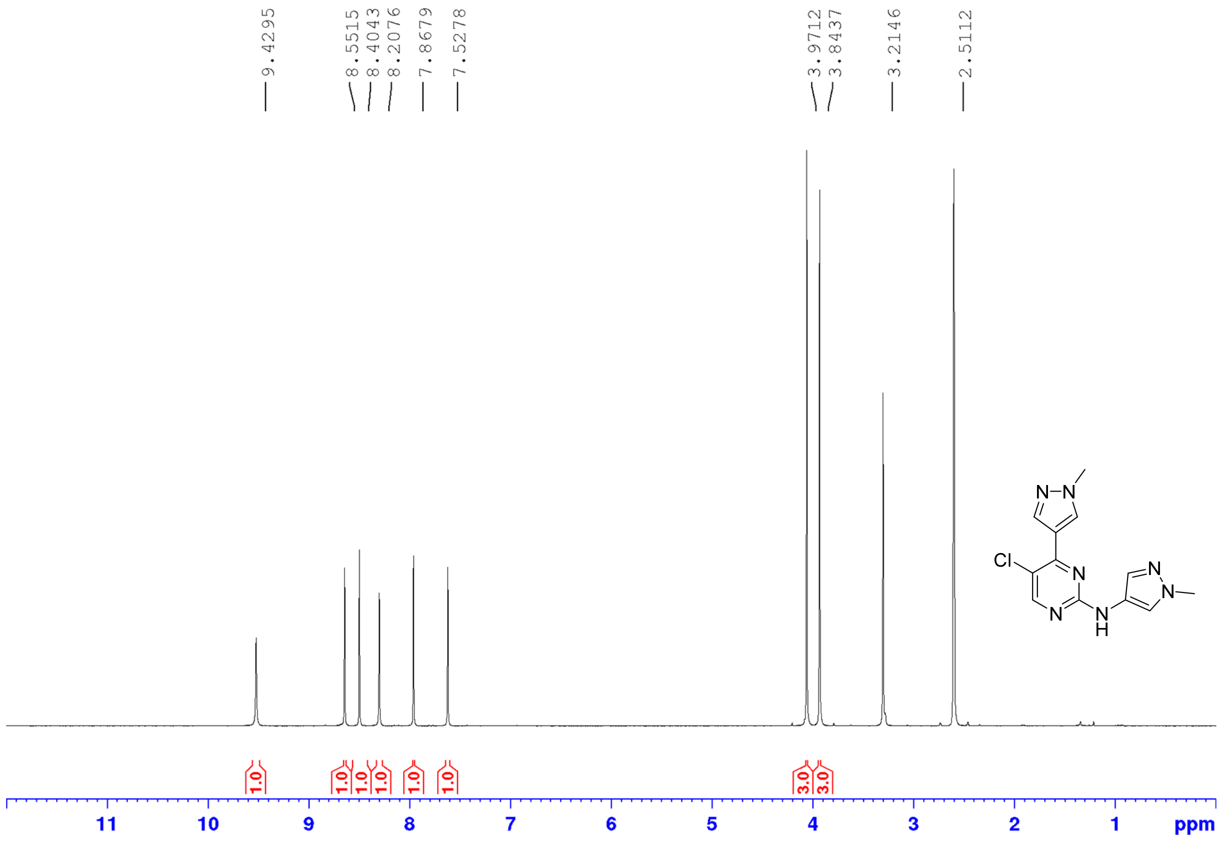

Figure S16. ^{1}H NMR spectrum of **17** in DMSO-d_6 (500 MHz).

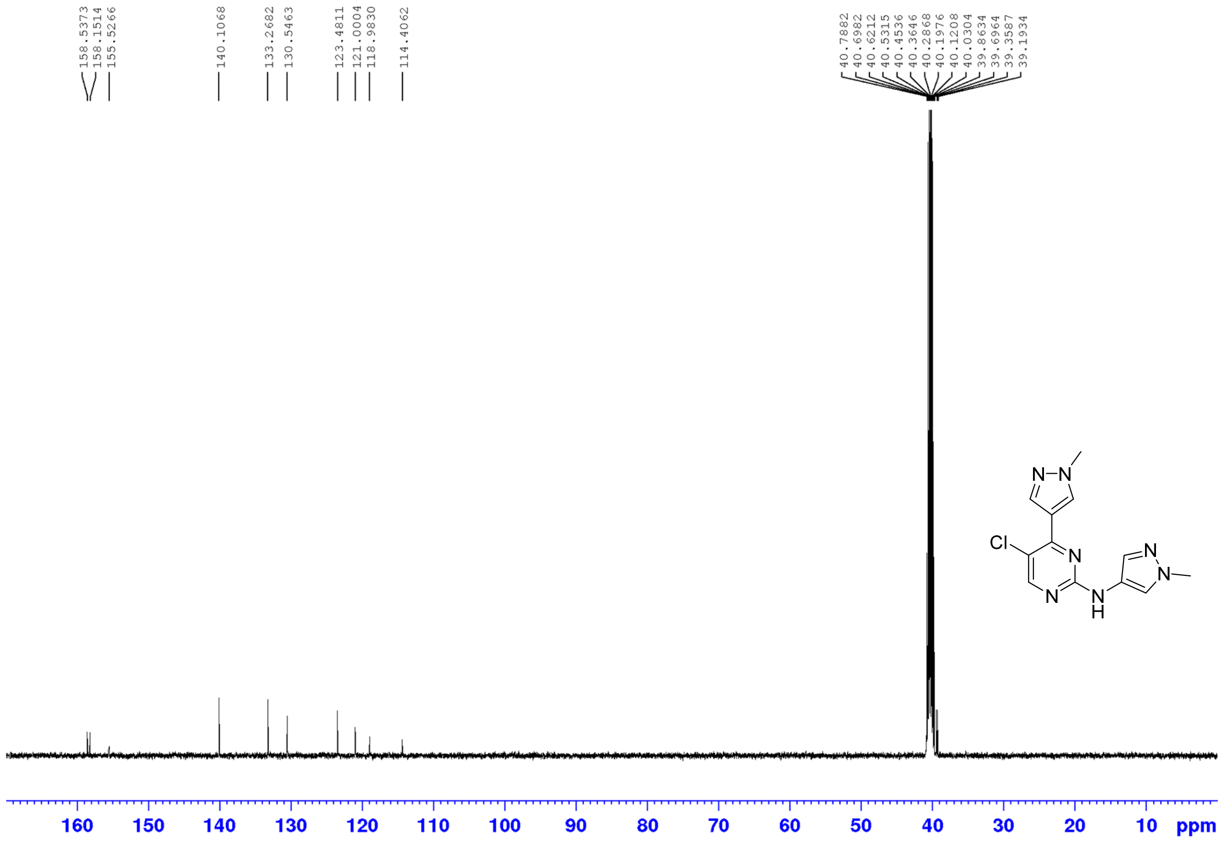

Figure S17. ^{13}C NMR spectrum of **17** in DMSO-d_6 (125 MHz).

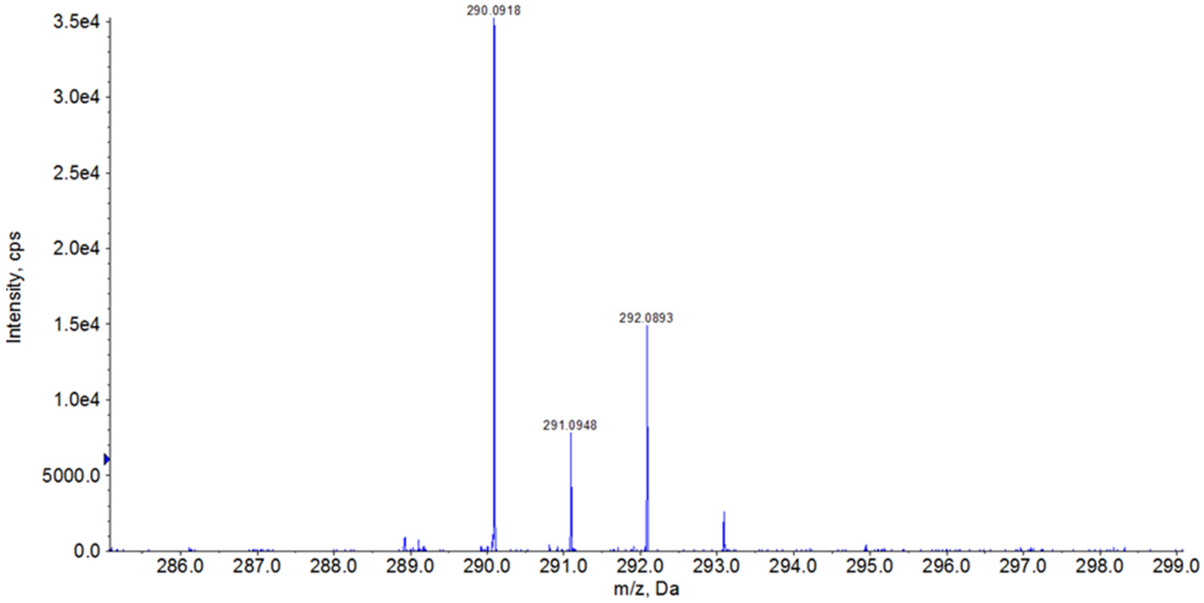

Figure S18. HRMS of **17**.

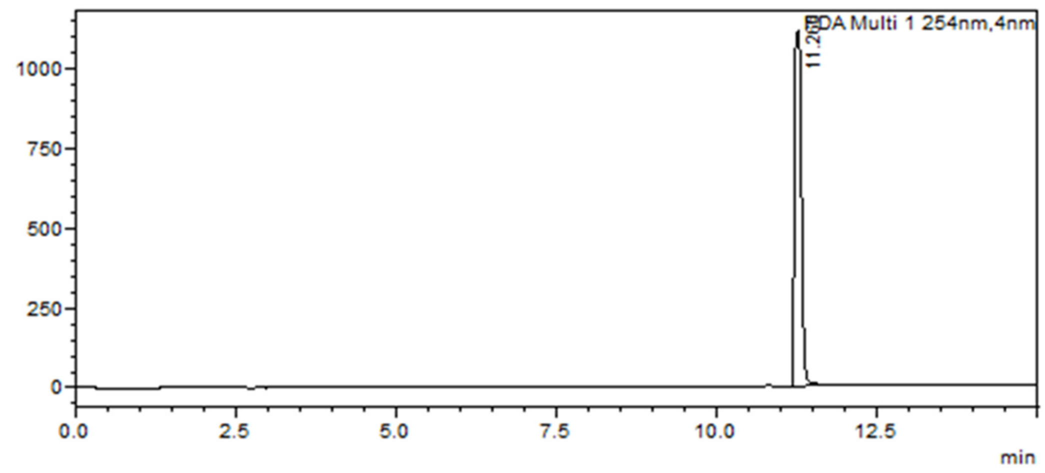

<Peak Table>

PDA Ch1 254nm

Peak#	Ret. Time	Area	Height	Conc.	Unit	Mark	Name
1	11.265	7841095	1111890	100.000			
Total		7841095	1111890				

Figure S19. HPLC chromatogram of **17** determined by method A.

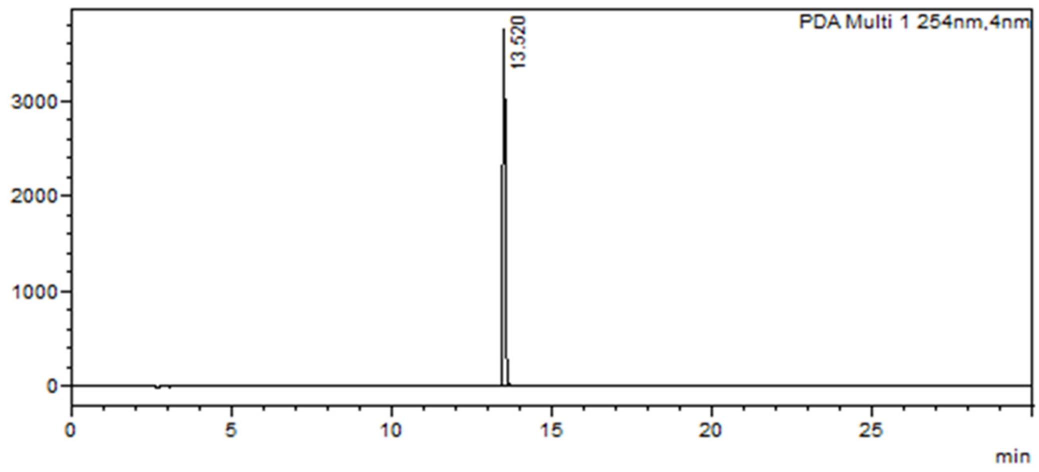

<Peak Table>

PDA Ch1 254nm

Peak#	Ret. Time	Area	Height	Conc.	Unit	Mark	Name
1	13.520	19824387	3750376	100.000			
Total		19824387	3750376				

Figure S20. HPLC chromatogram of **17** determined by method B.

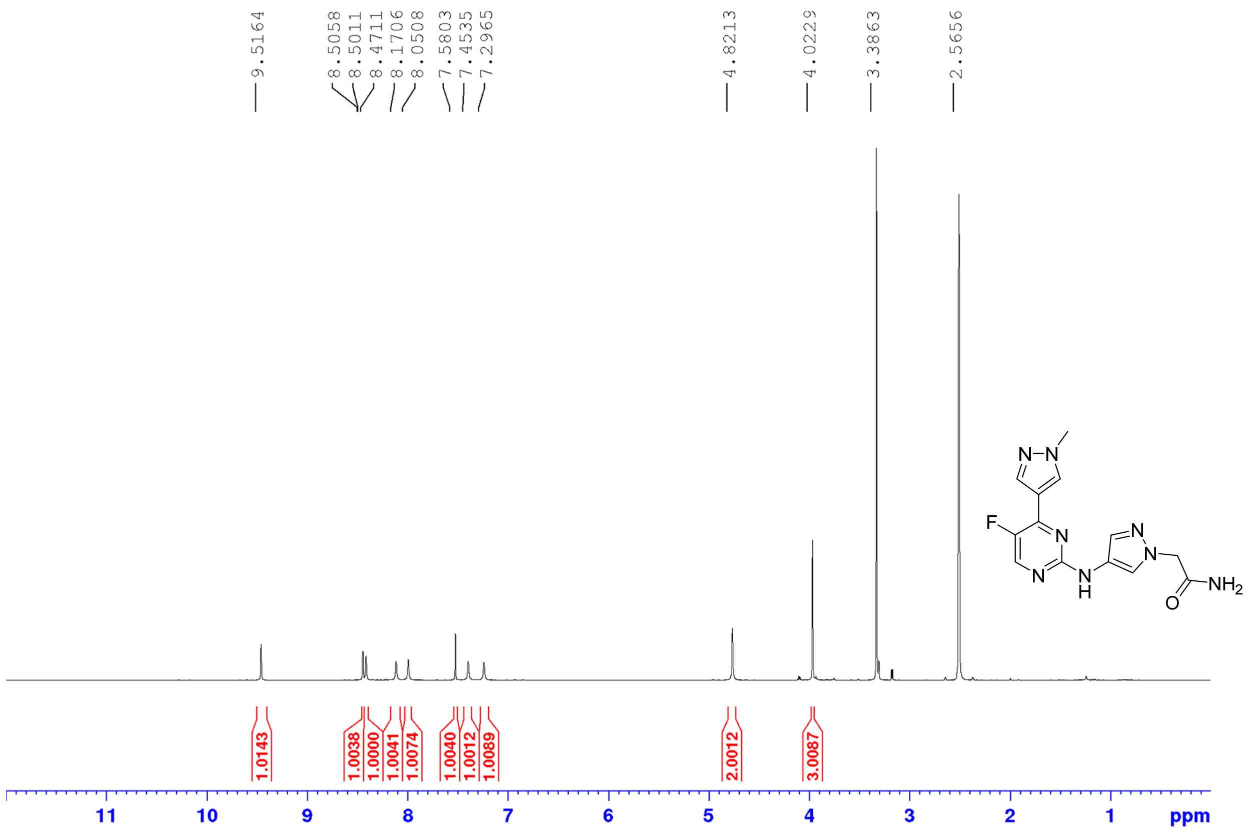

Figure S21. ^{1}H NMR spectrum of **18** in DMSO-d_6 (500 MHz).

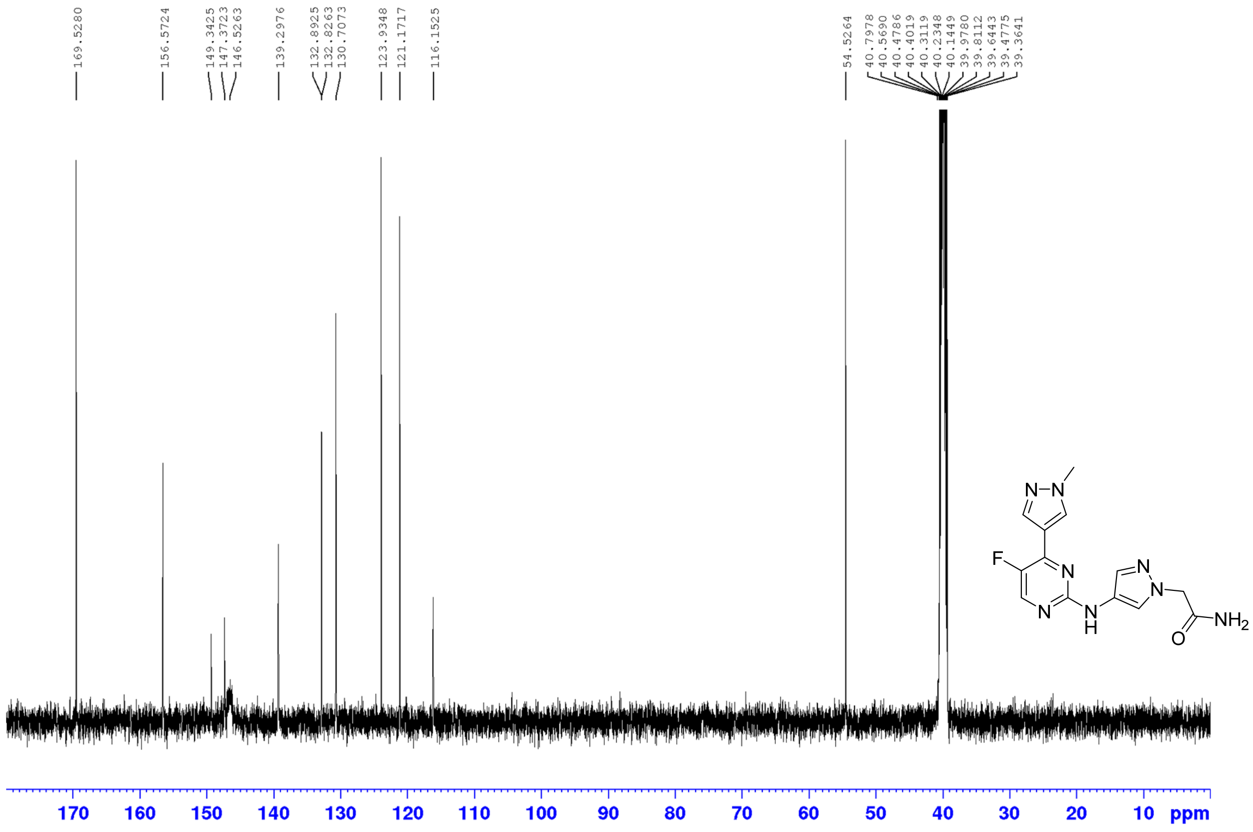

Figure S22. ^{13}C NMR spectrum of **18** in DMSO-d_6 (125 MHz).

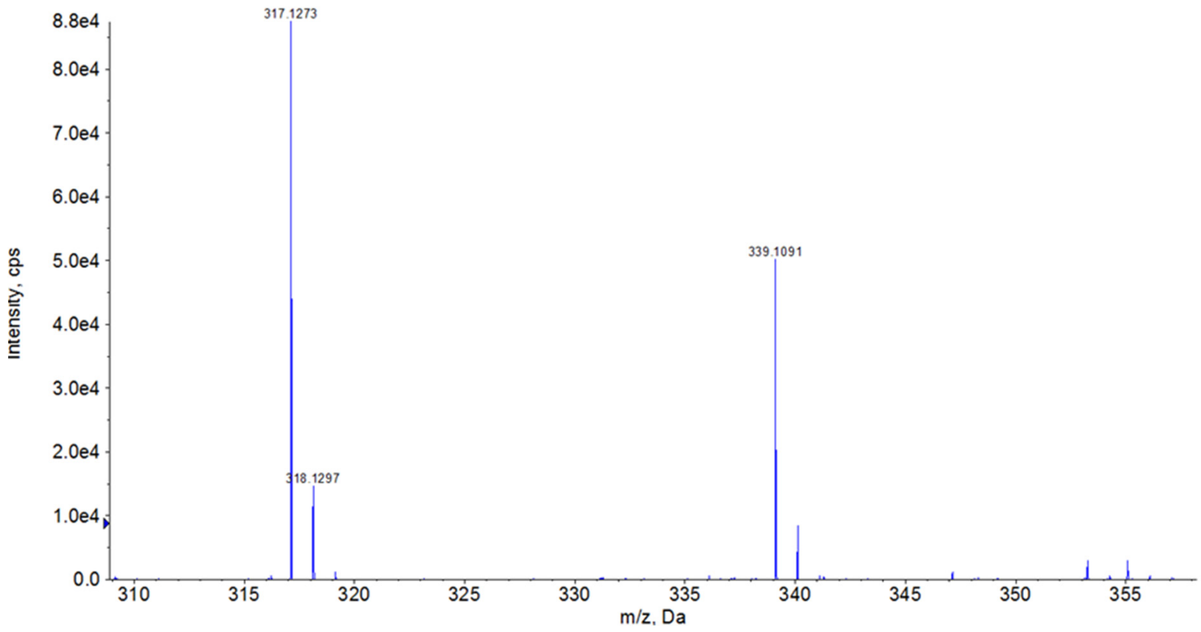

Figure S23. HRMS of **18**.

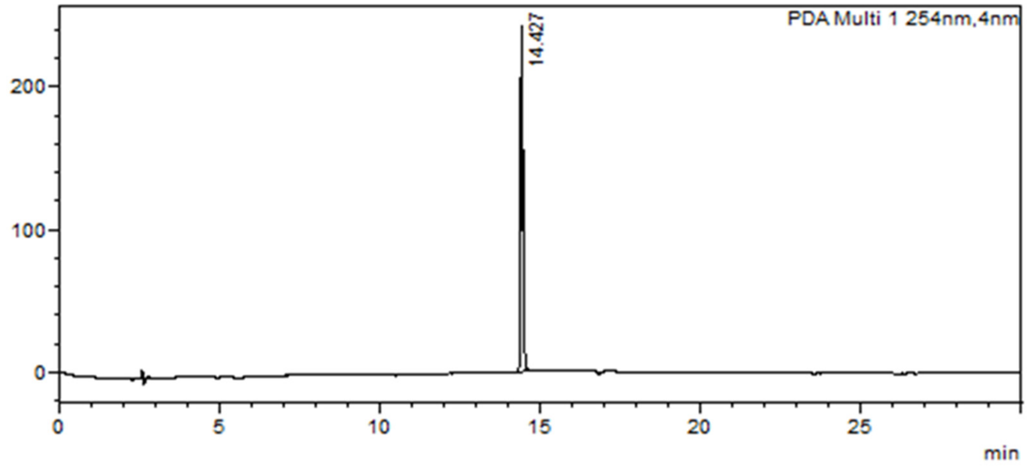

<Peak Table>

PDA Ch1 254nm

Peak#	Ret. Time	Area	Height	Conc.	Unit	Mark	Name
1	14.427	1192119	241524	100.000			
Total		1192119	241524				

Figure S24. HPLC chromatogram of **18** determined by method A.

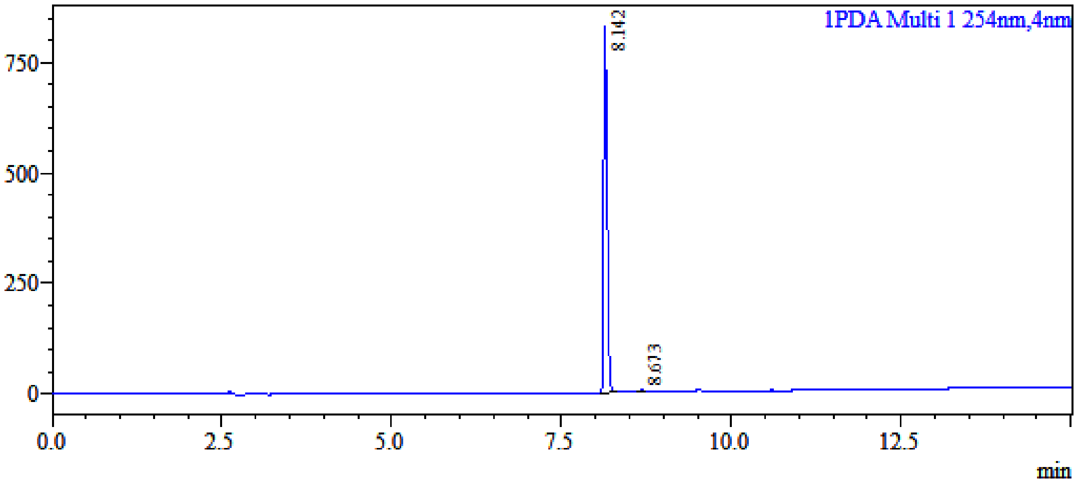

Peak Table

PDA Ch1 254nm

Peak#	Ret. Time	Area	Height	Conc.	Name	Area%
1	8.142	2923167	826705	99.344		99.344
2	8.673	19317	5928	0.656		0.656
Total		2942484	832633			100.000

Figure S25. HPLC chromatogram of **18** determined by method B.

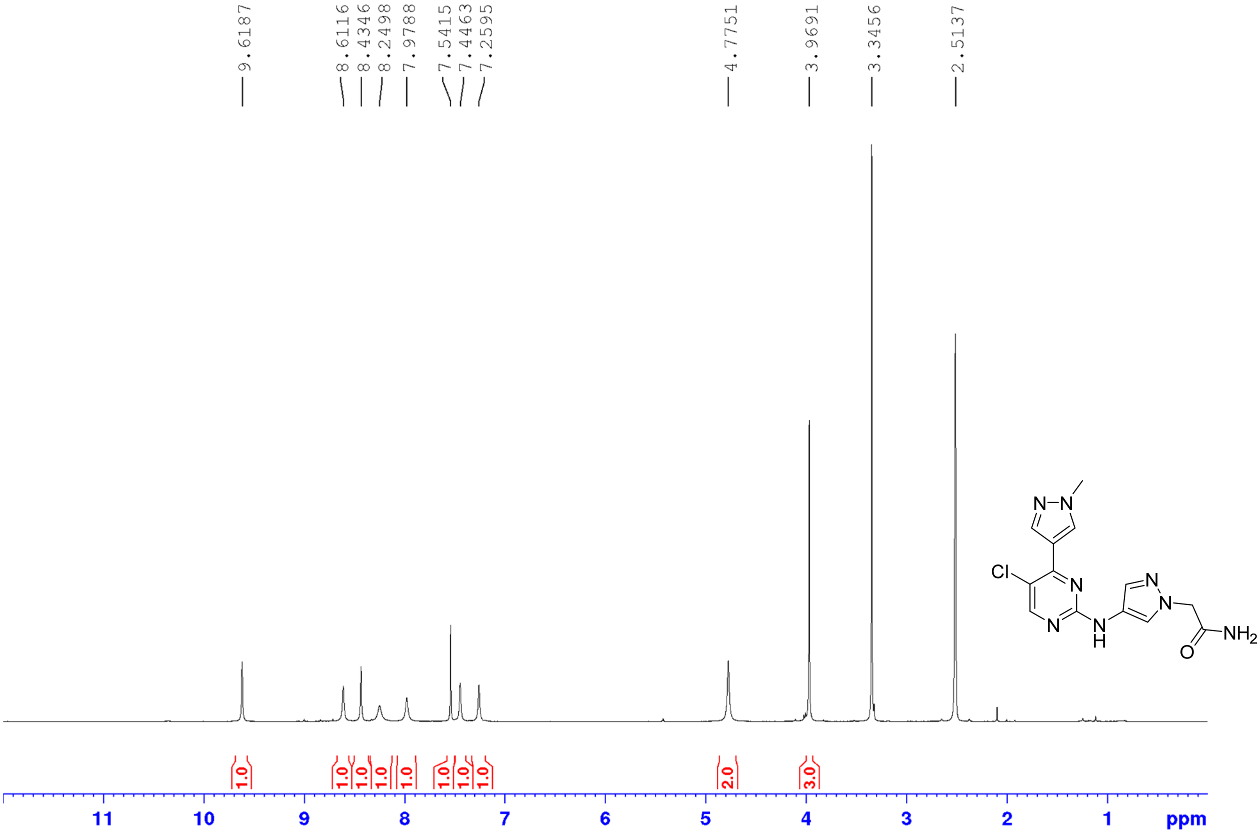

Figure S26. ¹H NMR spectrum of **19** in DMSO-d_6 (500 MHz).

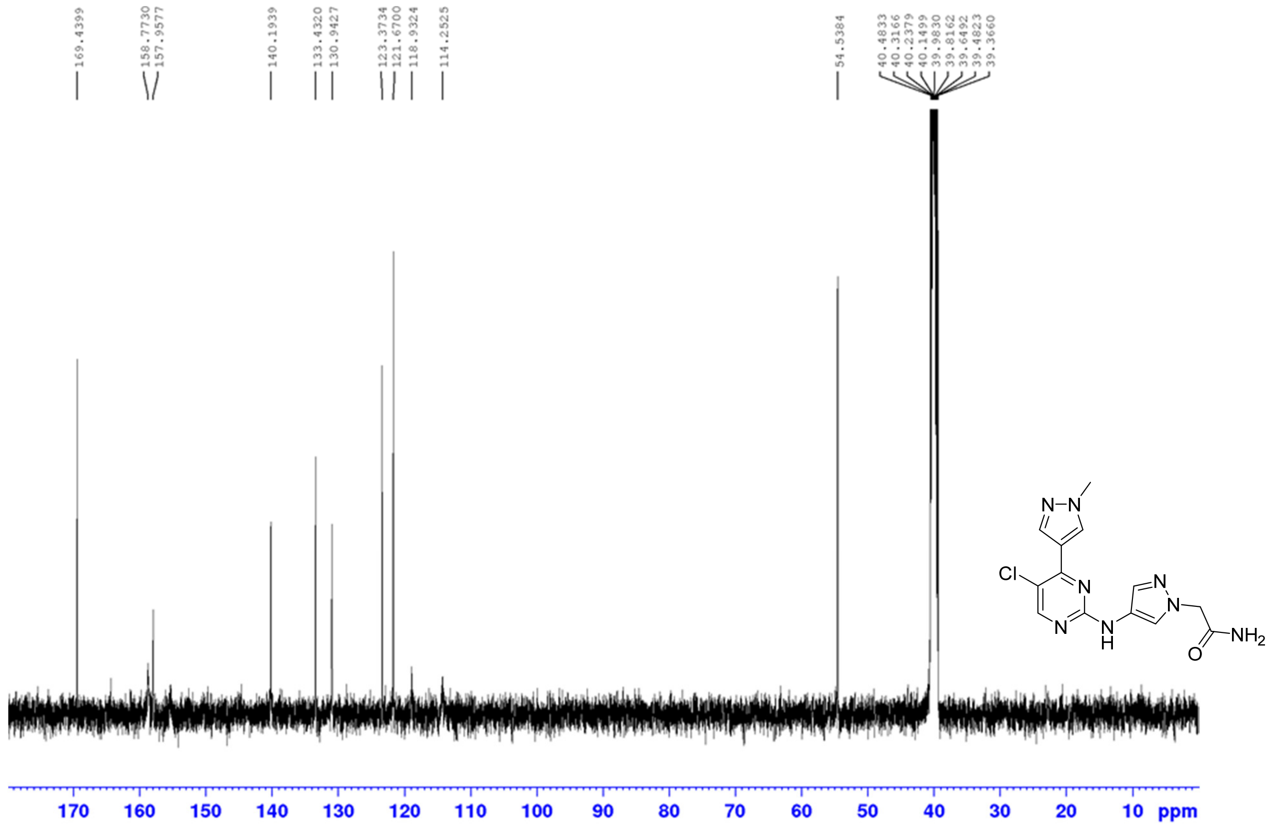

Figure S27. ^{13}C NMR spectrum of **19** in DMSO-d_6 (125 MHz).

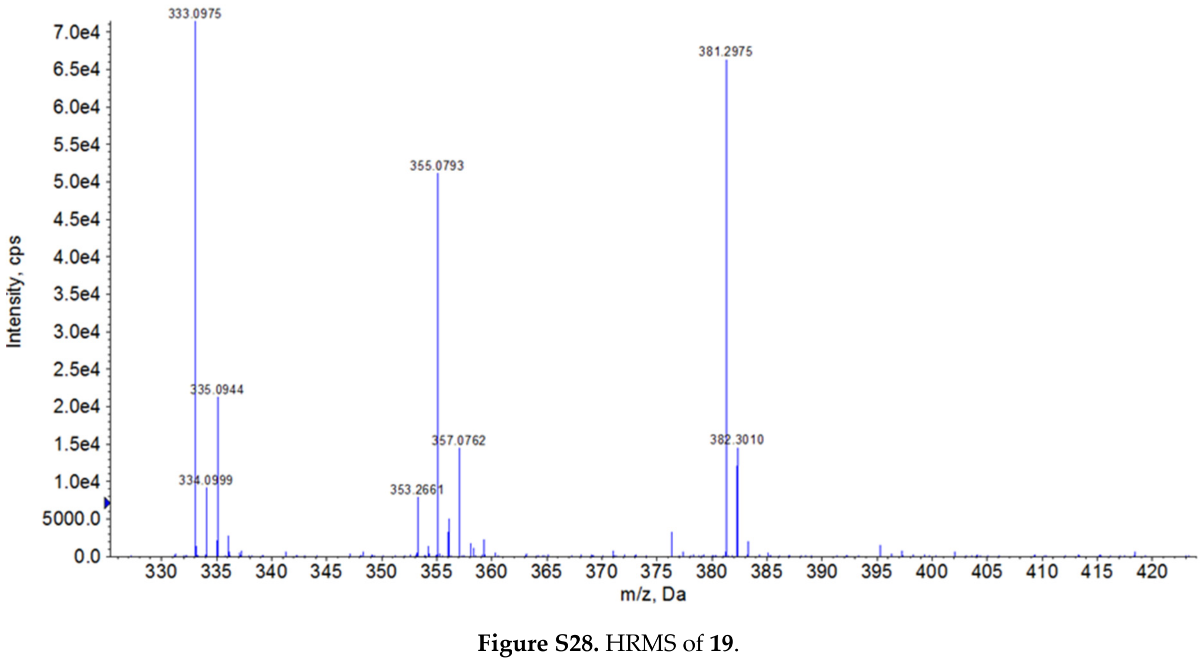

Figure S28. HRMS of **19**.

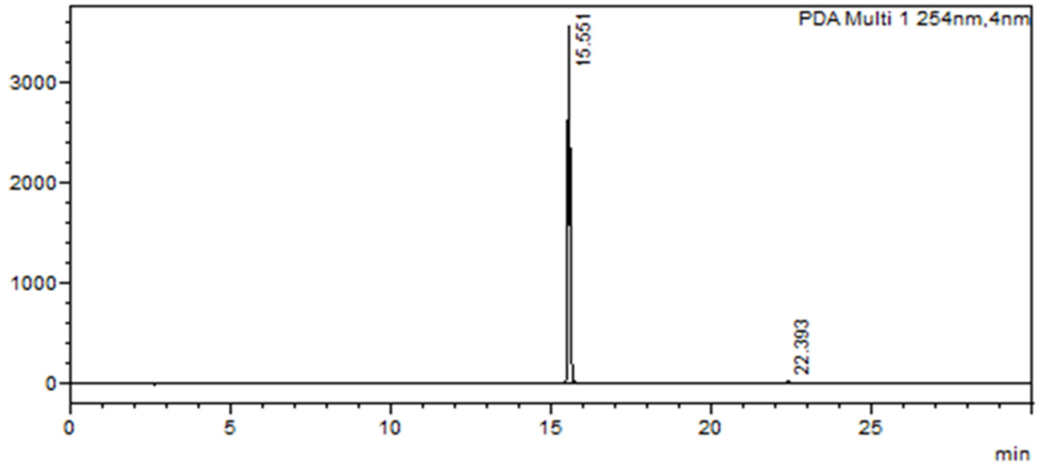

<Peak Table>

PDA Ch1 254nm

Peak#	Ret. Time	Area	Height	Conc.	Unit	Mark	Name
1	15.551	17250054	3558015	98.710			
2	22.393	225410	22741	1.290			
Total		17475464	3580756				

Figure S29. HPLC chromatogram of **19** determined by method A.

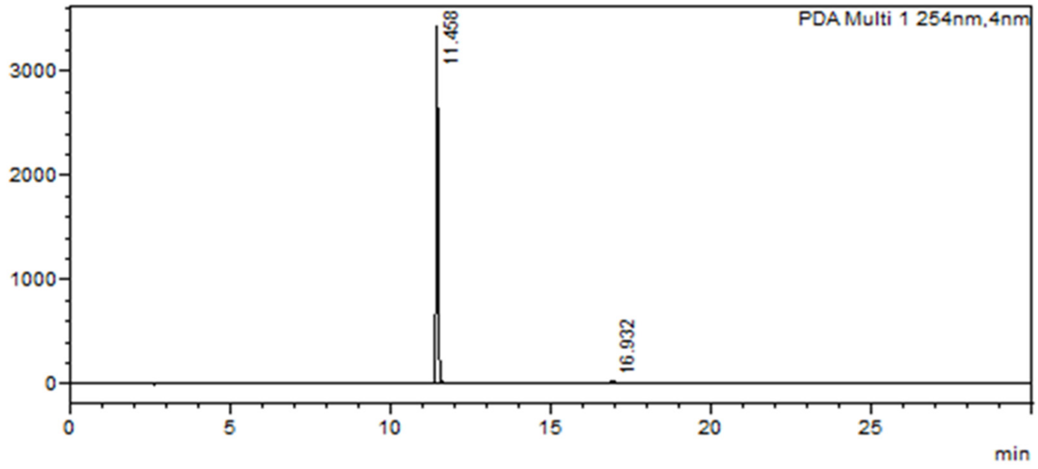

<Peak Table>

PDA Ch1 254nm

Peak#	Ret. Time	Area	Height	Conc.	Unit	Mark	Name
1	11.458	13653653	3420590	98.015			
2	16.932	276454	28686	1.985			
Total		13930106	3449276				

Figure S30. HPLC chromatogram of **19** determined by method B.

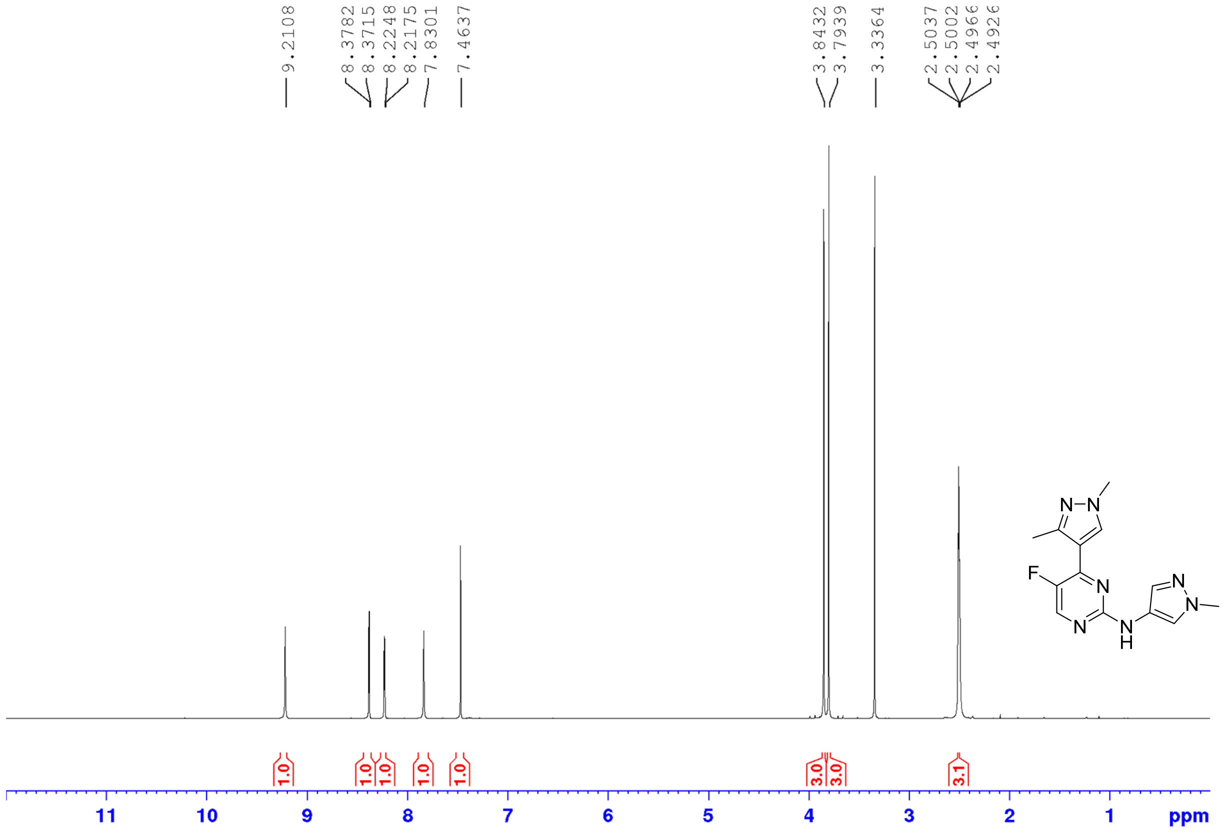

Figure S31. ^{1}H NMR spectrum of **20** in DMSO-d_6 (500 MHz).

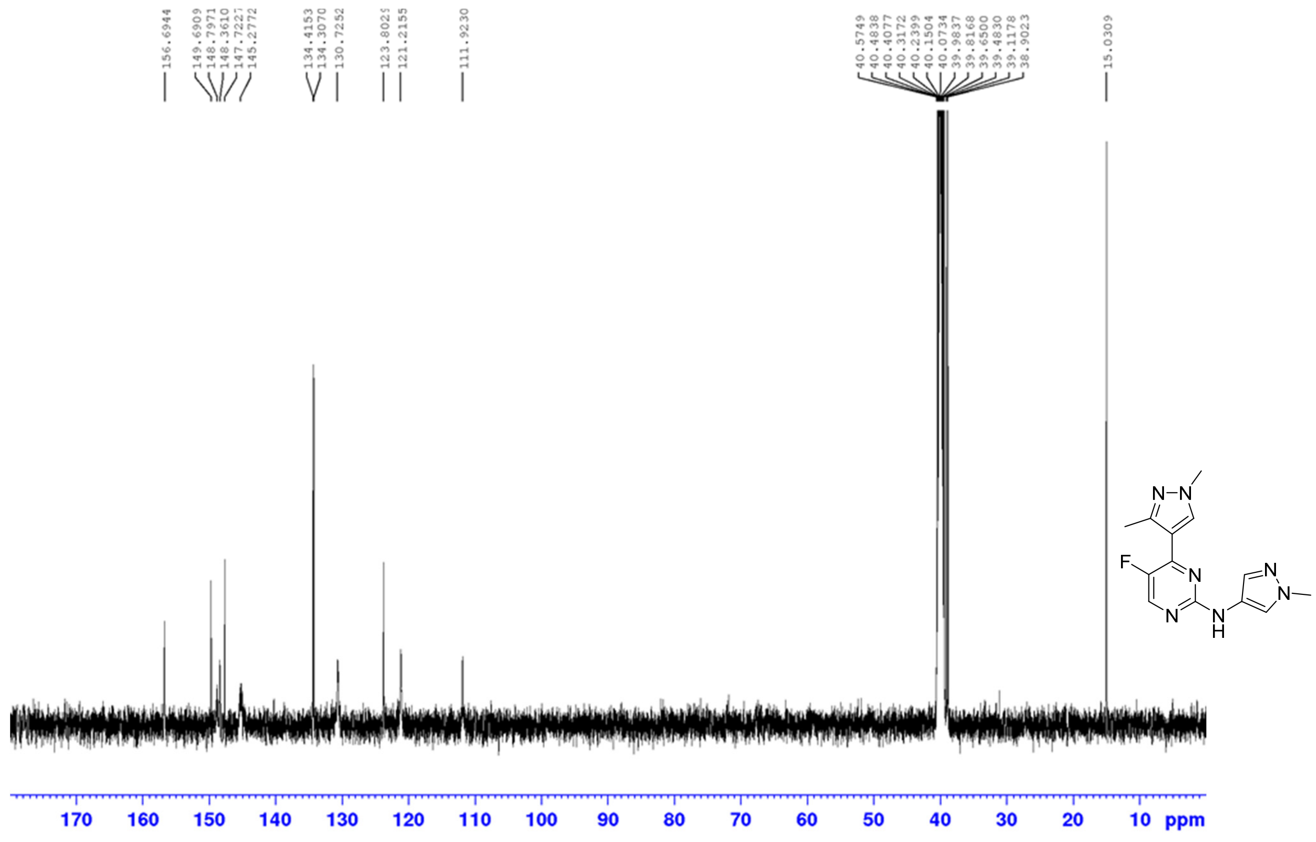

Figure S32. ^{13}C NMR spectrum of **20** in DMSO-d_6 (125 MHz).

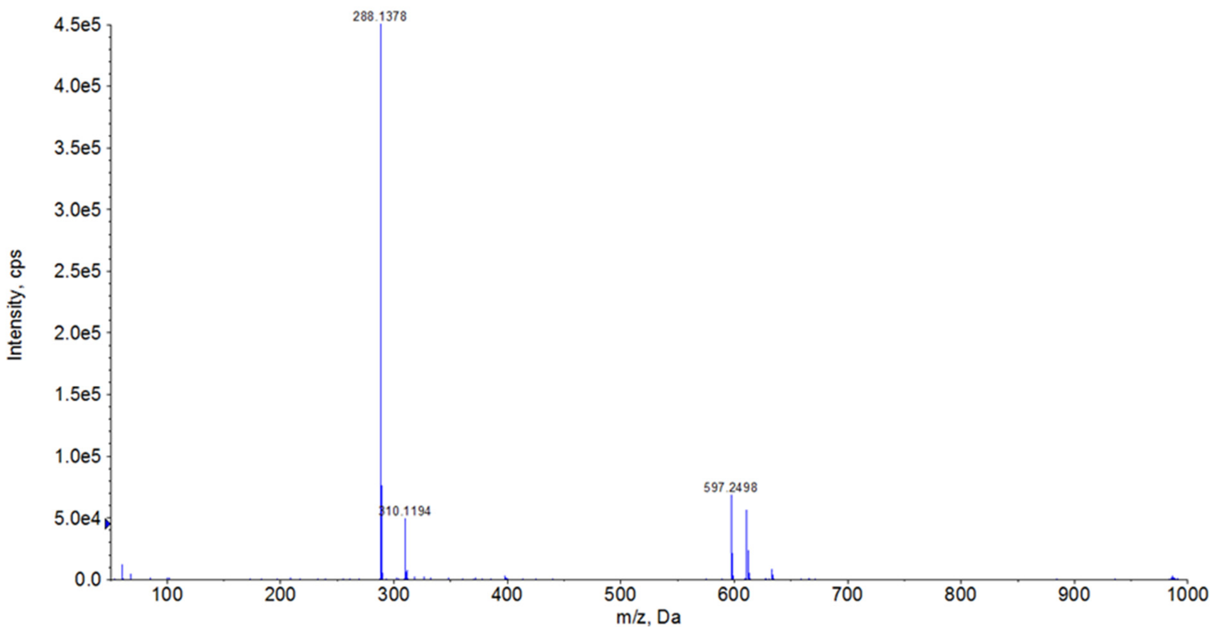

Figure S33. HRMS of **20**.

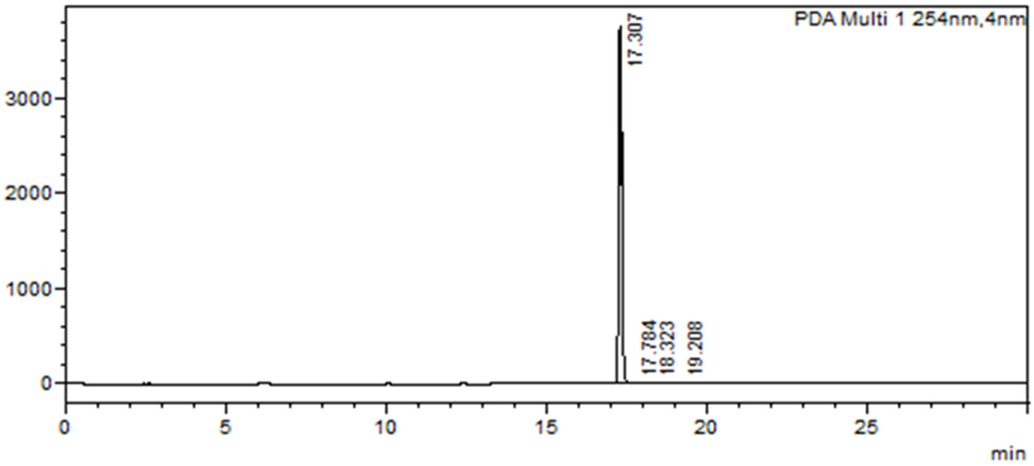

<Peak Table>

PDA Ch1 254nm

Peak#	Ret. Time	Area	Height	Conc.	Unit	Mark	Name
1	17.307	26888821	3741374	99.509			
2	17.784	67519	11908	0.250		V	
3	18.323	38741	8003	0.143			
4	19.208	26320	5714	0.097			
Total		27021403	3766999				

Figure S34. HPLC chromatogram of **20** determined by method A.

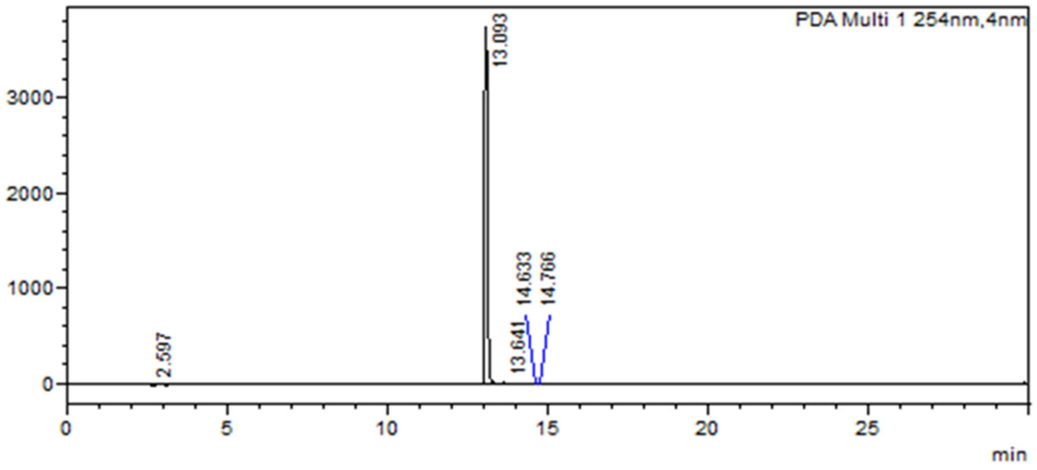

<Peak Table>

PDA Ch1 254nm

Peak#	Ret. Time	Area	Height	Conc.	Unit	Mark	Name
1	2.597	28220	9603	0.116			
2	13.093	24160213	3736673	99.377			
3	13.641	58547	11999	0.241		V	
4	14.633	34318	7906	0.141			
5	14.766	30279	6761	0.125		V	
Total		24311578	3772942				

Figure S35. HPLC chromatogram of **20** determined by method B.

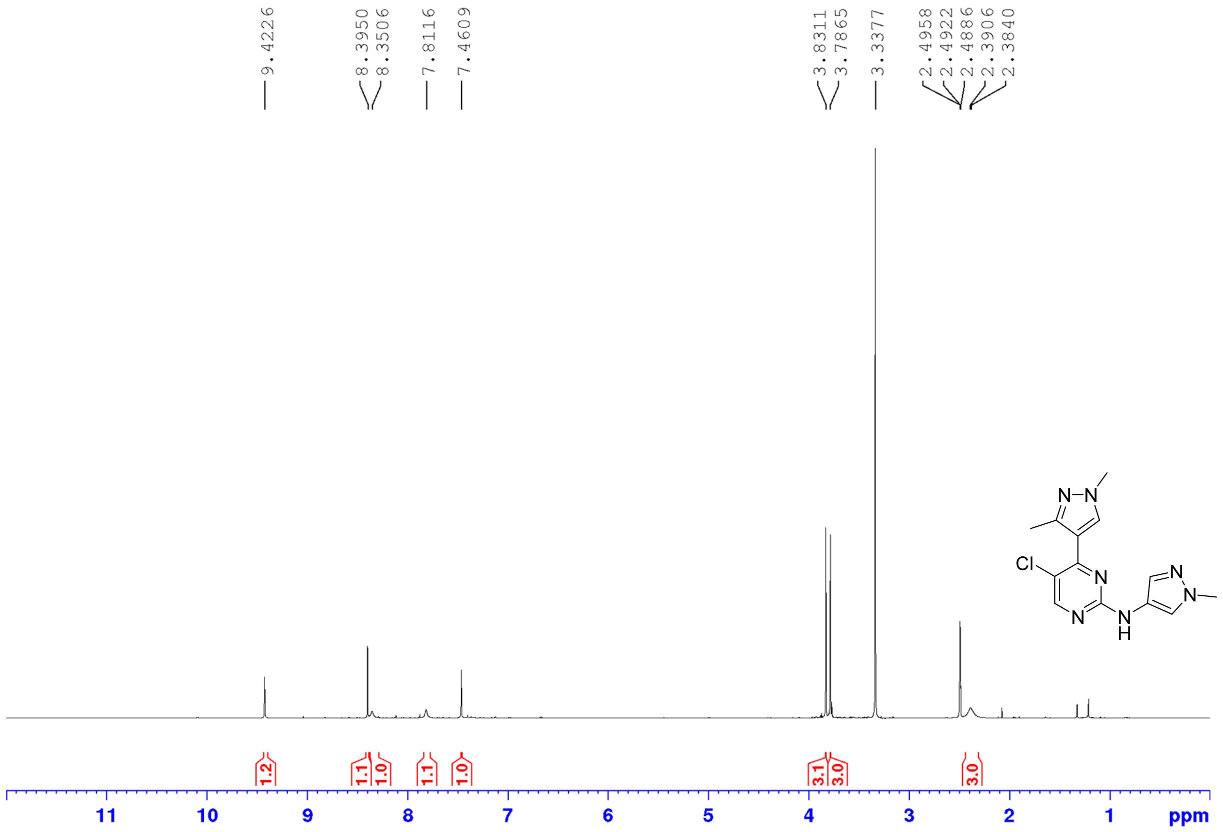

Figure S36. ^{1}H NMR spectrum of **21** in DMSO-d_6 (500 MHz).

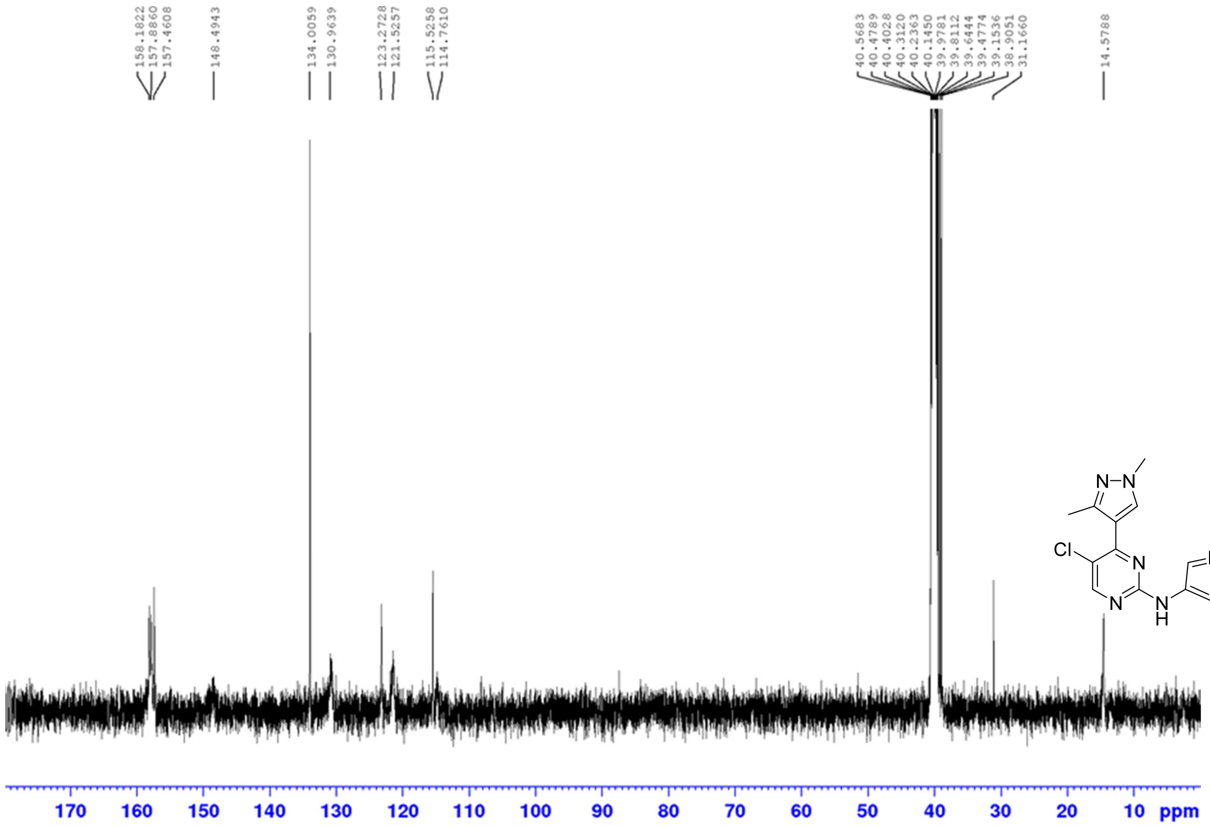

Figure S37. ^{13}C NMR spectrum of **21** in DMSO-d_6 (125 MHz).

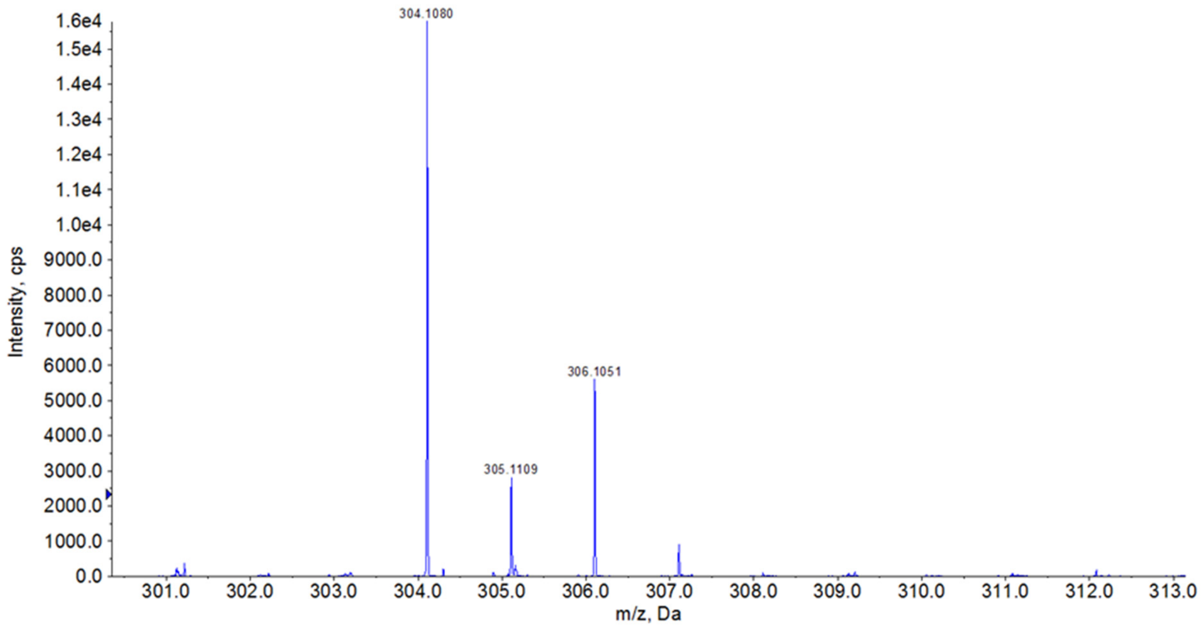

Figure S38. HRMS of **21**.

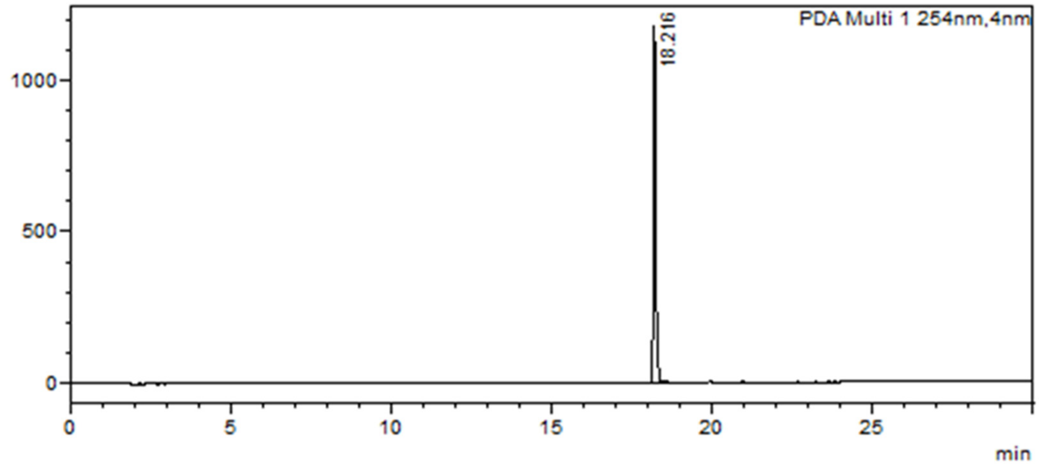

<Peak Table>

PDA Ch1 254nm

Peak#	Ret. Time	Area	Height	Conc.	Unit	Mark	Name
1	18.216	5934491	1173601	100.000			
Total		5934491	1173601				

Figure S39. HPLC chromatogram of **21** determined by method A.

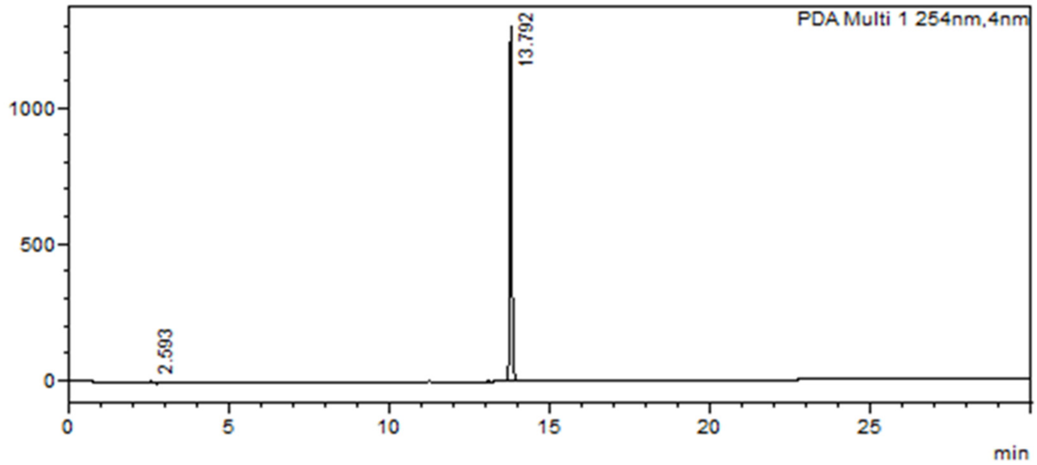

<Peak Table>

PDA Ch1 254nm

Peak#	Ret. Time	Area	Height	Conc.	Unit	Mark	Name
1	2.593	29866	10979	0.524			
2	13.792	5673906	1298775	99.476			
Total		5703772	1309754				

Figure S40. HPLC chromatogram of **21** determined by method B.

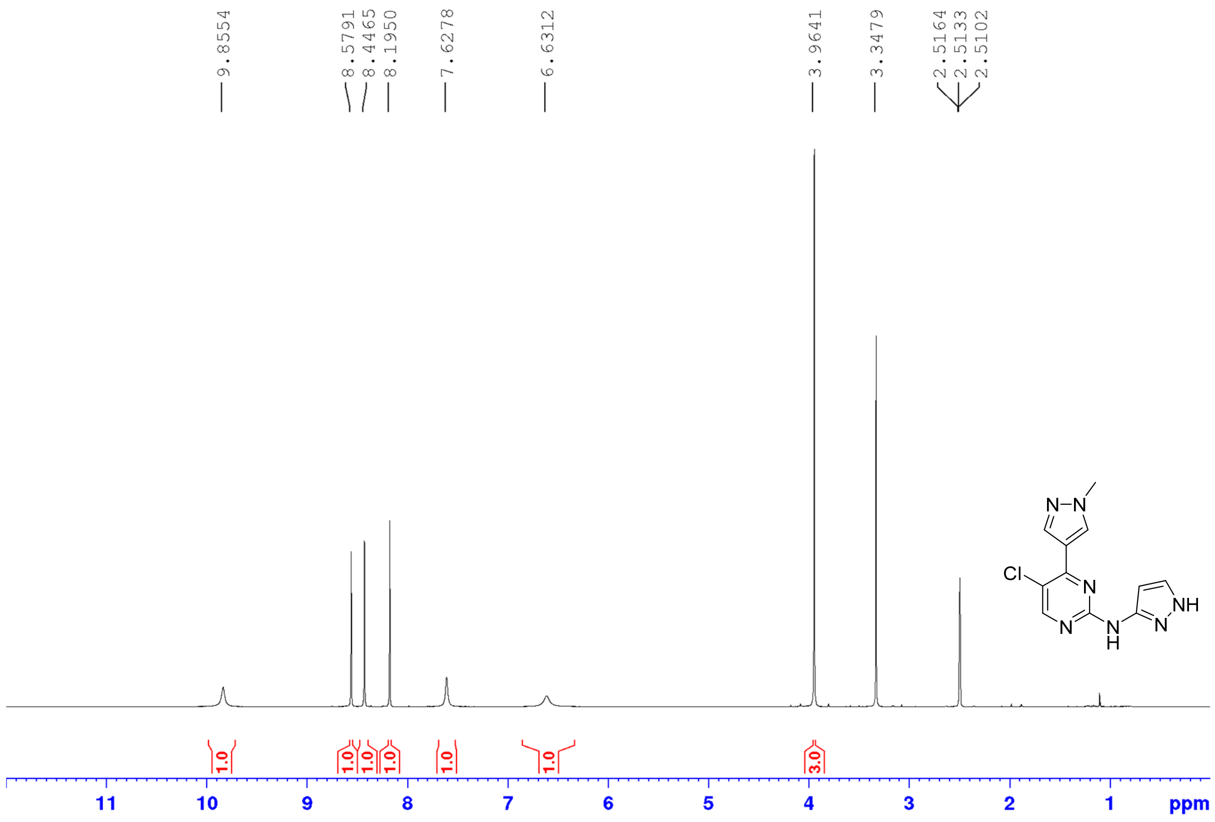

Figure S41. ^{1}H NMR spectrum of **23** in DMSO-d_6 (500 MHz).

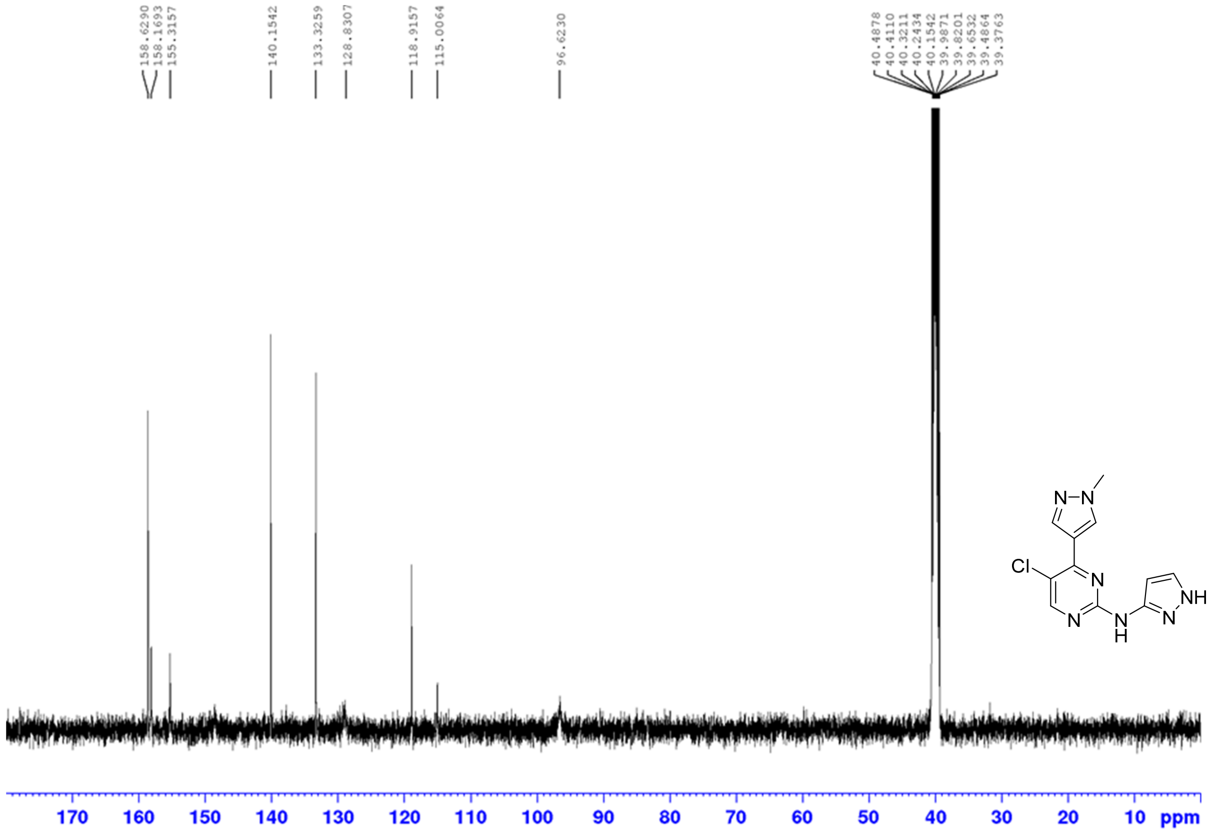

Figure S42. ^{13}C NMR spectrum of **23** in DMSO-d_6 (125 MHz).

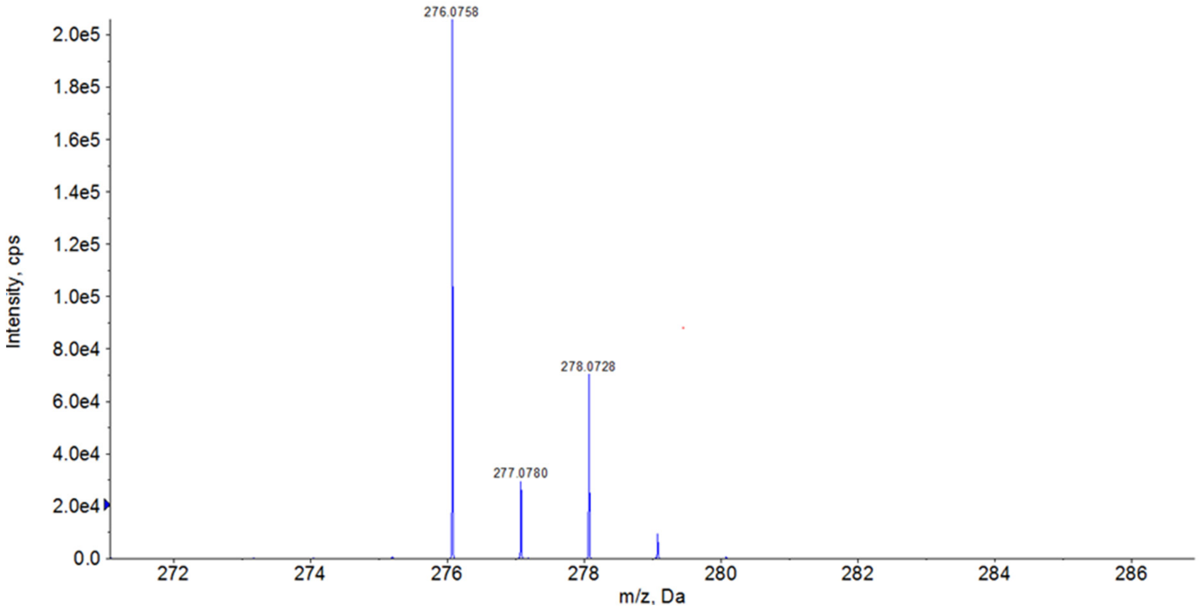

Figure S43. HRMS of **23**.

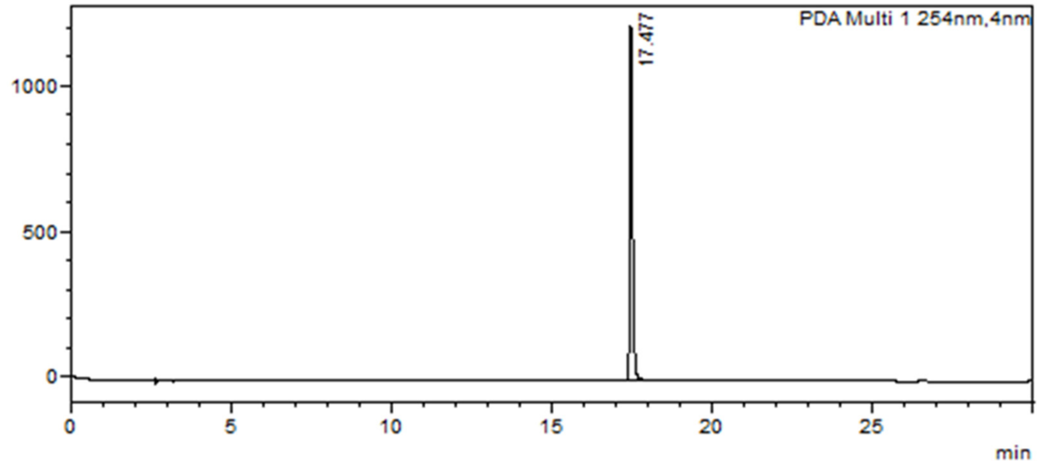

<Peak Table>

PDA Ch1 254nm

Peak#	Ret. Time	Area	Height	Conc.	Unit	Mark	Name
1	17.477	6660849	1213114	100.000			
Total		6660849	1213114				

Figure S44. HPLC chromatogram of **23** determined by method A.

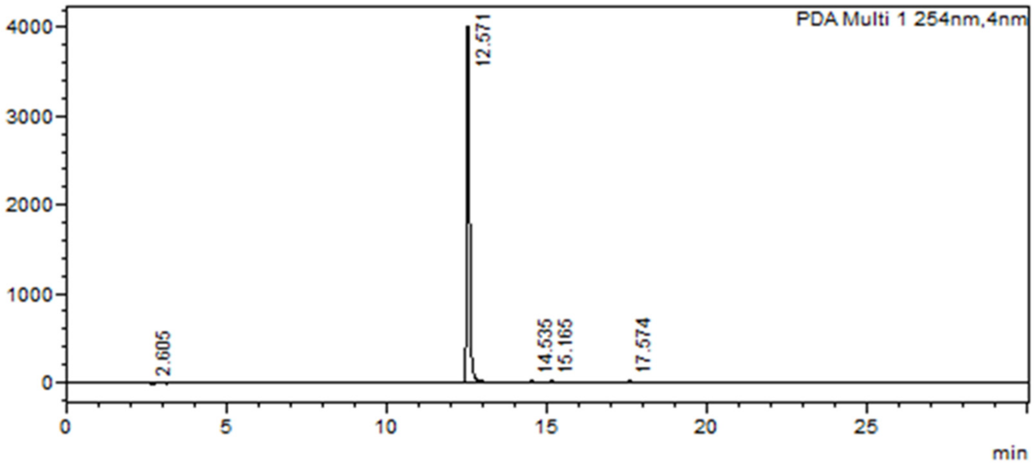

<Peak Table>

PDA Ch1 254nm

Peak#	Ret. Time	Area	Height	Conc.	Unit	Mark	Name
1	2.605	41583	18159	0.142			
2	12.571	28841977	4002782	98.700			
3	14.535	81708	14595	0.280			
4	15.165	95632	18969	0.327			
5	17.574	160834	23528	0.550			
Total		29221734	4078032				

Figure S45. HPLC chromatogram of **23** determined by method B.

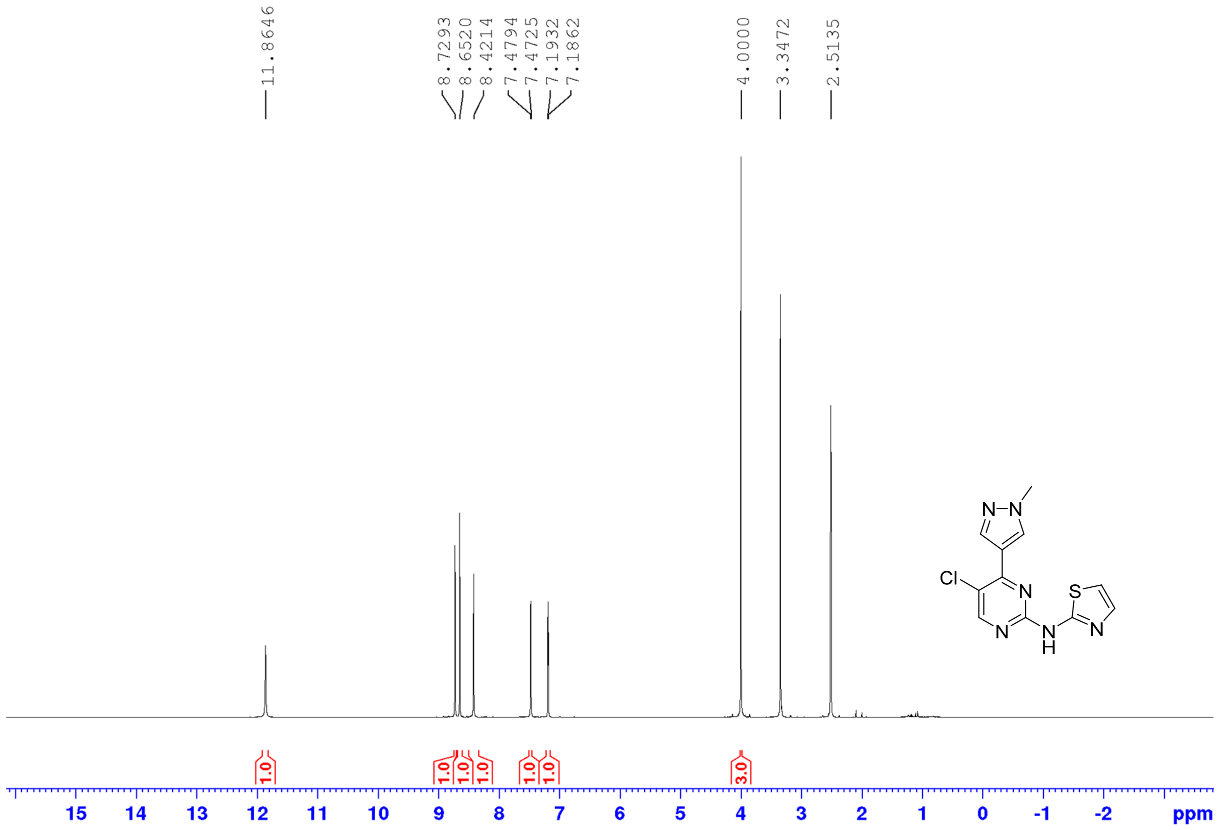

Figure S46. ^{1}H NMR spectrum of **25** in DMSO-d_6 (500 MHz).

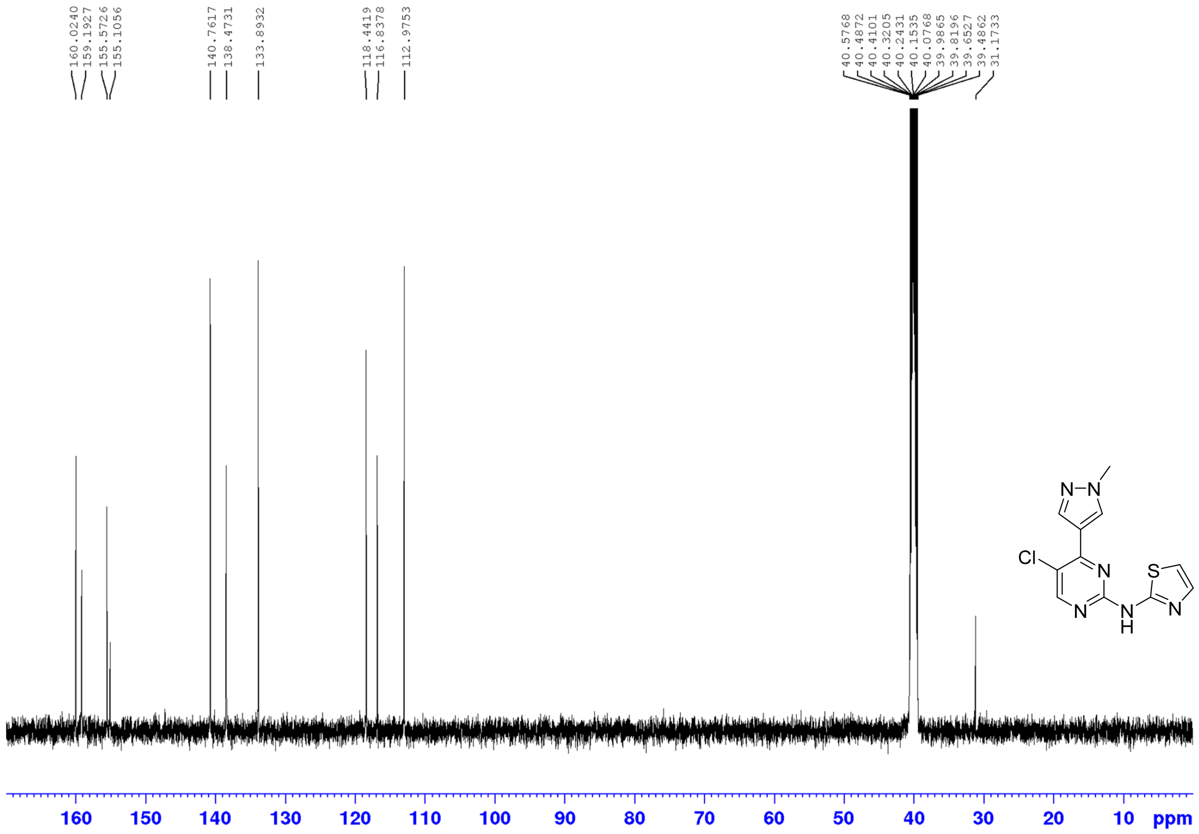

Figure S47. ^{13}C NMR spectrum of **25** in DMSO-d_6 (125 MHz).

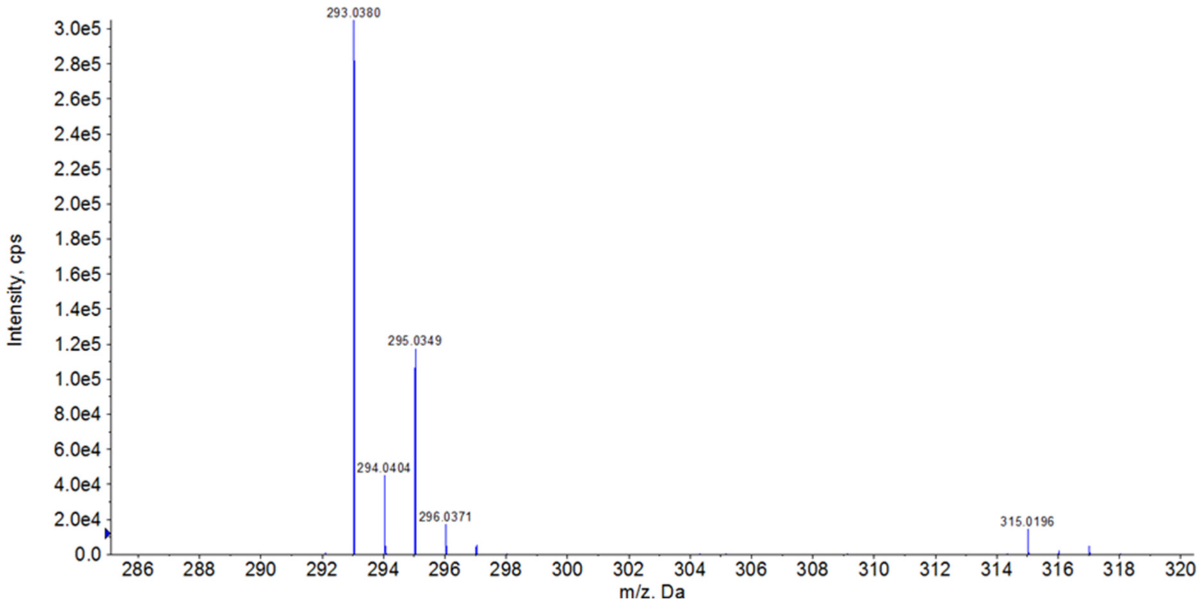

Figure S48. HRMS of **25**.

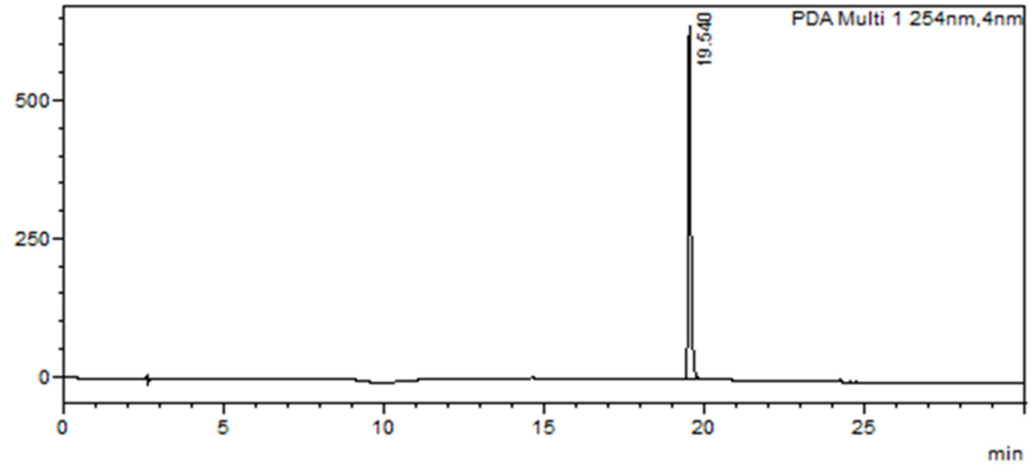

<Peak Table>

PDA Ch1 254nm

Peak#	Ret. Time	Area	Height	Conc.	Unit	Mark	Name
1	19.540	3463948	637472	100.000			
Total		3463948	637472				

Figure S49. HPLC chromatogram of **25** determined by method A.

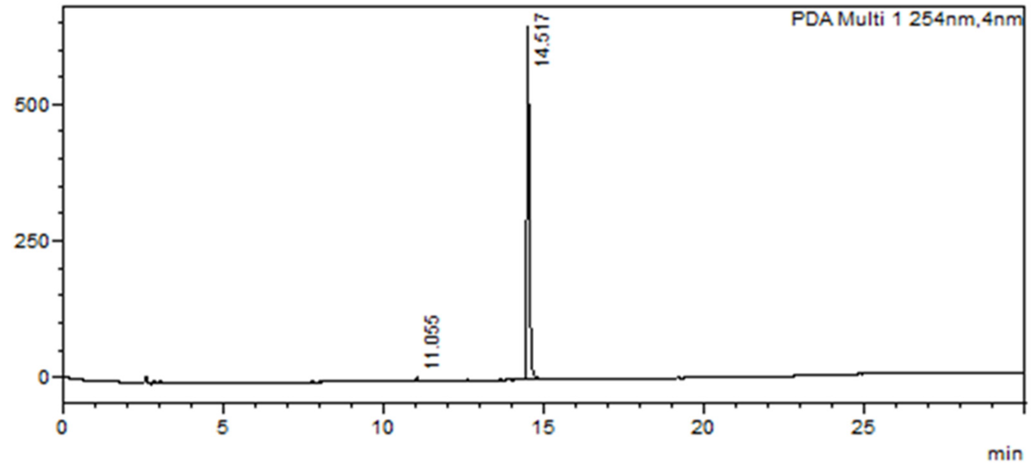

<Peak Table>

PDA Ch1 254nm

Peak#	Ret. Time	Area	Height	Conc.	Unit	Mark	Name
1	11.055	25561	6084	0.782			
2	14.517	3243477	644404	99.218			
Total		3269038	650488				

Figure S50. HPLC chromatogram of **25** determined by method B.

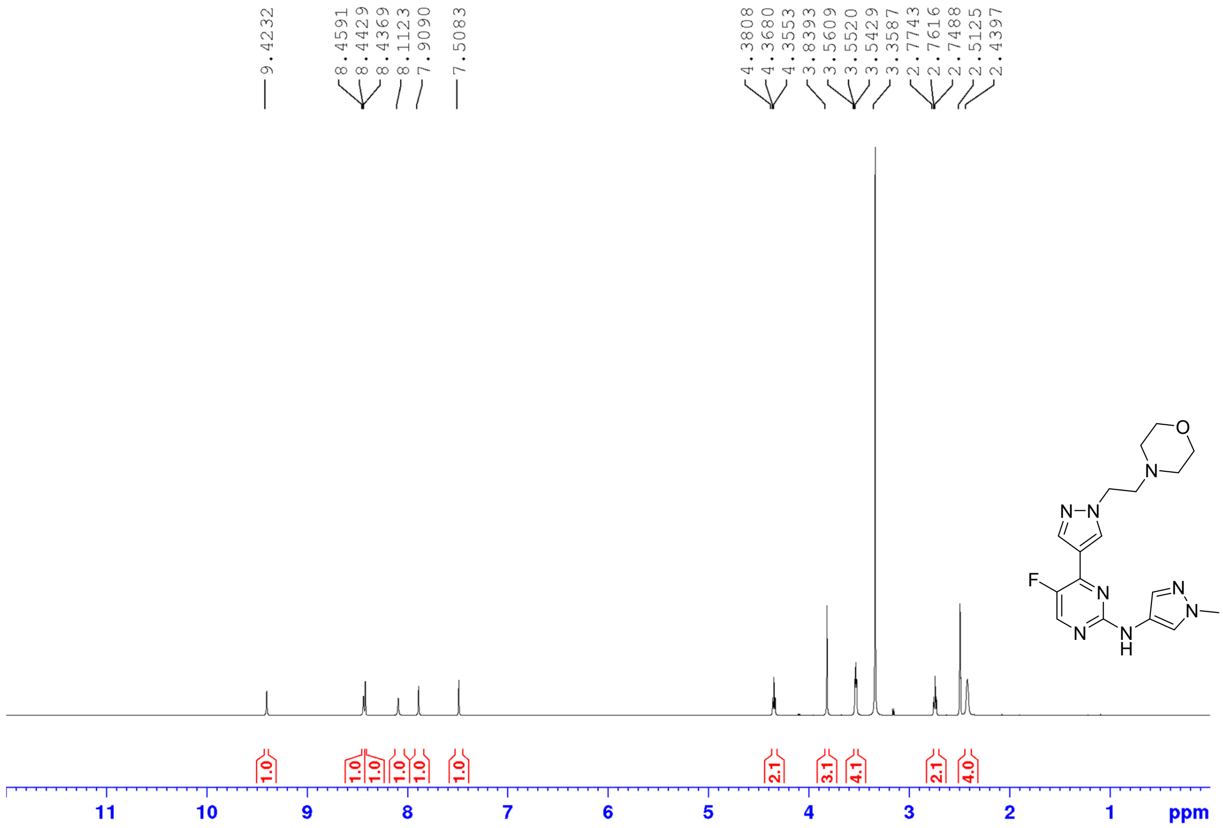

Figure S51. ^{1}H NMR spectrum of **31** in DMSO-d_6 (500 MHz).

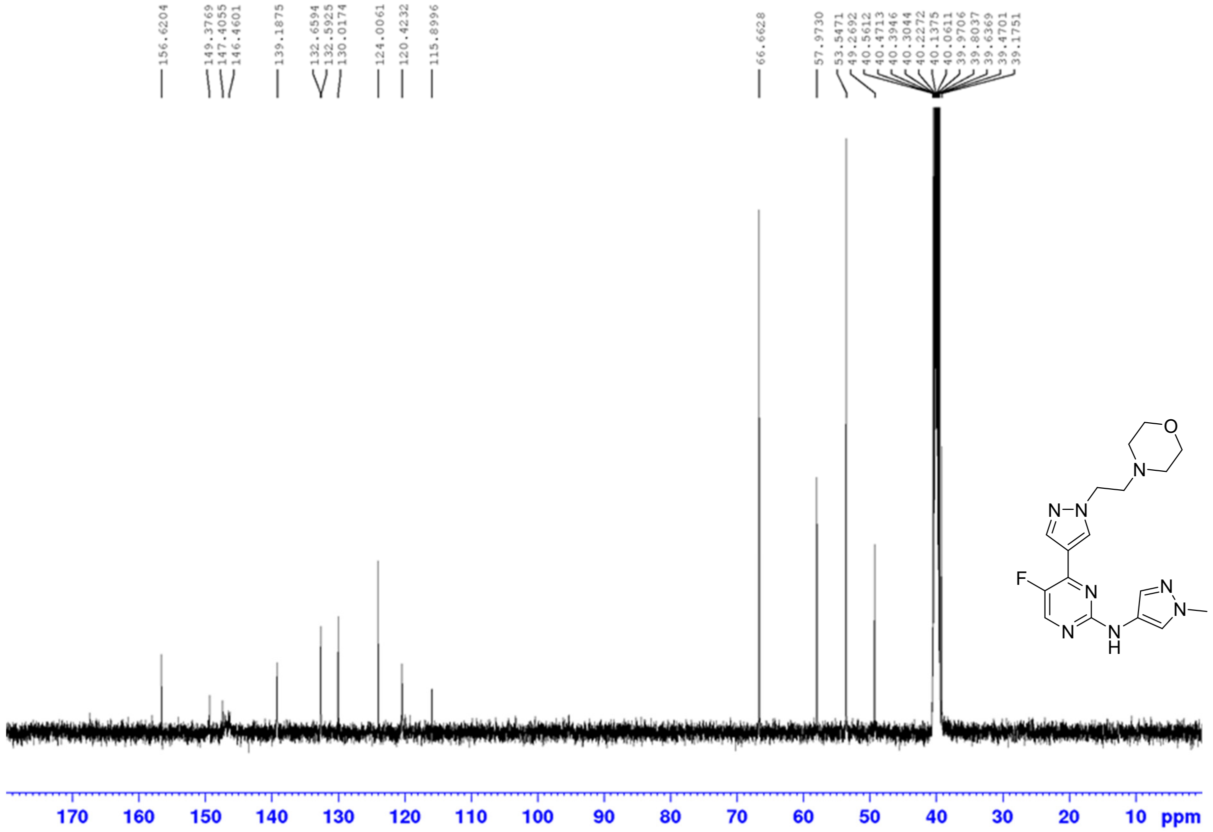

Figure S52. ^{13}C NMR spectrum of **31** in DMSO-d_6 (125 MHz).

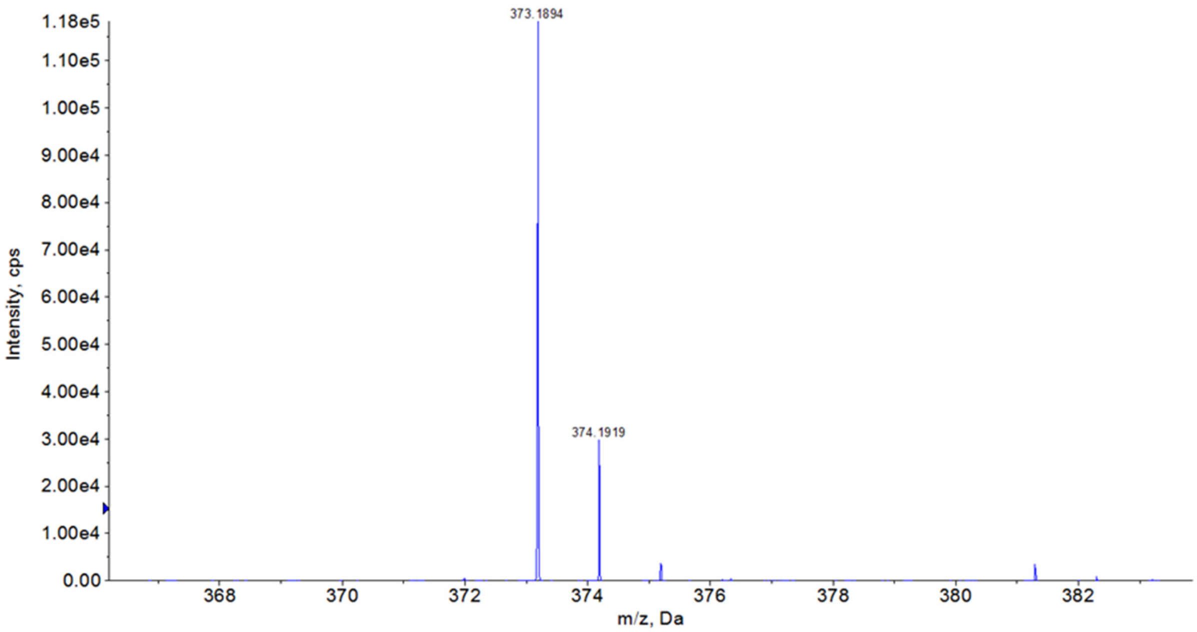

Figure S53. HRMS of **31**.

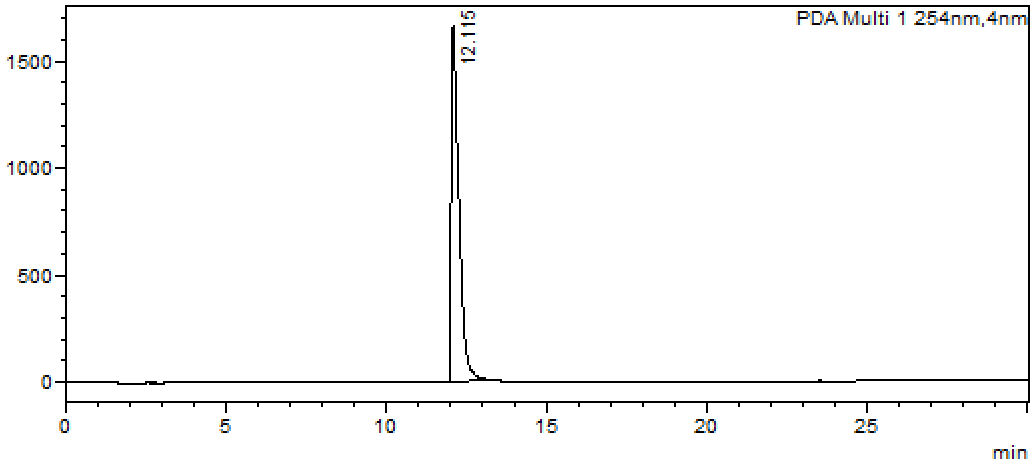

<Peak Table>

PDA Ch1 254nm

Peak#	Ret. Time	Area	Height	Conc.	Unit	Mark	Name
1	12.115	27865018	1665937	100.000			
Total		27865018	1665937				

Figure S54. HPLC chromatogram of **31** determined by method A.

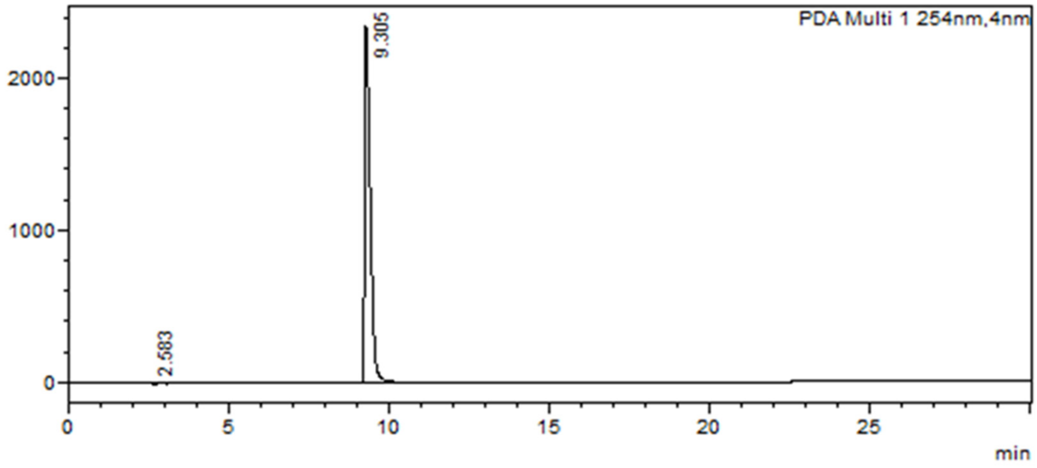

<Peak Table>

PDA Ch1 254nm

Peak#	Ret. Time	Area	Height	Conc.	Unit	Mark	Name
1	2.583	31629	10395	0.114			
2	9.305	27748824	2341917	99.886			
Total		27780453	2352313				

Figure S55. HPLC chromatogram of **31** determined by method B.

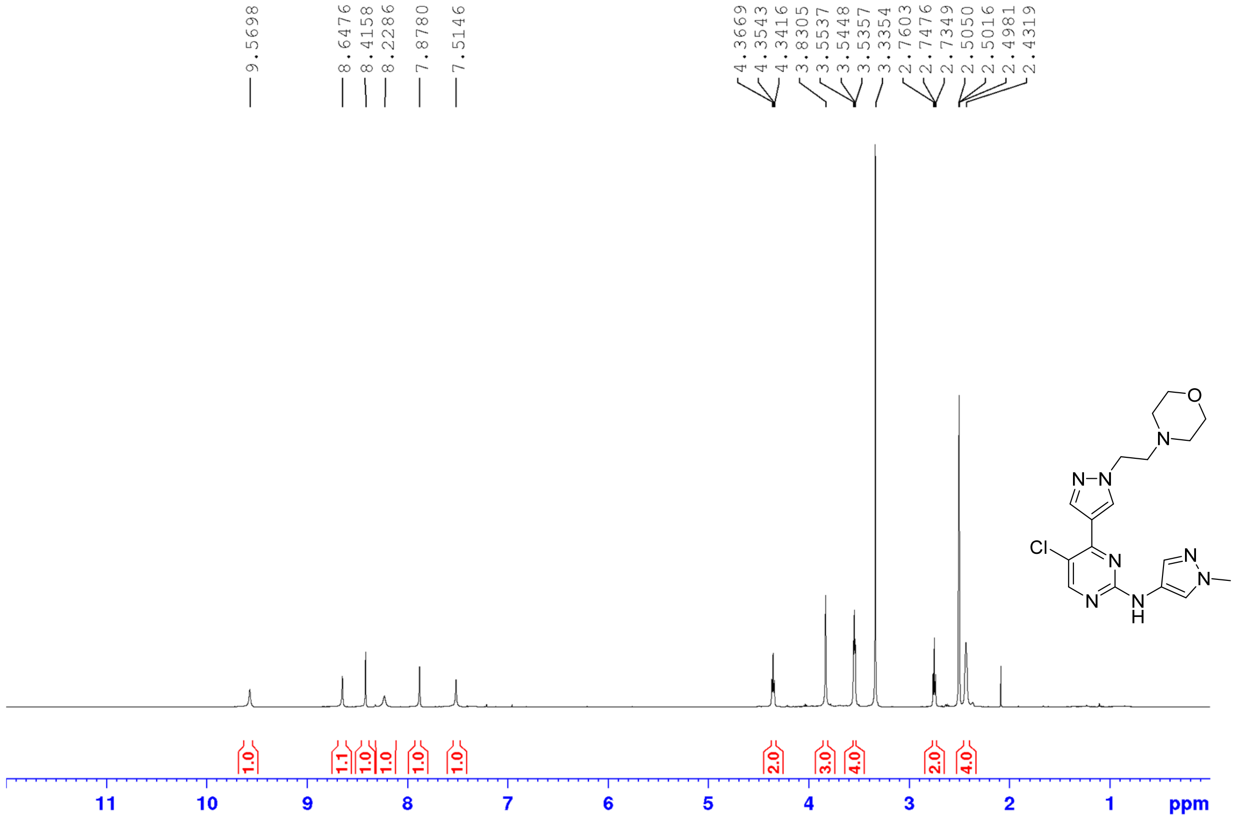

Figure S56. ^{1}H NMR spectrum of **32** in DMSO-d_6 (500 MHz).

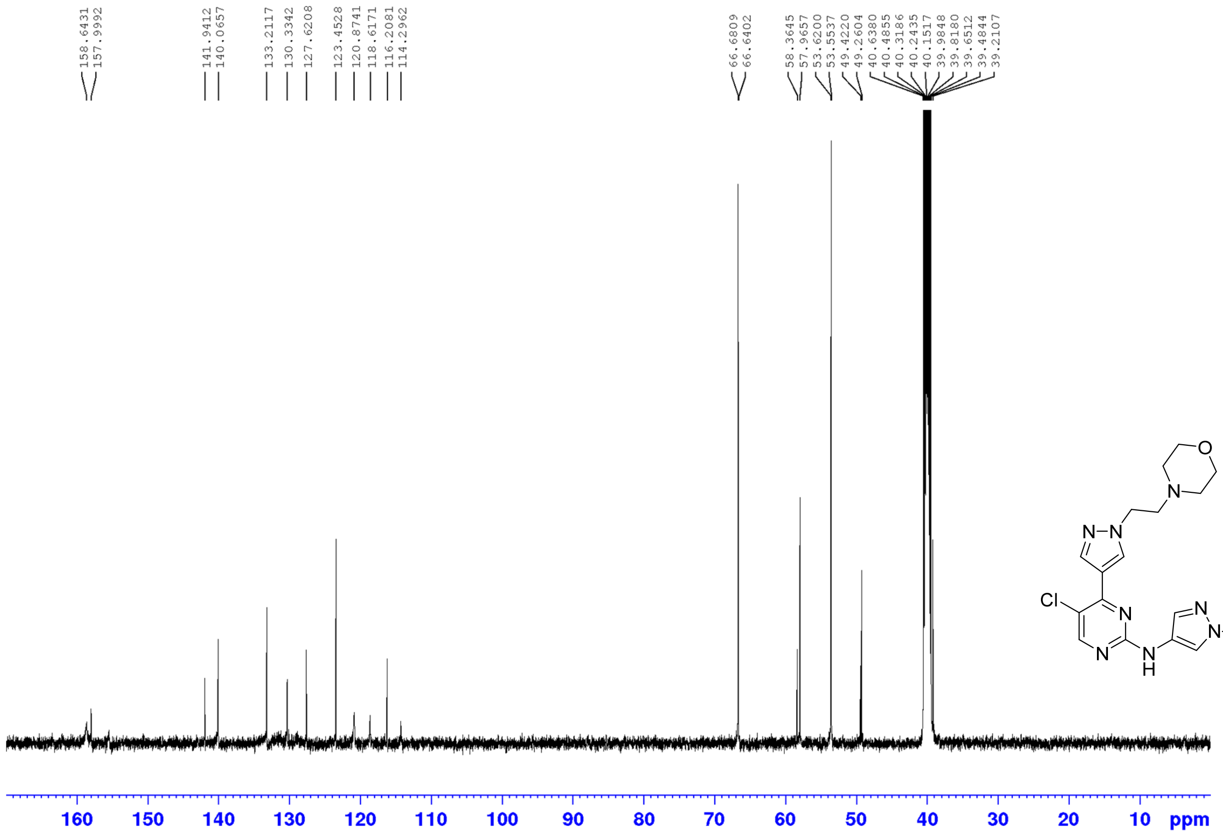

Figure S57. ^{13}C NMR spectrum of **32** in DMSO-d_6 (125 MHz).

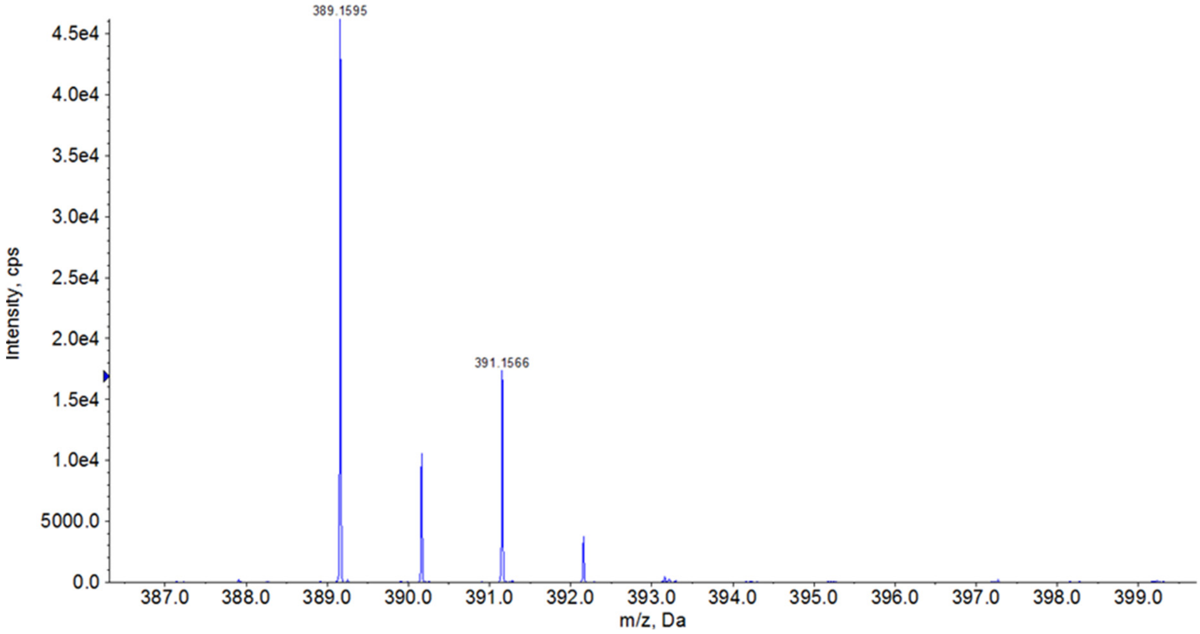

Figure S58. HRMS of **32**.

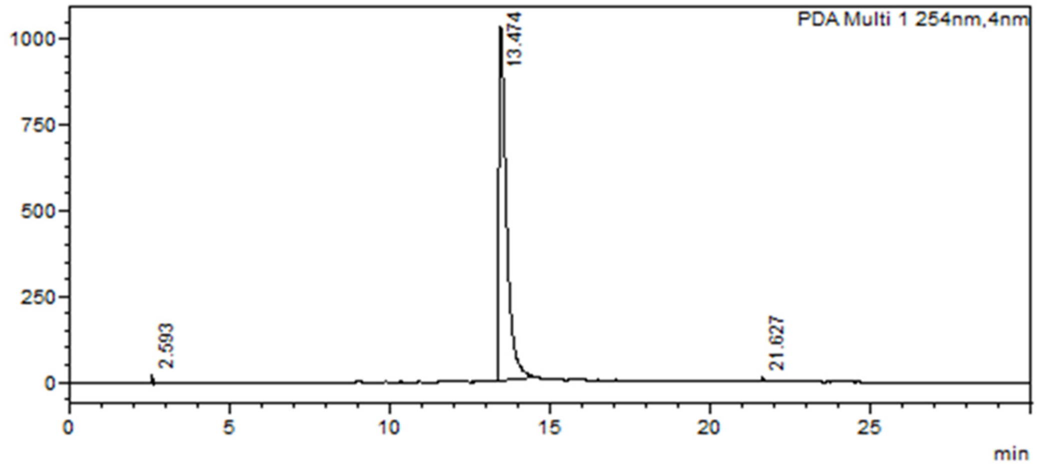

<Peak Table>

PDA Ch1 254nm

Peak#	Ret. Time	Area	Height	Conc.	Unit	Mark	Name
1	2.593	63476	23523	0.368			
2	13.474	17118563	1030230	99.296			
3	21.627	57885	12894	0.336			
Total		17239924	1066648				

Figure S59. HPLC chromatogram of **32** determined by method A.

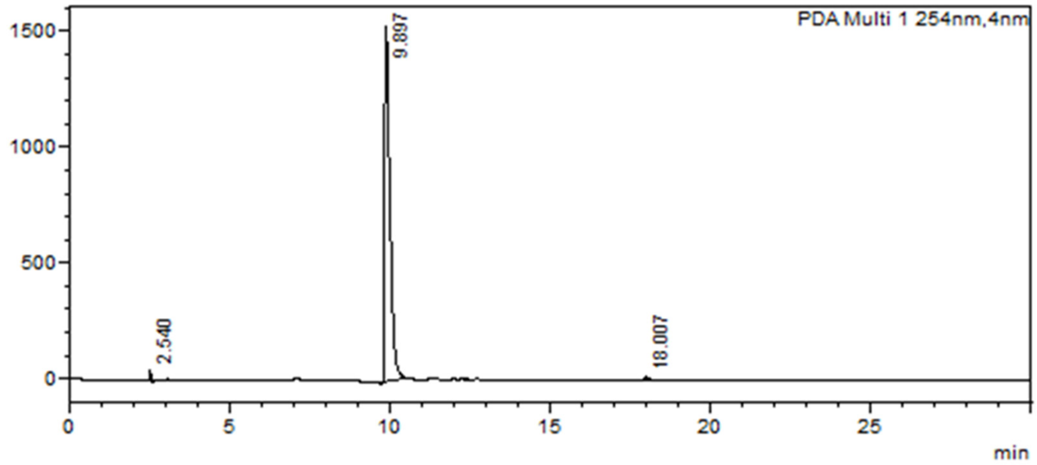

<Peak Table>

PDA Ch1 254nm

Peak#	Ret. Time	Area	Height	Conc.	Unit	Mark	Name
1	2.540	145491	46706	0.824			
2	9.897	17457535	1533616	98.890			
3	18.007	50510	9384	0.286			
Total		17653537	1589705				

Figure S60. HPLC chromatogram of **32** determined by method B.

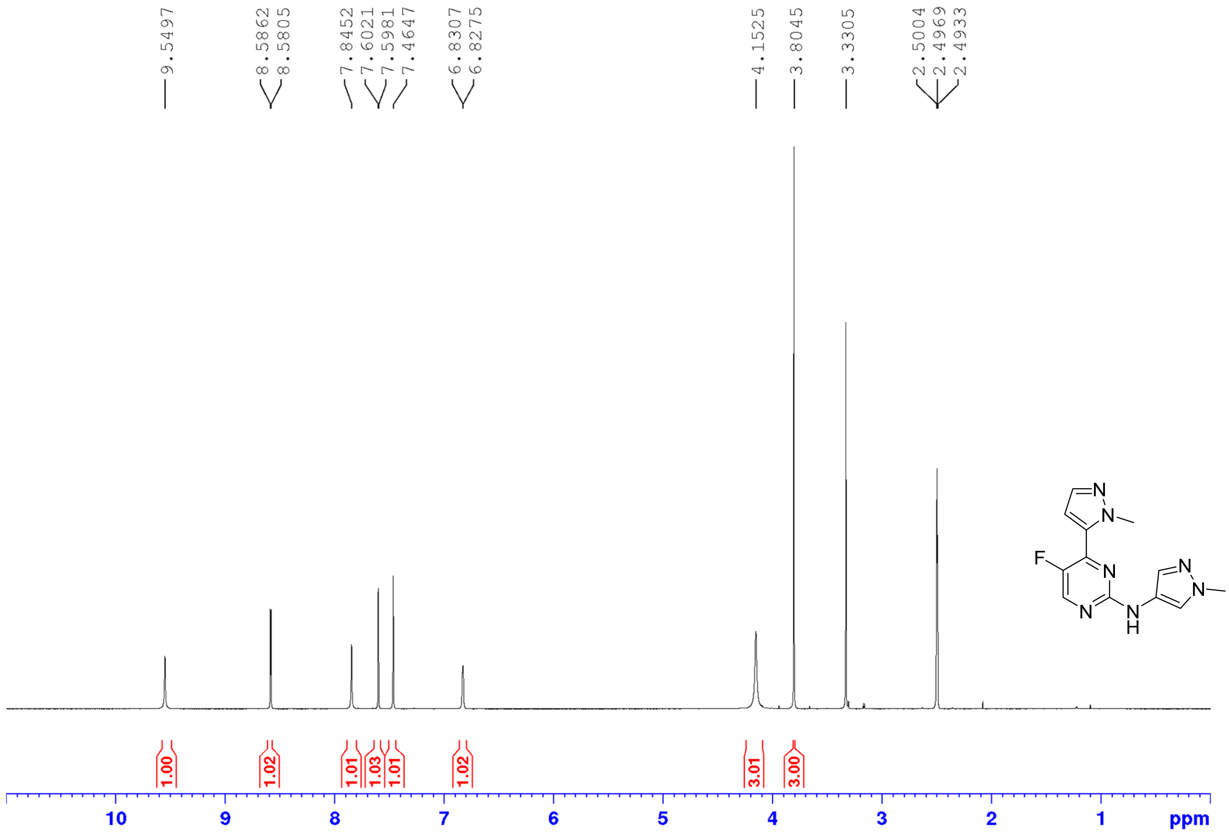

Figure S61. ^{1}H NMR spectrum of **35** in DMSO-d_6 (500 MHz).

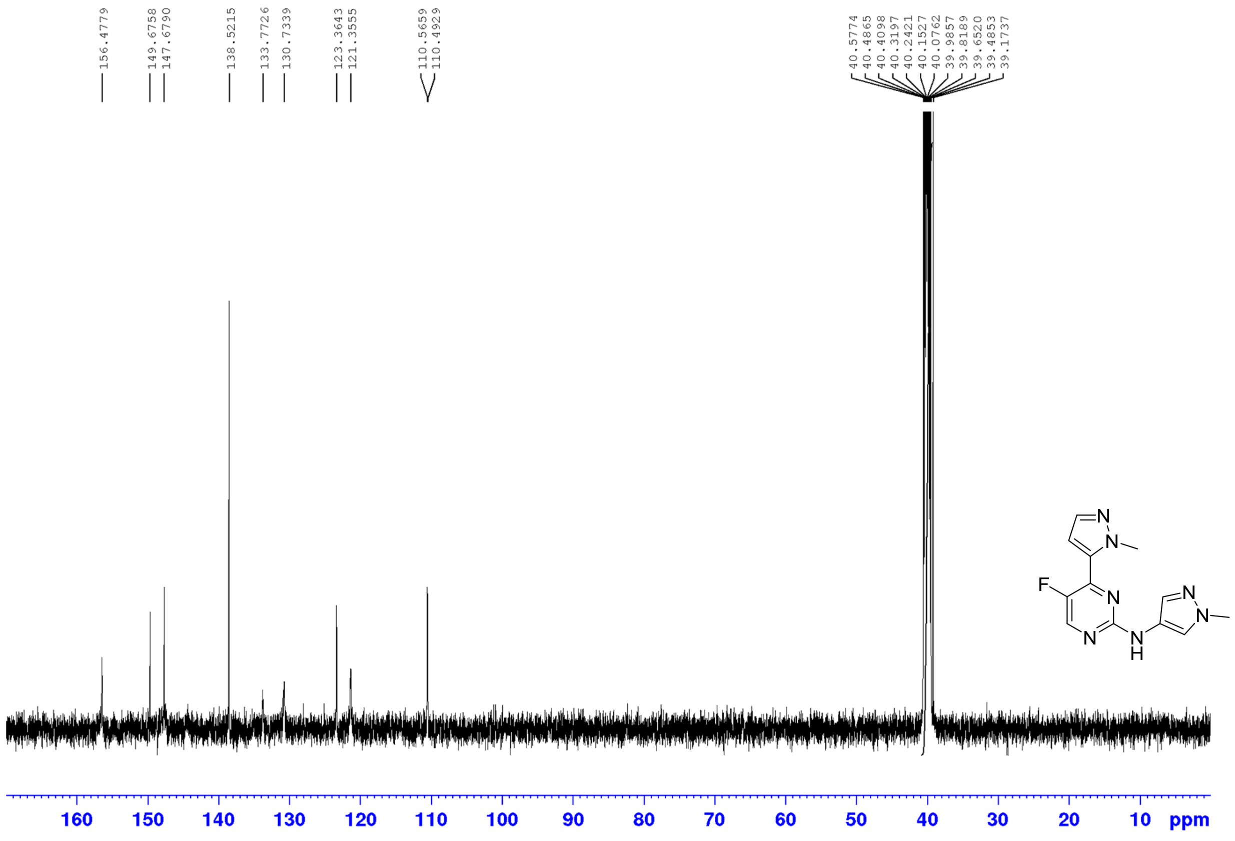

Figure S62. ^{13}C NMR spectrum of **35** in DMSO-d_6 (125 MHz).

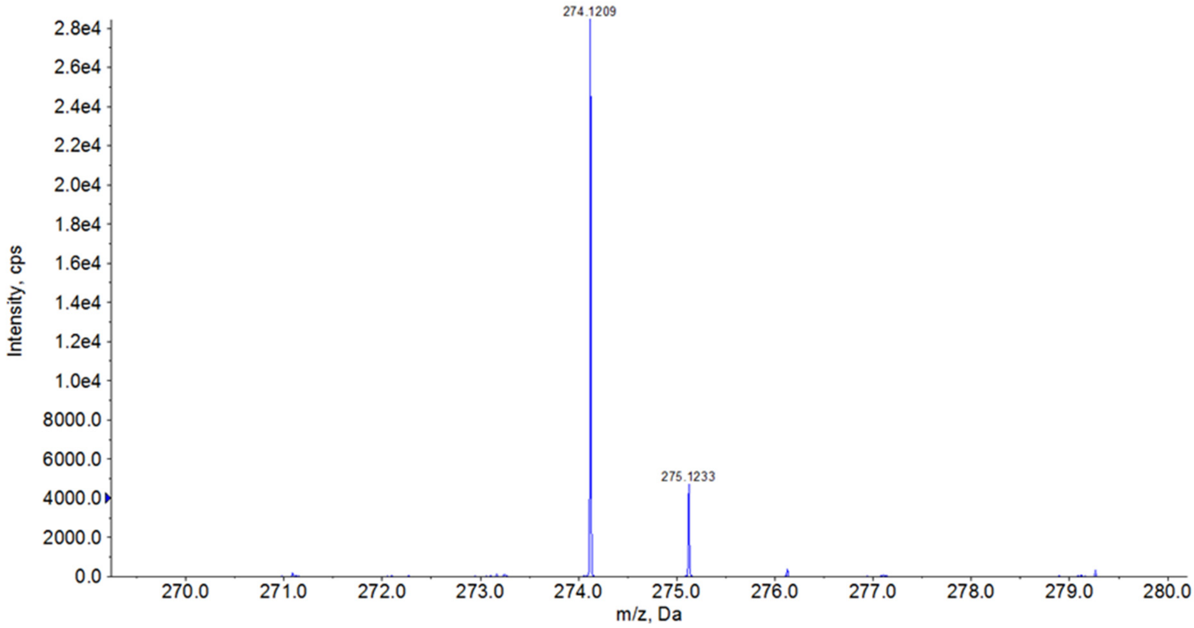

Figure S63. HRMS of **35**.

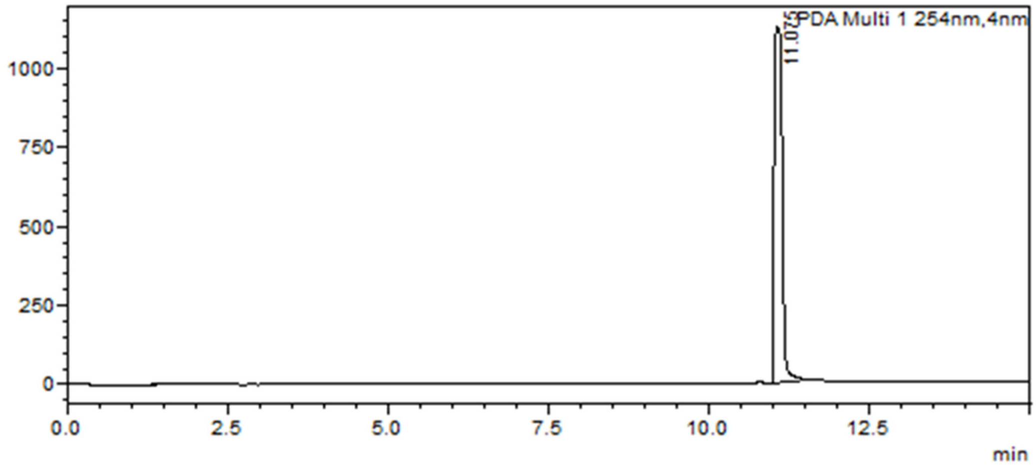

<Peak Table>

PDA Ch1 254nm

Peak#	Ret. Time	Area	Height	Conc.	Unit	Mark	Name
1	11.075	10009115	1123706	100.000			
Total		10009115	1123706				

Figure S64. HPLC chromatogram of **35** determined by method A.

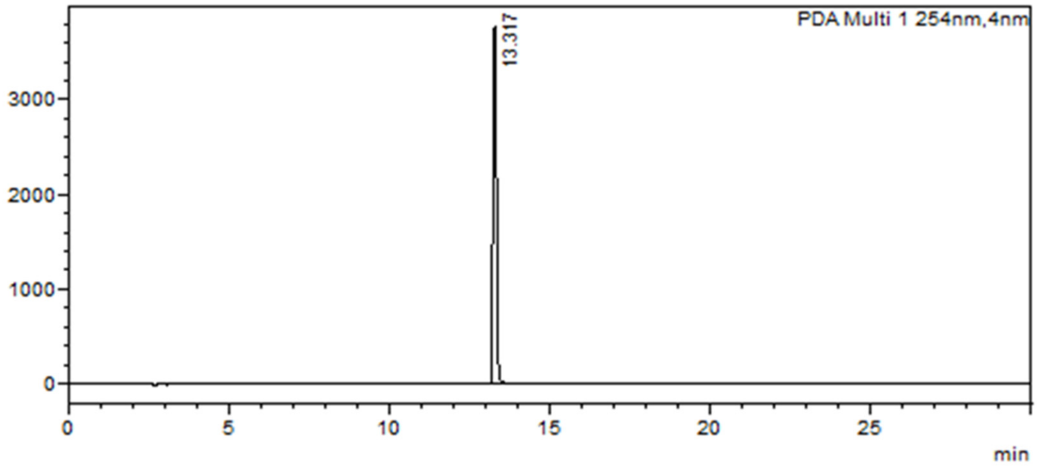

<Peak Table>

PDA Ch1 254nm

Peak#	Ret. Time	Area	Height	Conc.	Unit	Mark	Name
1	13.317	28404258	3763998	100.000			
Total		28404258	3763998				

Figure S65. HPLC chromatogram of **35** determined by method B.
